# Quantifying predictors for the spatial diffusion of avian influenza virus in China

**DOI:** 10.1186/s12862-016-0845-3

**Published:** 2017-01-13

**Authors:** Lu Lu, Andrew J. Leigh Brown, Samantha J. Lycett

**Affiliations:** 1 Institute of Evolutionary Biology, Ashworth Laboratories, University of Edinburgh, Edinburgh, EH9 3JT UK; 2 The Roslin Institute, University of Edinburgh, Easter Bush Campus, Midlothian, EH25 9RG UK

**Keywords:** Avian Influenza, Phylogeography, General Linear Model

## Abstract

**Background:**

Avian influenza virus (AIV) causes both severe outbreaks and endemic disease among poultry and has caused sporadic human infections in Asia, furthermore the routes of transmission in avian species between geographic regions can be numerous and complex. Using nucleotide sequences from the internal protein coding segments of AIV, we performed a Bayesian phylogeographic study to uncover regional routes of transmission and factors predictive of the rate of viral diffusion within China.

**Results:**

We found that the Central area and Pan-Pearl River Delta were the two main sources of AIV diffusion, while the East Coast areas especially the Yangtze River delta, were the major targets of viral invasion. Next we investigated the extent to which economic, agricultural, environmental and climatic regional data was predictive of viral diffusion by fitting phylogeographic discrete trait models using generalised linear models.

**Conclusions:**

Our results highlighted that the economic-agricultural predictors, especially the poultry population density and the number of farm product markets, are the key determinants of spatial diffusion of AIV in China; high human density and freight transportation are also important predictors of high rates of viral transmission; Climate features (e.g. temperature) were correlated to the viral invasion in the destination to some degree; while little or no impacts were found from natural environment factors (such as surface water coverage). This study uncovers the risk factors and enhances our understanding of the spatial dynamics of AIV in bird populations.

**Electronic supplementary material:**

The online version of this article (doi:10.1186/s12862-016-0845-3) contains supplementary material, which is available to authorized users.

## Background

China plays a central role in maintaining a source population for influenza diversity globally - of the four human influenza pandemics between 1918 and 2009, the 1957 “Asian” H2N2 flu and H3N2 1968 “Hong Kong” flu were spread from China, and the origin of the viruses of these outbreaks were reassortants between human and avian strains [1]. More than half of the provinces and municipalities in China have experienced avian influenza outbreaks. The distribution and prevalence of the outbreaks vary among locations, and the major regional differences in AIV prevalence are thought to be influenced by ecological systems, husbandry practices, cultural behaviours and economic development [2].

Two types of locations are considered as potential hotspots of influenza outbreaks and therefore of particular importance; areas with high ecological complexity and those with high densities of domesticated birds and trade activity. Areas with a high ecological complexity include those which are rich in nature reserves, water resources, and on the flyway route of migratory birds. This creates an environment in which domestic ducks and wild aquatic birds live together, sharing water, food, and habitat. Three major Asian migratory bird flyways, the Central Asian, East Asian-Australasian and West Pacific flyways have major stop-over sites in China [3, 4]. Qinghai Lake in Northwest China is one of the most important breeding and stopover sites for migratory birds along the Central Asian Flyway [5]. In 2005 an outbreak of H5N1 in wild birds occurred in Qinghai Lake, and the viruses carried by wild bird migration from Qinghai Lake contributed to the global H5N1 prevalence and to the increase in human H5N1 influenza virus infections [6–10]. Studies of the HPAI H5N1 outbreaks at Qinghai suggest that the influenza viruses were circulated by wild birds through the Central Asian flyway, and are continuously undergoing evolution [11–13]. In addition, Poyang Lake in Jiangxi Province and Dongting Lake in Hunan Province located in South Central China are within the East Asian Flyway, and both possess important national natural reserves and wetlands. These locations have been identified with extensive AIV reassortment events and close interactions between wild migratory birds and domestic poultry, providing opportunities for outbreaks of highly pathogenic avian influenza (HPAI) virus [14–16].

Locations that have high economy activity and trade have also been considered as a potential epicentre for AIV [17]. HPAI H5N1 was first encountered in 1996 in Guangdong province in domestic geese. By 2000, the host range had extended to domestic ducks, which played a key role in the genesis of the 2003/04 outbreaks [2, 18, 19]. The outbreak of human infections with an emerging avian influenza A (H7N9) virus occurred in eastern China since late March 2013. Human infection of H7N9 appears to be associated with exposure to infected live poultry or contaminated environments, including markets where live poultry are sold [20, 21].

Previous studies on the transmission and distribution of avian influenza virus are mainly focused on the highly pathogenic outbreaks, such as H5 and H7 [2, 22]. However, phylogenetic studies have shown that the avian influenza with low pathogenicity in birds (e.g. AIV of H9 and H6 subtypes) could transmit their gene segments (especially internal segments) to other strains. The gene invasions contribute to the genesis and spread of new reassortant viruses, and with the potential to cause zoonotic outbreaks in human and mammals [23–28]. In addition, a wide range of inter-subtype reassortments have been found with respect to the internal segments of Eurasian AIV. Low pathogenic AIV especially in wild or domestic anseriformes are more likely to reassort with each other while highly pathogenic AIV in galliforms exhibits a lower reassortment rate [29]. Therefore, understanding the features of the total interaction pattern of avian influenza viruses with different subtypes, low and high pathogenicity, sampled from both wild and domestic birds are of particular importance.

The aim of the study is to describe the pattern of spatial diffusion AIV in bird populations in different regions of China and to indicate the related risk factors across the diversity of economy, agriculture, environment and climate. We explore the key sources and destinations of AIV diffusion in China by using viral sequence data to infer discrete trait phylogeographic models in a Bayesian framework. These models provide a quantification of the significant viral spatial transmission rates among regions in terms of agricultural, ecological and economic parameters. The results highlight the importance of the agro-economic impact on the evolution and spatial dissemination of the influenza virus which may inform future strategies for the surveillance and control of influenza.

## Methods

### Sequence data

A total of 835 full length avian influenza genomes isolated from China were downloaded from NCBI Flu Resources [30] (https://www.ncbi.nlm.nih.gov/genome/viruses/variation/flu/). For each internal segment, sequences were aligned using MUSCLE [31] in MEGA 5.1.0 [32]. Preliminary trees for each segment were generated by the neighbour joining method (with 500 bootstraps) using MEGA 5.1.0. Sequences that might contain incorrect date information were detected in these preliminary phylogenies using path-o-gen V1.2 (http://tree.bio.ed.ac.uk/software/tempest/) and were removed from the dataset. To account for potential sampling biases in space and time, sequences were subsampled to preserve diversity with respect to location (provinces), host, pathogenicity, subtype background and year of sampling using custom R scripts which resulted in 405 sequences. Additionally one clade, composed of H5N1 strains mainly isolated from the south central region (mainly including Guangdong, Guangxi, Hunan provinces) and monophyletic in all six segments was subsampled further to reduce over-representation but retain genetic diversity. The final dataset composed of all the internal gene segments of 320 AIV sequences covers an 18-year period from the 1996 to 2013 (accession numbers and details in Additional file 1: Table S9).

### Time-scaled phylogenetic analysis

Time-scaled trees were generated for each internal protein-coding segment with the 320 Chinese AIV dataset using BEAST version 1.7.3 [33]. Different substitution models, clock models and tree models (including constant population, exponential growth, Bayesian skyline plot and Bayesian skyride) were evaluated for segment 1 by estimating the marginal likelihoods of the models using the Harmonic Mean Estimator (HME) and Akaike’s Information Criterion for MCMC samples (AICM) in Tracer, and also by employing Path Sampling (PS) and Stepping Stone Sampling (SS) [34, 35] (Additional file 1: Table S1). A general-time-reversible (GTR) model with site to site rate variation in four categories from a Gamma distribution was chosen as the nucleotide substitution model. A constant population size model and a relaxed uncorrelated log-normal molecular clock model were chosen for all eight segments. The MCMC was run for 10^8^ steps and sampled every 10^4^ steps. Two independent runs were used in each segment to confirm the convergence and then combined (after discarding the first 10% as burn-in). Maximum Clade Credibility (MCC) trees for each segment were obtained from the tree posterior samples, and the mean ages of each ancestral node and the corresponding Highest Posterior Density (HPD) intervals were calculated using Tree Annotator.

### Spatial diffusion analysis

#### Geographic region classification

To investigate the spatial diffusion pattern of (AIV) in China, the locations of the 320 Chinese AIV genomes from a total of 27 provinces, were categorized into larger regions according to four different schemes: Traditional regions (TR), Economic regions (ER), Economic Divided zones (ED), and China Agro-Ecological Regions (CAR). Traditional regions are the 31 provincial-level divisions grouped into the six former administrative areas from 1949 to 1952: East, North, Northeast, Northwest, South Central and Southwest. Economic regions are categorized by the per capita income basis and composed of four areas: East Coast, Central, Northeast and Western. The ER scheme shows that provinces in the coastal regions of China tend to be more industrialized, while regions further inland are less developed [36]. Economic regions were further divided into seven smaller economic zones (ED) according to the result of the trait-phylogeny test described below. Specifically, the East Coast area (which contains the highest AIV prevalence) is divided into three smaller economic zones areas: Yangtze River Delta (YRD), Pan-Pearl River Delta (PRD) and Bohai Economic Rim (BER); The vast and less developed West areas are divided into Northwest and Southwest, resulting in 7 zones in total. We also considered using the China Agro-Ecological Regions scheme which contains 8 regions and groups neighboring provinces by poultry and duck density, distinguishing the plateau region from the south and north west [37]. The detailed classification and distribution of provinces into regions can be seen in Additional file 1: Table S2.

#### Distribution of region traits on tree phylogeny

Associations between phylogenies and location traits for each region type (TR, ER, ED or CAR) were tested using Bayesian Tip-association Significance testing (BaTS) [38]. Parsimony score (PS), association index (AI) and the monophyletic clade (‘MC’) size were calculated on a set of 1000 subsampled trees (for each segment), and 1000 state randomizations were generated to create the null distributions.

#### Discrete trait mapping of the spatial diffusion

The rates of viral migration between locations across different regions in China were inferred by using an asymmetric discrete traits model to reconstruct the ancestral subtype states of the internal nodes in time-scaled tree phylogeny of each segment. The significant transition rates between discrete traits were identified with the Bayesian stochastic search variable selection (BSSVS) extension of the discrete model. For the rates calculated from BSSVS, a BF test was adopted to identify significant non-zero transition rates and a cut-off of BF = 5 was applied [39]. Additionally we performed location state randomisation on the TR, ER, ED and CAR schemes in order to assess the effects of potential sampling bias on the ancestral state reconstructions [40].

### Predictors of spatial diffusion

#### Predictor data

The National Statistics Bureau and Ministry of Agriculture’s Animal Husbandry Bureau publish annual statistics for each province in mainland China in 27 out of total 31 different categories: including 21 provinces (excluding Hainan province which missing sequence data), two municipalities (excluding Chongqing and Tianjing), four autonomous regions, (excluding Inner Mongolia), including those relating to Agro-economics, the environment and climate. We chose some of these statistics as predictors to help inform a phylogeographical model of AIV diffusion in China. Data were referenced from the China statistical year book 2013 [36] and the China agriculture yearbook 2012 [41].

In the agro-economic categories, we focused on the standard statistics related to the production and sale of poultry productions (chickens, ducks and geese). The poultry density is the annual counts of each poultry type collected from farms and households at the township level and reported up through county, prefecture, and provincial administrative units with final submission to the national level. Given that live bird markets (LBMs) play a crucial role in the maintenance, amplification and dissemination of AIV [42, 43], we included the number of farm product markets per provinces as a potential predictor obtained from the national navigation map [20]. In addition, the agro-economic variables including human population density and the total capacity of transportation (by means of rail, highway and waterway) per province were also considered.

Physical environmental variables comprising the coverage of forest, surface water and natural resource were considered as potential predictors, since these may indicate a higher chance of wild birds interacting with poultry (which is expected to be positively correlated to disease outbreaks). Climatic factors including the average annual temperature and the average relative humidity were also considered. Finally, the distribution of host types within the AIV dataset were also considered; (1) the proportion of domestic birds vs wild birds; and (2) the proportion of domestic galliformes (mainly chickens) vs domestic anseriformes (e.g. ducks/geese) (Additional file 2: Figure S1).

Thirty three predictors were collected from 27 provinces (including autonomous regions and municipalities) in mainland China (Additional file 1: Table S10). Predictors having significantly high correlations (as measured using a pearson correlation test in R, correlation coefficient (r) > = 0.99) (e.g. poultry density versus output of Poultry meat) and measuring similar aspects (e.g., population at Year-end by Region versus population density) were grouped, and we kept one predictor per group, leaving 9 predictors. In addition, the number of AIV sequences per discrete location was considered as a separate predictor to test the impact of sampling effects on the diffusion process (Table 1). Predictor data at the provinces level was integrated into regions according to the traditional and economic region schemes (Additional file 1: Table S2). The final 10 predictors for each region type are summarized in Table 2.

**Table 1:** Predictors for traditional regions (TR) and economic divided regions (ED)

TR	East	North	Northeast	Northwest	South Central	Southwest	
Poultry dens	14132.8	3766.9	3793.7	539.1	10115.2	1514.8	
Farm market	29912	5601	8604	5823	17588	7067	
Forest Volume	128885.7	17056.33	256744.1	87692.69	145843.83	563511.33	
Nature reserve	4.28	6.34	13.22	15.06	5.66	16.33	
Temperature	17	12.49	5.76	8.83	18.5	13.86	
Humidity	69.83	52.28	66.22	55.85	74.47	65.75	
Water resource	6222.31	201.61	1575.44	2366.17	7041.57	9751.36	
Human dens	6688.97	1790.22	529.06	357.86	1974.9	485.01	
Transportation	1363443.1	389900.12	326827.45	295949.22	1003544.8	296865.77	
Sample size	119	14	16	16	134	21	
ED	BER	Central	Northeast	Northwest	PRD	Southwest	YRD
Poultry dens	7176.94	9959.04	3793.74	539.12	2751.7	2796.32	6845.58
Farm market	6638	12094	8604	5823	10653	8808	21975
Forest Volume	15751	129714	256744	87693	78620	610387	20826
Nature reserve	5.43	5.73	39.67	15.06	4.92	14.26	3.62
Temperature	13.72	15.76	17.28	8.83	20.92	15.37	16.65
Humidity	53.61	68.72	66.22	55.85	78.38	68.55	69.44
Water resource	317.88	5800.06	1575.44	2366.17	3527.56	11837.72	1733.64
Human dens	2231.64	2061.8	529.06	357.86	897.94	683.14	5064.57
Transportation	578895	1170352	326827	295949	340422	458222	505863
Sample size	30	98	16	16	65	49	46

The data of 10 selected predictors per area for the 2 types of regions

The unit of each predictors are listed below:

1 Poultry density (10000unit/km^2^)

2 Number of farm product markets (region) (unit)

3 Stock Volume of Forest (10000 cu.m)

4 Percentage of Nature Reserves (%)

5 Average Temperature of Major Cities (°C)

6 Average Relative Humidity (%)

7 Surface Water Resources (100 million cu.m)

8 population density (10000 persons/km^2^)

9 Freight by transportation total (10000 tons)

10 Sample size (unit)

**Table 2:** Correlation test among predictors

TR	1	2	3	4	5	6	7	8	9	10
1 Poultry dens		0.96	−0.3	−0.84	0.7	0.66	**0.28**	0.89	0.98	0.91
2 Farm market			**−0.18**	−0.69	0.65	0.67	0.39	0.93	0.97	0.87
3 Forest Volume				0.62	**−0.02**	0.35	0.7	−0.32	**−0.28**	**−0.21**
4 Nature reserve					−0.65	**−0.26**	**0.08**	−0.76	−0.8	−0.73
5 Temperature						0.52	0.65	0.6	0.76	0.82
6 Humidity							0.69	0.36	0.65	0.77
7 Water resource								**0.2**	0.37	0.48
8 Human dens									0.9	0.7
9 Transportation										0.94
10 Sample size										
ED	1	2	3	4	5	6	7	8	9	10
1 Poultry dens		0.45	−0.319	−0.38	0.21	**0.03**	−0.12	0.63	0.86	0.6
2 Farm market			−0.25	−0.37	0.41	0.49	**−0.08**	0.87	0.22	0.36
3 Forest Volume				0.39	0.05	0.2	0.86	−0.46	−0.11	**−0.005**
4 Nature reserve					**−0.03**	−0.11	−0.06	−0.53	−0.4	−0.57
5 Temperature						0.86	**0.09**	0.15	**0.04**	0.45
6 Humidity							0.36	0.08	**0.05**	0.59
7 Water resource								−0.29	0.2	0.42
8 Human dens									0.32	0.23
9 Transportation										0.79
10 Sample size										

The values in the cell represent the correlation coefficient of the two predictors in the column and row, >0.5 indicates a supported positive correlation and < −0.5 indicates a supported negative correlation. The predictors which have weak correlation with others are put into the same GLM analysis, with the correlation coefficient values marked in bold

#### Hypothesis test of predictors

To test the contribution of potential predictors for the CTMC transition rate matrix between locations, an extension of the phylogenetic diffusion model was used to parameterize these rates as a log-linear function of a set of predictor matrices within a generalised linear model (GLM) [44, 45]. The GLM specifies coefficients for each predictor, allowing the estimation of their contribution to the diffusion process and indicator variables to model the inclusion or exclusion of each predictor.

In this study we created matrices for each predictor in regional categories (Table 1), and these were transformed to log space and normalised prior to their incorporation. Symmetric (no directions) and asymmetric matrices (with donor/recipient directions matrices) were used. Firstly, we performed several individual analyses, using pairs of independent (or near independent) predictors as shown in Table 2 to determine the importance of one predictor relative to the other. The independent predictor pairs and their impact on the spatial transmission were analysed in GLM models.

Secondly we also performed a single GLM-diffusion analysis including all predictors (in total/donor/recipient directions) as well as sample size as a predictor, and one in which all the predictors except sample sizes were used to examine the possible sample size effect. In analyses with many predictors, a binomial prior that prefers the inclusion of a small number of predictors was used together with a multivariate normal updater for the coefficients which considers their correlation as described in Faria et al. [45].

## Results

### Phylogeographic evolution of AIV in China

In this study the data set consisted of 320 AIV genomes which were obtained from 27 out of the 32 provinces in China. Of these, Guangdong, Fujian, Hunan, Jiangsu and Jiangxi provinces had the highest number of sequences (Additional file 2: Figure S1). The majority of AIV in the dataset was sampled from domestic birds, with 47.2% from domestic anseriformes (ducks and geese), 32.8% from domestic galliformes (chickens), and 19.4% from wild birds (Additional file 2: Figure S1). Subtypes H5, H6, H7 and H9 are prevalent in China and are circulating in domestic birds, and our dataset includes 31.9% H5N1 and 19.1% H9N2.

Bayesian phylogenetic trees of the 6 internal segments of the 320 Chinese AIV were generated (Additional file 2: Figures S4–S7 and Additional file 3: Figure S9 A–F). The evolutionary rates for five segments (PB2, PB1, PA, NP and M) were estimated and range from 2.47 × 10^−03^ to 4.36 × 10^−03^ substitutions per site per year. The estimated time to most recent common ancestors (TMRCA) of these five internal segments range from 1955 to 1960. However, the NS segment has a lower substitution rate (1.66 × 10^−03^) and earlier TMRCA (1554, HPD: 1175–1836) due to the divergent alleles A and B [46], the detailed evolutionary rate and TMRCA estimations (mean and 95%HPD intervals) for each AIV segment can be found in Additional file 1: Table S4.

The strength of phylogeny-trait association was further tested on different regions types upon the Bayesian phylogenetic trees of the PB2 segment. The results are summarized in Table 3, and statistical analysis of AI and PS of the full phylogeny revealed that for TR, ER, and ED, the total number of migration events occurring in each category was less than expected under the null hypothesis (*P* <0.005) indicating that there is a sufficient correlation between these geographical characters and the phylogeny. However, the AI and PS statistics under the CAR scheme were not significant. The significance of the clustering of areas within each region type was further indicated by the estimation of the maximum monophyletic clade (MC) size. There is evidence of clustering of AIV belonging to each of the six areas in the traditional region; Northeast and Central areas which belong to the economic region also have a strong clustering support. However, the East Coast area is the most dominant group (141/320 sequences) among economic regions with a significantly higher value (mean MC = 13.95) than the other areas (mean MC = 3 to 6). This shows a very strong phylogeny-trait association thus indicating strong gene flow within the areas rather than to the other areas. To further identify the pattern of gene flow within east coast region, we further divided it into three economic zones: Pan-Pearl River Delta (PRD), Yangtze River Delta (YRD) and Bohai Economic Rim (BER). The three smaller economic zones showed evidence of trait-phylogeny correlation with the MC value from 2 to 4, with strong supported. In addition, the Western area showed poor statistical support, indicating the AIV are randomly distributed across the tree phylogeny rather than clustering together in the same clade. This can be explained by the limited number of sequences (65/320) sampled from ten provinces divided into a Northwest and a Southwest area with supported clustering for both with mean MC = 2.

**Table 3:** Results of trait-phylogeny association of different region types on PB2

Region^a^	Statistics^b^		Observed	Null	*P*-value
			mean^c^	mean	
TR	AI		14.10 (13.13, 15.17)	22.56 (20.68, 24.42)	<0.005
	PS		114.70 (112.00, 118.00)	149.31 (142.70, 156.17)	<0.005
	MC (6)	East	8.89 (8.00, 11.00)	3.59 (2.63, 5.11)	0.002
		North	2.00 (2.00, 2.00)	1.16 (1.00, 2.00)	0.05
		Northeast	1.99 (1.90, 2.00)	1.21 (1.00, 2.00)	0.06
		Northwest	1.98 (1.9, 2)	1.19 (1.00, 2.00)	0.06
		SouthCentral	12.50 (12.00, 13.00)	4.00 (3.00, 6.00)	0.002
		Southwest	3 (2.90, 3.00)	1.34 (1.00, 2.00)	0.006
ER	AI		12.67 (12.10, 13.17)	27.42 (24, 29.20)	<0.005
	PS		103.40 (103.00, 104.10)	148.41 (148.00, 149.40)	<0.005
	MC (4)	East Coast	13.95 (13.9, 14)	4.11 (3.01, 6.00)	0.001
		Central	6 (5.90, 6.00)	3.1 (2.07, 4.17)	0.008
		Northeast	4.27 (3.00, 7.00)	1.97 (1.25, 3.00)	0.001
		Western	3.00 (3.00, 3.00)	2.37 (1.95, 3.18)	0.14
ED	AI		15.54 (14.46, 16.68)	27.42 (25.67, 29.00)	<0.005
	PS		137.29 (134, 140)	192.83 (185.83, 199. 37)	<0.005
	MC (7)	BER	2.00 (2.00, 2.00)	1.12 (1.00, 1.99)	0.03
		PRD	4.27 (3.00, 7.00)	1.97 (1.25, 3.00)	0.001
		YRD	4.05 (3.90, 4.00)	2.00 (1.25, 3.00)	0.004
		Central	6.00 (6.00, 6.00)	3.1 (2.07, 4. 17)	0.008
		Northeast	1.98 (1.90, 2.00)	1.2 (1.00, 2.00)	0.06
		Northwest	1.98 (1.90, 2.00)	1.19 (1.00, 2.00)	0.06
		Southwest	3 (2.9.00, 3.00)	2.9 (2.00, 3.00)	0.06
CAR	AI		25.70 (24.59, 26.93)	27.12 (25.52, 28, 41)	0.09
	PS		185.82 (183.00, 189.00)	189.72 (183.60, 196.47)	0.22
	MC (8)	East	3.00 (3.00, 3.00)	2.14 (1.39, 3)	0.09
		South	3.00 (3.00, 3.00)	2.99 (2.11, 4.00)	0.5
		Central	2.52 (2.00, 3.00)	2.79 (2.00, 4.00)	1
		North	1.46 (1.00, 2.00)	1.86 (1.13, 3.00)	1
		NorthEast	2.00 (2.00, 2.00)	1.19 (1.00, 1.98)	0.04
		SouthWest	1.43 (1.00, 2.00)	1.15 (1.00, 2.00)	1
		NorthWest	1.88 (1.00, 2.00)	1.08 (1.00, 1.81)	0.02
		Plateau	1.85 (1.00, 2.00)	1.13 (1.00, 2.00)	0.04

^a^region type classification; ^b^the three statistics: Association Index (AI), Parsimony Score (PS) and Maximum monophyletic clade (MC) size of the area traits per region type;

^c^the last 3 columns correspond to posterior estimates of observed and expected values and the *p* value for the AI, PS and MC metrics. Lower AI and PS values represent strong phylogeny–trait association; while larger values of mean MC indicate increased phylogeny-trait association and the significance indicated by *p*-value (<0.05)

We also compared the trait-phylogeny interaction among the six internal segments using the economic regions as traits (Additional file 1: Table S3). In the full phylogenies, AI and PS varied among six internal segments, ranging from PB2 (AI mean = 11.6; PS = 96.9) to NS (mean = 17.7, PS = 117.6), indicating an increase in gene flow from PB2 and NS. Similar results are found in the MC size of the East coast area, which is the area with the majority of AIV, the MC size of PB2 (mean = 20) is larger than the other five segments, while NS has the lowest value (mean = 7.7), which indicated it is more randomly distributed, which could be attributed to the Allele A and B divergence of NS segment [47].

### Quantified spatial diffusion patterns

**Fig. 1:**
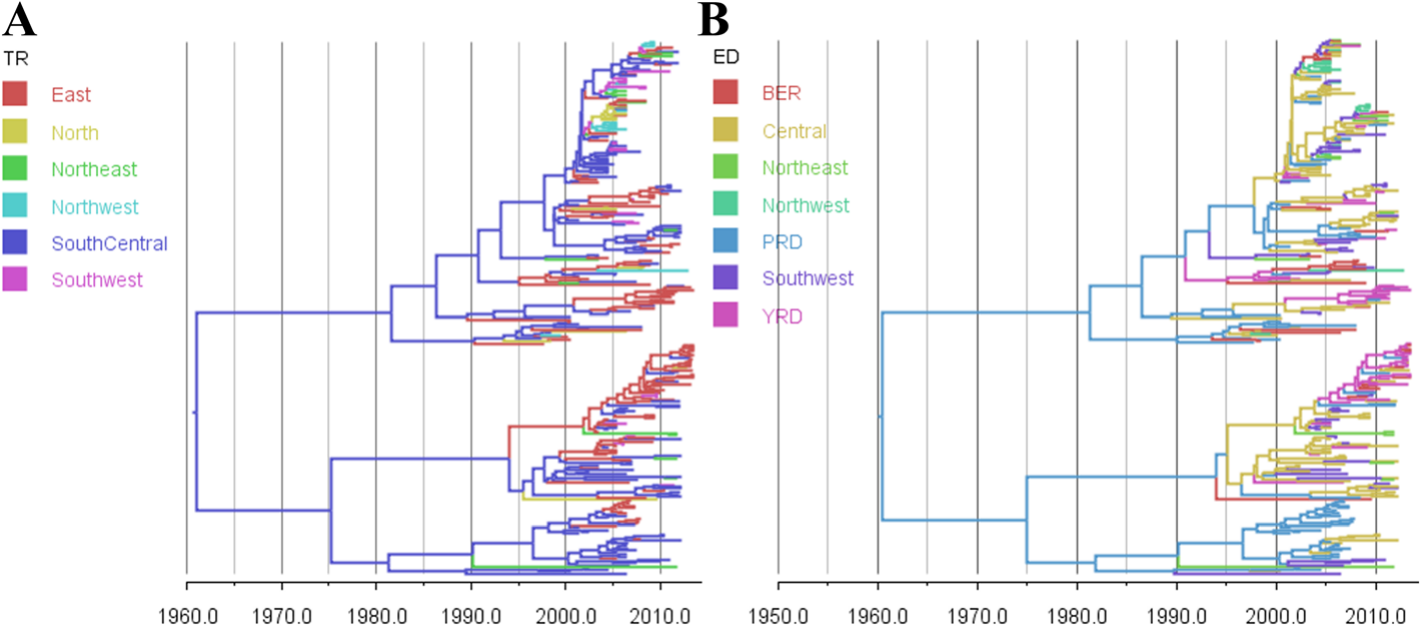
Bayesian MCC phylogenies and between-regions diffusion networks on PB2 gene segment of AIV in China. The sequences are classified according to their variant designation in (1) TR: Traditional geographic regions; (2) ED: Economic divided zones. Branches are coloured according to their descendent nodes annotated by the different sampled areas within the region type, with the key for colours shown on the left. **a** Tree phylogeny with branches coloured according to the Tradition regions. **b** Tree phylogeny with branches coloured according to the Economic divided zones

To explore the spatial diffusion patterns of AIV across different regions in China, we used a discrete trait procedure on the empirical trees sampled from the posterior distribution of original phylogenies of the six internal segments. Patterns of AIV spatial diffusion in China were quantified under a BSSVS procedure. In each internal segment phylogeny, tree branches are coloured to the predicted states of the descendent node, according to geographic distance (TR) or the economic relationship (ED) (Fig. 1 and Additional file 2: Figures S3–S7). Co-circulation of multiple lineages and diverse gene flows of avian influenza virus between different areas of China were detected. For the TR areas, we found that South Central is the source of the majority of lineages (Fig. 1a). Multiple introductions are observed among the economic divided region type, with the Pan-Pearl River Delta (including Guangdong and Fujian provinces) as the main ancestor of all AIV, and the Central area represented as the source of two main clades (Fig. 1b and Additional file 2: Figures S3–S7).

**Fig. 2:**
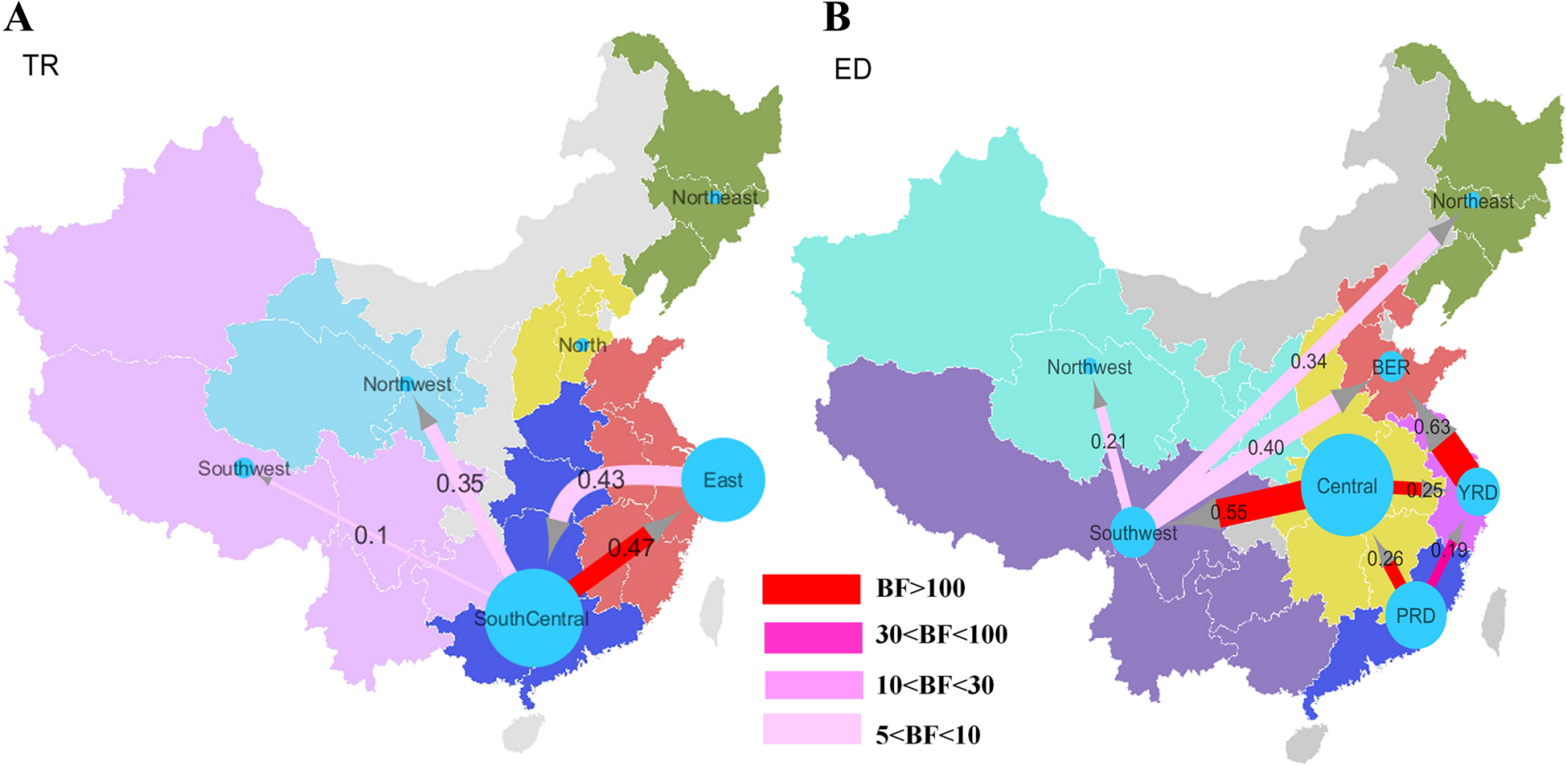
Diffusion networks with quantified diffusion rate and BF support. Quantified diffusion rate between regions and BF support were estimated by an irreversible discrete trait model on the phylogeny of PB2 segment. Areas for each region type are labelled by the same colour in the region annotated phylogenetic trees in Fig. 1. The size of the blue circles represent the AIV sample size in each area. The diffusion rate and statistical support for all six internal segments are summarized in Additional file 1: Table S6 and Table S7. The map source is National Science & Technology Infrastructure of China, National Earth System Science Data Sharing Infrastructure (http://www.geodata.cn). Traditional regions (**a**) and economic divided zones (**b**) were mapped in ARCGIS (http://www.esri.com/software/arcgis). The diffusion networks on the maps were made via Cytoscape v3.1.0 (http://www.cytoscape.org/). **a** Diffusion network of AIV among traditional regions. **b** Diffusion network of AIV among economic divided zones

The transmission networks among traditional regions (TR) as well as economic zones (ED) for PB2 segment are shown on the maps in Fig. 2 (A:TR, B:ED). The detailed diffusion rate and BF support for each region and each internal segment are summarized in Additional file 1: Table S6 (TR) and Additional file 1: Table S7 (ED). In general, the BSSVS procedure identified supported transition rates between locations for all segments (with BF > 5) in both region types (2 to 3 pairs/segment in TR; 4 to 9 pairs/segment in ED). However not all the strongly supported BF rates (BF > 100) actually correspond to a high magnitude transition rate. The average diffusion rate (exchange/year) of six internal segments also vary (For TR: PB2 = 0.31; PB1 = 0.3; PA = 0.46; NP = 0.24; M = 1.18 and NS = 0.91; For ED: PB2 = 0.35; PB1 = 0.42; PA = 0.5; NP = 0.42; M = 0.63 and NS = 0.70).

Among the 15 possible transition rates between locations for the traditional regions (TR), three are found with significant BF support in PB2, PA and NP segments, while two are found in the other segments. The South Central area (composed of Guangdong, Guangxi, Henan, Hubei, Hunan) was found to play a role as a major source region transmitting AIV to the East (composed of Shandong, Jiangsu, Anhui, Jiangxi, Zhejiang, Fujian, Shanghai), Northwest (Gansu, Ningxia, Qinghai, Xinjiang, Shaanxi) and Southwest areas (Guizhou, Sichuan, Tibet, Yunnan) (Fig. 2a). This also can be seen in the region-annotated MCC tree which shows that the majority of the ancestral nodes are inferred to have a South Central location (Fig. 1a). The most significant gene flow is from South Central to East area (mean rate = 0.47, BF = 3703). The Northwest area is also likely to accept AIV virus from other areas. Invasions were also found from Northeast or from South Central to other areas, but only with moderate support (3 < BF < 6) (Additional file 1: Table S6). The only bidirectional transmissions are found between South Central and East in PB2 segment.

Using the economic zones classification of locations (ED), two major sources of AIV migration are found (Fig. 2b and Additional file 1: Table S7). One is the Central area (composed of Shanxi, Anhui, Henan, Hubei, Hunan, Jiangxi), which has highly supported virus transmissions (BF > 100) into the southwest area and YRD (Shanghai, Jiangsu, Zhejiang). The other source is PRD (Guangdong and Fujian) with high supported transmissions to YRD (mean = 0.19, BF = 43) and Central areas (mean = 0.26, BF = 158). In addition, the Guangxi province might also play an important role as a source location when it is included in the southwest area in ED type. There are wild bird migration routes from southwest to Northeast, Northwest and BER, however these routes have weak support (BF < 10) (Fig. 2b). In comparison, AIV are mainly transmitted to two economic zones: YRD and BER (Beijing, Hebei and Shandong). Specifically, YRD mainly receives AIV from Central (mean = 0.19 to 0.28, BF > 100) and moderate supported invasion from PRD (mean = 0.18 to 0.21, BF = 10 to 43). A high transmission rate is identified from YRD to BER (mean rate = 0.62 to 0.97), which is consistent in all six segments with high support (BF = 573 to 5172).

Results from discrete traits analyses can be influenced by severely under or over-represented numbers of sequences with respect to location states. To mitigate this potential bias, we selected the samples from those publically available, applying a random subsampling procedure to attempt to preserve diversity in province location, host, subtype and year whilst reducing over-representation in South Central area in this study. However, given that the sample size is correlated with poultry density and also reflective of the actual prevalence of AIV (Table 2), we did not down-sample AIV sequences to even number between different regions in China, because that would severely reduce the subtype and host diversity within those regions with the highest AIV prevalence. Therefore, we also investigated the effect of sampling heterogeneity on ancestral state reconstruction by performing sensitivity analyses using location state randomization (Additional file 2: Figure S3) [40]. In ED, sampling frequencies of location states have little impact on the root location probability (and therefore are unlikely to be influencing the reconstructions as a whole [40]; however, in TR, we found for the original (un-permuted) and the permutated states, the South Central region had the highest root state frequency.. Therefore, the state reconstruction of the Traditional region classification scheme may still influenced by the large number of samples from the south central region, but this is an area with dense avian populations so the inclusion of a higher number of samples from this region is appropriate

### Hypothesis-based spatial analysis

Ten potential predictors over categories including economic (agricultural) activity, environment, climate as well as the sample size of AIV (Table 1) were selected to identify the possible factors that determine the viral diffusion of avian influenza among different regions of AIV in China, by employing a generalized linear model which is an extension of a Bayesian phylogenetic diffusion model on six internal genes.

In this study, individual analyses using pairs of uncorrelated predictors were performed, and a single GLM-diffusion analysis including all ten predictors was also conducted. In the single GLM analysis, only sample size was strongly correlated with viral spatial diffusion, the other predictors showed no sign of correlation with diffusion. We also performed an analysis with all predictors but excluding sample sizes, which gave weaker but still significant coefficients and BF supports compared to those estimated using separate analysis, indicating the inclusion/exclusion of sample size with other predictors in the same GLM analysis can have a strong impact on the results (data not shown). However, several predictors are correlated with sample size (e.g. poultry density and transportation freight), which is unsurprising since the sampling is expected to be approximately reflective of prevalence. Therefore, individual analyses using pairs of uncorrelated predictors were preferred and the results were described below.

**Fig. 3:**
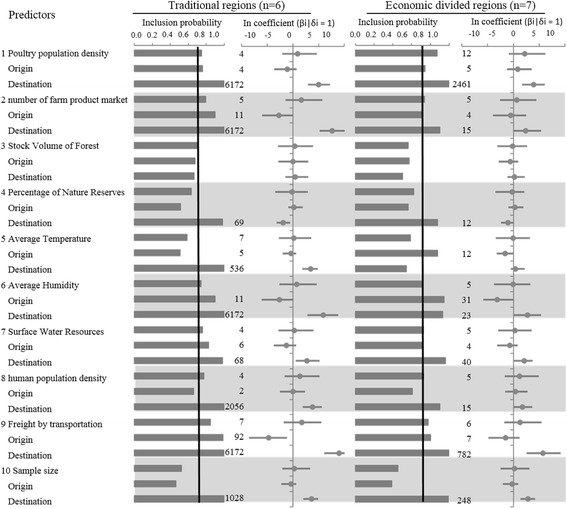
Predictors of Chinese AIV spatial diffusion. The inclusion probabilities (IP) shown in the bar plots are defined by the indicator expectations which reflect the frequency at which the predictor is included in the model and therefore represent the support for the predictor; The predictors with Bayes factor (BF) are indicated with the corresponding BF value (those with BF < 5 are not shown); The contribution of each predictor, when included in the model is represented by the conditional effect sizes (cES) and 95% HPD on a log scale (ßi|δi = 1). The zero line not included in the credible intervals indicates the positive correlation (on the right, above 0) or the negative correlation (on the left, below 0)

**Fig. 4:**
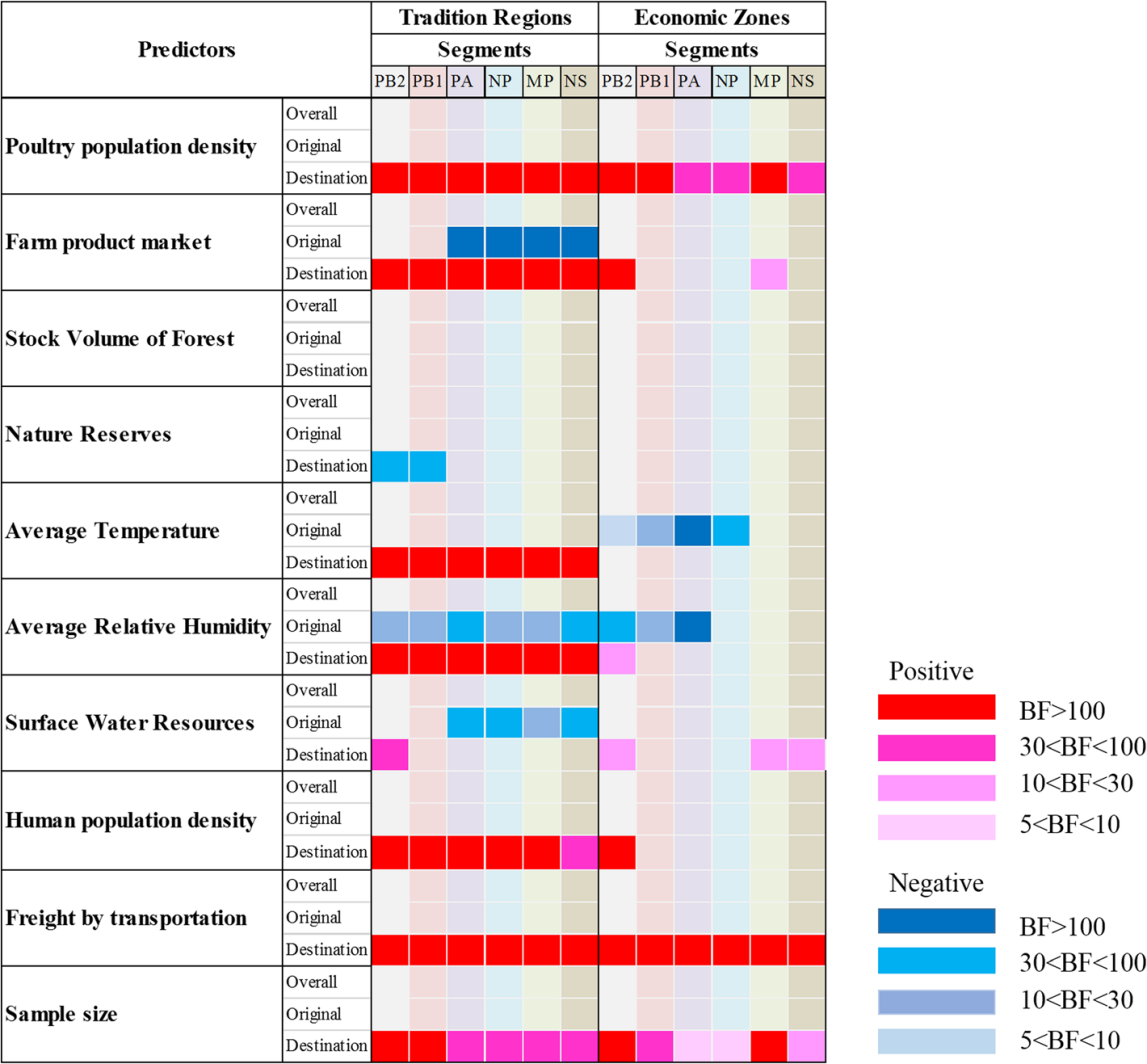
Predictors of the Chinese AIV spatial diffusion on 6 internal genes segments. For the 10 predictors, their correlations to the AIV diffusion in two region types (traditional regions and economic divided zones) are summarized in the cells, with rows representing predictors and columns representing six internal segments (PB2, PB1, PA, NP, M, NS) of the Chinese AIV dataset. The markers of the correlation with statistical supports are on the right. Red indicates positive correlation, blue indicates negative correlation. The rank of colours indicating the Bayes factor (BF) support from low to high; blank cells indicate that either no correlation or no BF support was detected. The inclusion probabilities and the log coefficients for each predictor for each region type in all six internal segments are in Additional file 1: Table S8

The identified determinants of spatial AIV (using PB2 gene segment as an example) diffusion are summarized in Fig. 3, which shows the mean and 95% HPD of the BF support for each predictor as well as the conditional effect sizes (coefficient) on a log scale. The conditional effect sizes are the estimated effect sizes when the indicators =1, and they summarize the coefficients conditional on the predictor being included in the GLM model. The results of all six internal segments and both region types are summarized in Fig. 4 and Additional file 1: Table S8.

In general, the potential predictors have strongly directional impacts on the AIV spatial diffusion, which means that the predictors have significant impacts on the sources and/or the recipients rather than the overall transmission. The impacts are not always consistent among the six internal segments, which can be reflected in both the effective sample size and the BF support, more so for the economic zones (ED) than for the traditional regions (TR), but similar trends (positive or negative) are found for predictors of transmission between traditional regions across segments, with varied statistical support (Fig. 4).

Overall we found the agro-economic predictors played decisive roles in viral spread in both traditional regions and economic zones in China. Specifically, poultry population density and productivity have a significant positive correlation, consistent in all six segments with high estimated size of the effect (mean = 3.5 to 5). The statistical support for its inclusion in the model (BF = 256 to 6172) indicated that the locations with high poultry density and productivity are more likely to accept (or “absorb”) the viral migration. The GLM diffusion model also revealed that the number of farm product markets (selling poultry product and also live poultry) is also a driver of the AIV dissemination in China. It has a negative correlation (mean = −4.9 to −5, BF = 166 to 293) in the source (in PA, NP, M and NS segments) and positive impact in the destination regions (mean = 5.9 to 7.6, BF = 6172) in all six segments, indicating the direction of high viral transmission rate is towards the location with high demands of poultry products. The productivity and sale of poultry is also linked with the impact of human actions, and Human population density in the destination location has support (mean = 2.8 to 3.8, BF = 38 to 2056) in the models for different segments. Interestingly, the capacity of freight transportation has the most significant impact on the viral diffusion (mean = 7.7 to 8.9, BF = 6172), indicating the movement of avian influenza virus is mainly dependent on the human transportation, not through wild bird migration. In addition, climate factors (temperature and relative humidity) are have impact on the inferred diffusion pattern; locations having comparably higher temperature (mean = 2.9 to 3.4, BF = 122 to 6172) and higher humidity (mean = 4.2 to 5.9, BF = 122 to 6172) are more likely to accept the virus than those with lower values.

The environmental factors (coverage of forest, water and natural reserves) impact on the viral diffusion to some degree. Surface water resources has a negative impact on the source regions in PA, NP, M and NS segments, suggested that AIV are not likely to be transmitted from region that is rich in water resources; and the coverage of natural reserves has a slight negative correlation with the recipient location of the viral diffusion in only PB2 and PB1 segments (mean = −1.95 and −1.85, BF = 56 and 69), while forest coverage does not yield noticeable support in the models for any of the segments.

The possible impacts of the distribution of host population based on the AIV dataset (the proportion of domestic birds and the proportion of domestic galliformes) are also considered as possible explanatory variables in the GLM model in this study, but neither of these have any statistical supported for impact on viral diffusion (Additional file 1: Table S8).

## Discussion

By applying phylogeographical diffusion models, the key source/sink locations were captured and the rates of movement were simultaneously informed from the evolutionary history of the viral segments encoding for internal proteins. The results highlighted that AIV in China were mainly exported from the Yangtze River Delta (YRD: including Guangdong, Fujian provinces) and the South Central area (including Hunan, Hubei, Jiangxi provinces). Previously, Martin et al. have postulated YRD as a hypothetical influenza epicentre [2], while considering that the multiple viral exposures from South Central areas are probably due to its location along the Central Asian Flyway [21]. In addition, H7 viruses with multiple NA subtypes were found in ducks in Jiangxi province (in South Central), suggesting an epidemiological bridge from migratory birds to sentinel farm ducks and then to market birds. Epidemiological studies also suggested that HPAI H5N1 outbreaks in poultry mostly occurred in areas which overlapped with habitats for wild birds, whereas outbreaks in wild birds were mainly found in areas where food and shelter are available [48]. Bidirectional movement of wild birds among the provinces along the flyway route in south central areas were identified in studies tracking the movements of migrating wild birds, and the transmission of HPAI H5N1 to Qinghai lake (Northwest) have also been identified [15, 49, 50]. The HA and NA genes of the recent human H7N9 cases (2013–2015) may originate from duck avian influenza viruses, which might have obtained the viral genes from wild ducks along the east Asian flyway a year before [51].

In contrast, the East Coast areas especially YRD are major targets for receiving the viral strains. The importance of the east coastal region as the hotspot of avian influenza outbreaks is supported by previous studies: The potential hotspots for H5N1 reassortment being identified in previous studies are the coastal provinces [52]; HPAI H5N1 risk distributions are identified in both south and north of the YRD [2]. In addition, the recent H7N9 outbreaks occurred initially in the YRD, and then spread along both south and north of the East Coast lines [20].

The transmission between Southwest and Northwest is supported by the studies that identified the outbreaks reported in poultry or wild birds on the Qinghai-Tibet Plateau. The initial outbreaks occurred in poultry then occurred in wild birds afterwards. The locations of outbreaks started in Lasa (in Xinjiang province) then were transmitted to Tibet [37]. No significant viral exposure has been identified from the Northeast China. Northeast China has a rapid development of intensive chicken production with intensive poultry production systems. It has invested in disease prevention measures and applies mass vaccination of their flocks in order to prevent viral infection and spread [2].

Most of China's economic production and growth originate in the coastal regions. The PRD and YRD have served as the commercial arteries for China’s economic development and major manufacturing and transportation hubs since late 1970s; BER is rising as a vital economic region in North in recent years. Cities in economic zones have built a complete network for water, land and air transportation, and are interconnected by highways and railways [53]. Therefore, the strong transport connections among the three economic zones can explain the strong viral flow linkages being found in our study, especially between PRD and YRD, which showed a trend of bidirectional transmission. In addition, the connection can also be the explanation of the extremely strong positive correlation between the capability of transportation and the gene flow that identified in the GLM results.

We found poultry density is strongly correlated with viral diffusion in the AIV sink regions. More than 75% of the world's domestic duck population is bred in China and more than 13 billion chickens in China, of which 60% are raised on small farms [8]. Shandong and Hebei (within BER) are the major sources of the output of poultry meat (mainly chickens) and have the highest poultry density, whereas the PRD has much less poultry being raised in the commercial farms with more being raised as free range and backyard [54]. Similarly, regions with higher number of live bird markets are more likely to accept the AIV gene flow. Most of the live poultry markets in China and Southeast Asia have been characterized by poor sanitation where the domestic ducks and geese are being housed together with chickens [16]. Our results supported that AIV epidemiological processes are more likely to occur in highly-populated areas, and there are higher possibilities of AIV transmission through trade and farming-related activities [2].

Similarly, regions with higher number of live bird markets are more likely to accept the AIV gene flow. Most of the live poultry markets in China and Southeast Asia have been characterized by poor sanitation where the domestic ducks and geese are being housed together with chickens [16]. Surveillance and phylogenetic studies showed that the current human H7N9 outbreak virus has spread over a large geographic region and is prevalent in poultry (mainly chickens) and in live poultry markets in the outbreak areas, which are thought to be the immediate source of human infections [21]. Our results also supported that AIV epidemiological processes are more likely to occur in highly-populated areas, and there are higher likelihood of outbreak detections and higher possibilities of HPAIV H5N1 transmission through trade and farming-related activities [2].

The climate and natural factors have a mild impact on the viral spatial diffusion when considering analysis by geographic regions, but little or no impact on AIV diffusion among the economic regions. Firstly, AIV are highly likely to be transmitted to areas with higher temperature and humidity, which also can be explained by the warmer regions as a rest place for migrating wild birds. The transmission of AIV is mainly by faeces and contact between bird populations (sometimes by faecal-oral route and fomite transmissions), and the pathway also differs between wild birds and terrestrial birds, thus this is not expected to be influenced by the same factors as airborne transmission in humans [55]. In addition, AIV transmission are shown to have impact from the amount surface water resources and the coverage of natural reserves. This is consistent with the suggestion that AIV are likely to be transmitted into areas rich in water resources and natural reserves such as wetlands and lakes that are important stopover, breeding, or wintering sites for migratory water birds [56, 57].

It is crucial to recognize that if variables in the GLM are correlated, the results can be misleading. A previous similar study also showed that the sample size absorbed effect of other predictors in a GLM model [44]. Therefore, pairs of uncorrelated predictors were used in separate analyses in this study, and a single GLM-diffusion analysis including all ten predictors was also conducted for comparison. The results showed no predictor expects sample size has correlation with viral spatial diffusion. In addition, we did analysis with all predictors together but excluding sample sizes, which gave weaker but still significant coefficients and BF support compared to those estimated by using separate analysis, indicating the inclusion/exclusion of sample size with other predictors in the same GLM analysis impacts the results. However, certain predictors (e.g., poultry density) are correlated with sample size in this study, so the results of GLM analysis are expected to be affected when sample size is included together with other correlated predictors. Finally since the agricultural and economics statistics are updated each year, it would be interesting to take into consideration the change of the agricultural and economic factors through the same years as the phylogenetic time scales to explain the AIV evolution for future studies.

## Conclusions

In summary, this study describes the AIV diffusion pattern among geographic regions and economic zones in China by looking at the evolution of internal segments of AIV sequences. We found that transmission patterns of avian influenza virus in China are mainly driven by exposure from the South region and the central region, which are composed of the areas along the East Asian migration routes also with high density of poultry industry; while virus tends to be imported into the east coast areas that are economically developed. We highlight that the transmission between regions of AIV in China is largely driven by human activity including transportation of the poultry product and trade rather than natural and ecological conditions. The impact of economic development on the transportation of domestic animals and the agricultural practices may enhance spread and evolution of influenza in certain areas, and good biosecurity measures as adopted in the North East region may limit viral transmission. Also, agricultural policy should be coherent with public health policy with the aim to reduce the risk of emergence of cross-host transmission from animals to humans.

## Additional files

Additional file 1: Table S1. Tree model fit comparison. **Table S2.** Classification of provinces into areas in four region types. **Table S3.** Results of trait-phylogeny association of different segments. **Table S4.** Estimation of evolution parameters of different segments. **Table S5.** Host type and Subtype correlation with Sample Size. **Table S6.** Transmission rate and statistical support between areas from Traditional Regions. **Table S7.** Transmission rate and statistical support between areas from Economic Divided Zones. **Table S8.** Coefficients of predictors of spatial diffusion of Chinese AIV from GLM analysis. **Table S9.** Sequence information and Accession number of 6 internal segments of AIV in this study. **Table S10.** Original 31 predictor data per province. (PDF 2305 kb)
Additional file 2: Figure S1. Host distribution of 320 Chinese AIV sequences in Traditional Region, Economic Region, Economic Divided zone, and China Agro-Ecological Region types. **Figure S2.** Distributions of 320 Chinese AIV sequences in the sampled time, host order, subtype and sampled provinces. **Figure S3.** Influence of the sampling scheme on the phylogeographic reconstruction. **Figure S4.** PB1 tree mapping with region traits. **Figure S5.** PA tree mapping with region traits. **Figure S6.** NP tree mapping with region traits. **Figure S7.** M tree mapping with region traits. **Figure S8.** NS tree mapping with region traits. (PDF 864 kb)
Additional file 3: Figure S9. (A to F) Bayesian MCC phylogenies of 6 internal segments of 320 Chinese AIV sequences labelled with sequence names on tips and Bayesian posterior probability on nodes. (A) PB2; (B) PB1; (C) PA; (D) NP; (E) M; (F) NS. (ZIP 523 kb)
