# Supplementary material for: Quantifying predictors for the spatial diffusion of avian influenza virus in China
Source: BMC Evol Biol. 2017 Jan 13;17:16. doi: 10.1186/s12862-016-0845-3 (PMC5237338; doi:10.1186/s12862-016-0845-3)
Supplement: Additional file 1: Table S1. — Tree model fit comparison. Table S2. Classification of provinces into areas in four region types. Table S3. Results of trait-phylogeny association of different segments. Table S4. Estimation of evolution parameters of different segments. Table S5. Host type and Subtype correlation with Sample Size. Table S6. Transmission rate and statistical support between areas from Traditional Regions. Table S7. Transmission rate and statistical support between areas from Economic Divided Zones. Table S8. Coefficients of predictors of spatial diffusion of Chinese AIV from GLM analysis. Table S9. Sequence information and Accession number of 6 internal segments of AIV in this study. Table S10. Original 31 predictor data per province. (PDF 2305 kb) [file 12862_2016_845_MOESM1_ESM.pdf]

Additional Tables

Table S1 Tree model fit comparison

Models	PS[a]	SS[a]	AICM[b]	HME[c]
Constant size model	-44221	-34077	99146	-49262
Exponential Growth model	-55811	-55185	112154	-54224
Bayesian skyline model	-55779	-55392	109102	-54290
Bayesian skyride model	-47568	-41709	99382	-49275

[a] The estimated log marginal likelihood using path sampling (PS) and stepping stone sampling (SS), lower absolute values of marginal likelihood indicate a better fit to the data.
[b] The likelihood using Akaike's Information Criterion for MCMC samples (AICM), lower values of marginal likelihood indicate a better fit to the data.
[c] The estimated harmonic marginal likelihood of posterior (HME), lower absolute values of marginal likelihood indicate a better fit to the data.

Table S2 Classification of provinces into areas in 4 region types

Region	Area	Provinces
TR	East	Anhui, Fujian, Jiangsu, Jiangxi, Shandong, Shanghai, Zhejiang
	North	Beijing, Hebei, Shanxi
	NorthEast	Heilongjiang, Jilin, Liaoning
	NorthWest	Gansu, Ningxia, Qinghai, Shaanxi, Xinjiang
	SouthCentral	Guangdong, Guangxi, Henan, Hubei, Hunan
	SouthWest	Guizhou, Sichuan, Tibet, Yunnan
ER	Central	Anhui, Henan, Hubei, Hunan, Jiangxi, Shanxi
	EastCoast	Beijing, Fujian, Guangdong, Hebei, Jiangsu, Shandong, Shanghai, Zhejiang
	NorthEast	Heilongjiang, Jilin, Liaoning
	Western	Gansu, Guangxi, Guizhou, Ningxia, Qinghai, Shaanxi, Sichuan, Tibet, Xinjiang, Yunnan
ED	BER (Bohai Economic Rim)	Beijing, Hebei, Shandong
	Central	Anhui, Henan, Hubei, Hunan, Jiangxi, Shanxi
	NorthEast	Heilongjiang, Jilin, Liaoning
	NorthWest	Gansu, Ningxia, Qinghai, Shaanxi, Xinjiang
	PRD (Pan-Pearl River Delta)	Fujian, Guangdong
	SouthWest	Guangxi, Guizhou, Sichuan, Tibet, Yunnan
	YRD (Yangtze River Delta)	Jiangsu, Shanghai, Zhejiang
CAR	Central	Hubei, Hunan, Jiangxi
	East	Anhui, Jiangsu, Shanghai, Zhejiang
	North	Beijing, Hebei, Henan, Shandong, Shanxi
	NorthEast	Heilongjiang, Jilin, Liaoning
	NorthWest	Gansu, Ningxia, Shaanxi, Xinjiang
	Plateau	Qinghai, Tibet
	South	Fujian, Guangdong, Guangxi
	SouthWest	Guizhou, Sichuan, Yunnan

Table S3 Results of trait-phylogeny association for different segments

Statistic			Observed mean	Null mean	P-value
AI		PB2	11.57 (10.8, 12.36)	22.8 (20.6, 24.8)	<0.001
		PB1	14.35 (13.4, 15.25)	23.21 (21.26, 25.22)	<0.001
		PA	13.53 (12.64, 14.34)	22.8 (20.6, 24.8)	<0.001
		NP	12.67 (11.62, 13.74)	22.42 (20.67, 25)	<0.001
		M	15.76 (14.5, 16.71)	22.95(21.14, 24.71)	<0.001
		NS	17.67 (16.43, 18.95)	23.3 (21.46, 25.29)	<0.001
PS		PB2	96.87 (95, 99)	148.6 (141.7, 155.3)	<0.001
		PB1	106.60 (105, 109)	148.5 (141.1, 155.1)	<0.001
		PA	105.06 (102, 108)	148.6 (141.7, 155.3)	<0.001
		NP	103.4 (101, 106)	148.4 (141.45, 154.75)	<0.001
		M	113.2 (110, 116)	148.4 (141.52, 154.9)	<0.001
		NS	117.61 (114, 121)	148.4 (141.96, 154.9)	<0.001
MC	EastCoast	PB2	20 (19.98, 20)	4.14 (3, 6)	0.001
		PB1	12.97 (12.9, 13)	4.2 (3, 6)	0.001
		PA	10.09 (10, 11)	4.15 (3, 6)	0.002
		NP	13.95 (13.9, 14)	4.11 (3, 6)	0.001
		M	9.11 (8, 12)	4.3 (3, 6)	0.005
		NS	7.68 (5, 10)	4.2 (3, 6)	0.002
	Central	PB2	4 (3.9, 4)	3.07 (2, 4)	0.12
		PB1	6.42 (6, 7)	3.11 (2, 4)	0.01
		PA	4.11 (3.9, 4)	3.11 (2, 4.2)	0.13
		NP	6 (6, 6)	3.1 (2.07, 4.17)	0.008
		M	5 (4.9, 5)	3.12 (2.23, 4.21)	0.01
		NS	6.12 (6, 7)	3.13 (2.23, 4.24)	0.004
	Western	PB2	6.99 (6.9, 7)	2.38 (2, 3)	0.1
		PB1	3 (3, 3)	2.38 (2, 3)	0.2
		PA	4 (3.9, 4)	2.38 (2, 3)	0.17
		NP	3 (2.9, 3)	2.37 (2, 3)	0.14
		M	3 (3, 3)	2.37 (2, 3)	0.15
		NS	3 (3, 3)	2.38 (2, 3)	0.14
	Northeast	PB2	3 (2.9, 3)	1.22 (1, 2)	0.001
		PB1	2.02 (1.9, 2)	1.22 (1, 2)	0.1
		PA	3 (3, 3)	1.22 (1, 2)	0.003
		NP	4.27 (3, 7)	1.97 (1.25, 2)	0.001
		M	2.97 (2.9, 3)	1.21 (1, 2)	0.003
		NS	1.34 (1, 2)	1.22 (1, 2)	0.1

Table S4 Estimation of evolution parameters of different segments

	TMRCA (year)		Evo.rate ($\times 10^{-03}$ subs/s/y)	
Segment	mean	(95% HPD lower, upper)	mean	(95% HPD lower, upper)
PB2	1960	(1941, 1977)	4.36	(3.84, 4.92)
PB1	1966	(1951, 1977)	3.15	(2.73, 3.64)
PA	1960	(1941, 1976)	3.41	(2.95, 3.85)
NP	1951	(1935, 1976)	3.32	(2.84, 3.87)
M	1955	(1931, 1975)	2.47	(2.06, 2.90)
NS	1553	(1175, 1836)	1.66	(1.27, 1.96)

Table S5 Host type and Subtype correlation with Sample Size

Number of Host and Subtype Samples		Sample Size	Domestic	Domestic Gal	H5N1	H9	H6
	Total	320	260	107	102	61	76
	Fraction of Total		0.81	0.33	0.32	0.19	0.24
**Region**	**Area**	**Sample Size**	**Domestic**	**Domestic Gal**	**H5N1**	**H9**	**H6**
**TR**	East	119	98	44	22	29	24
	North	14	13	10	5	6	0
	NorthEast	16	6	6	9	2	0
	NorthWest	16	10	10	14	2	0
	SouthCentral	134	115	32	43	20	45
	SouthWest	21	18	5	9	2	7
	Correlation with Sample Size		0.998	0.936	0.894	0.932	0.952
**ER**	Central	98	77	24	36	11	22
	EastCoast	141	125	58	29	41	34
	NorthEast	16	6	6	9	2	0
	Western	65	52	19	28	7	20
	Correlation with Sample Size		0.998	0.940	0.776	0.887	0.972
**ED**	BER	30	27	23	9	18	0
	Central	98	77	24	36	11	22
	NorthEast	16	6	6	9	2	0
	NorthWest	16	10	10	14	2	0
	PRD	65	56	14	12	10	33
	SouthWest	49	42	9	14	5	20
	YRD	46	42	21	8	13	1
	Correlation with Sample Size		0.987	0.562	0.781	0.368	0.763
**CAR**	Central	86	65	15	28	8	22
	East	50	46	22	11	13	1
	North	38	35	31	14	21	0
	NorthEast	16	6	6	9	2	0
	NorthWest	10	10	10	8	2	0
	Plateau	13	4	3	10	2	0
	South	93	80	18	17	13	46
	SouthWest	14	14	2	5	0	7
	Correlation with Sample Size		0.984	0.516	0.839	0.560	0.839

Table S6 Transmission rate and statistical support between areas from Traditional Regions

**PB2**	**Origin**	SouthCentral	East	SouthCentral	SouthCentral
	**Destination**	East	SouthCentral	Northwest	Southwest
	**Mean rate**	0.47	0.24	0.35	0.1
	**Indicator**	1	0.84	0.84	0.84
	**BF**	3703	6	6	
**PB1**	**Origin**	SouthCentral	SouthCentral		
	**Destination**	East	Southwest		
	**Mean rate**	0.49	0.1		
	**Indicator**	1	0.81		
	**BF**	1233	5		
**PA**	**Origin**	SouthCentral	Northeast	SouthCentral	
	**Destination**	East	Northwest	Northwest	
	**Mean rate**	0.33	0.96	0.1	
	**Indicator**	1	0.86	0.89	
	**BF**	426	8	10	
**NP**	**Origin**	SouthCentral	SouthCentral	SouthCentral	
	**Destination**	East	Northwest	Southwest	
	**Mean rate**	0.42	0.13	0.14	
	**Indicator**	0.91	0.88	0.87	
	**BF**	410	9	8	
**M**	**Origin**	SouthCentral	Southwest		
	**Destination**	East	Northwest		
	**Mean rate**	0.44	1.92		
	**Indicator**	1	0.88		
	**BF**	1233	9		
**NS**	**Origin**	SouthCentral	Southwest		
	**Destination**	East	Northwest		
	**Mean rate**	0.49	1.32		
	**Indicator**	1	0.8		
	**BF**	1233	5		

States=6; only transitions with BF support > 5 are shown

Table S7 Transmission rate and statistical support between areas from Economic Development Zones

PB 2	**Origin**	South west	YRD	PRD	Central	Central	South west	South west	PRD
	**Destination**	BER	BER	Central	South west	YRD	North east	North west	YRD
	**mean**	0.40	0.63	0.26	0.55	0.25	0.34	0.21	0.19
	**Indicator**	0.84	1.00	0.99	1.00	0.99	0.85	0.85	0.96
	**BF**	9	3102	158	815	169	10	10	43
PB 1	**Origin**	YRD	Central	Central	Central	North west	PRD	PRD	
	**Destination**	BER	North west	South west	YRD	North east	South west	YRD	
	**mean**	0.73	0.14	0.60	0.19	0.92	0.20	0.18	
	**Indicator**	1.00	0.78	1.00	0.93	0.99	0.73	0.86	
	**BF**	7758	6	1409	21	286	5	10	
PA	**Origin**	YRD	Central	Central	Central	North west	PRD	PRD	
	**Destination**	BER	North west	South west	YRD	North east	South west	YRD	
	**mean**	1.17	0.20	0.61	0.23	0.84	0.26	0.21	
	**Indicator**	1.00	0.86	1.00	0.81	0.96	0.80	0.91	
	**BF**	1722	10	815	7	39	7	18	
NP	**Origin**	South west	YRD	Central	Central	Central	South west	PRD	
	**Destination**	BER	BER	North west	South west	YRD	North east	YRD	
	**mean**	0.26	0.62	0.23	0.50	0.24	0.84	0.24	
	**Indicator**	0.84	1.00	0.98	1.00	0.97	0.79	0.98	
	**BF**	9	1938	76	774	50	7	76	
M	**Origin**	YRD	PRD	Central					
	**Destination**	BER	Central	South west					
	**mean**	0.74	0.41	0.73					
	**Indicator**	0.98	0.89	1.00					
	**BF**	91	15	418					
NS	**Origin**	YRD	Central	PRD	South west				
	**Destination**	BER	South west	South west	YRD				
	**mean**	0.66	0.91	0.39	0.83				
	**Indicator**	0.75	0.99	0.94	0.84				
	**BF**	5	121	26	9				

States=7; only transitions with BF support > 5 are shown

Table S8 Coefficients of predictors of spatial diffusion of Chinese AIV from GLM analysis

Predictor		Segment	TR			ED		
			Coefficient	Indicator	BF	Coefficient	Indicator	BF
**Poultry population density**	Overall	PB2	0.94 (-2.88, 4.78)	0.75	4	2.23 (-0.88, 6.2)	0.75	4
		PB1	0.75 (-3.29, 4.49)	0.74	3	0.8 (-2.28, 4.14)	0.69	3
		PA	0.21 (-3.65, 3.96)	0.77	4	0.65 (-2.1, 4.42)	0.70	3
		NP	0.21 (-3.65, 3.96)	0.77	4	0.71 (-2.28, 4.38)	0.69	3
		M	0.4 (-3.58, 4.01)	0.75	4	0.5 (-2.66, 3.79)	0.68	3
		NS	0.47 (-3.43, 4.38)	0.75	4	0.42 (-2.52, 3.99)	0.68	3
	Origin	PB2	-1.09 (-4.08, 2.15)	0.76	4	0.89 (-1.19, 3.6)	0.76	4
		PB1	-1.27 (-4.18, 1.78)	0.79	5	-0.19 (-2.27, 1.84)	0.58	2
		PA	-2.44 (-4.96, 0.2)	0.95	24	-0.35 (-2.71, 1.59)	0.62	2
		NP	-2.44 (-4.96, 0.2)	0.95	24	-0.24 (-2.74, 2.17)	0.62	2
		M	-2.04 (-4.79, 0.6)	0.91	13	-0.6 (-2.97, 0.99)	0.64	2
		NS	-1.85 (-4.62, 1.67)	0.88	9	-0.66 (-3.14, 0.89)	0.65	2
	Destination	PB2	4.99 (2.88, 7.12)	1	6172	3.91 (1.7, 6.09)	1	6172
		PB1	4.62 (2.28, 6.78)	1	3085	3.07 (0.91, 5.37)	0.99	122
		PA	3.49 (1.27, 5.82)	0.9952	256	2.84 (0.66, 5.14)	0.98	60
		NP	3.49 (1.27, 5.82)	1	256	2.42 (0, 4.4)	0.95	23
		M	3.76 (1.26, 5.96)	0.99	236	3.34 (1.14, 5.53)	0.99	122
		NS	4.01 (1.52, 6.62)	0.99	227	3.08 (0.6, 5.34)	0.98	60
**Farm product market**	Overall	PB2	1.64 (-2.57, 5.72)	0.79	5	0.7 (-2.6, 4.45)	0.79	5
		PB1	1.02 (-2.8, 5.32)	0.73	3	0.24 (-2.95, 3.83)	0.61	2
		PA	0.41 (-3.45, 4.27)	0.71	3	0.11 (-3.22, 3.54)	0.67	3
		NP	0.41 (-3.45, 4.27)	0.71	3	0.35 (-2.91, 3.87)	0.62	2
		M	0.34 (-3.62, 4.31)	0.69	3	0.31 (-3.12, 3.76)	0.69	3
		NS	0.22 (-3.63, 4.19)	0.7	3	0.01 (-3.61, 3.42)	0.70	3
	Origin	PB2	-2.71 (-6.33, 1.24)	0.9	11	-0.52 (-3.96, 2.46)	0.9	11
		PB1	-3.57 (-6.86, 0.02)	0.96	27	-0.68 (-3.95, 1.85)	0.64	2
		PA	-4.88 (-7.97, -1.64)	0.99	166	-1.3 (-4.57, 1.5)	0.74	4
		NP	-4.88 (-7.97, -1.64)	0.99	166	-0.44 (-3.57, 2.1)	0.60	2
		M	-4.89 (-7.94, -1.83)	0.99	195	-1.16 (-4.5, 1.45)	0.74	4
		NS	-5.09 (-8.01, -2.13)	1	293	-2.06 (-5.52, 0.71)	0.85	7

	Destination	PB2	7.63 (5.13, 10.21)	1	6172	2.42 (0, 5.41)	1	6172
		PB1	6.88 (4.4, 9.57)	1	6172	1.27 (-0.82, 4.33)	0.72	3
		PA	6.17 (3.65, 8.64)	1.00	6172	1.71 (-0.6, 4.69)	0.80	5
		NP	6.17 (3.65, 8.64)	1	6172	1.22 (-0.9, 4.22)	0.70	3
		M	6.08 (3.51, 8.63)	1	6172	2.33 (0, 5.27)	0.89	10
		NS	5.93 (3.38, 8.6)	1	6172	2.28 (-0.13, 5.32)	0.88	9
**Stock Volume of Forest**	Overall	PB2	0.35 (-3.38, 4.17)	0.7	3	-0.13 (-3.11, 2.68)	0.7	3
		PB1	-0.07 (-3.98, 3.58)	0.68	3	0.04 (-2.74, 3.13)	0.59	2
		PA	-0.46 (-4.18, 3.32)	0.71	3	-0.37 (-3.56, 2.44)	0.63	2
		NP	-0.46 (-4.18, 3.32)	0.71	3	-0.07 (-2.99, 2.7)	0.59	2
		M	-0.12 (-4.07, 3.57)	0.69	3	0.05 (-2.43, 2.76)	0.41	1
		NS	-0.11 (-4.11, 3.64)	0.69	3	0.16 (-1.79, 2.9)	0.31	1
	Origin	PB2	-0.04 (-3.49, 3.36)	0.67	3	-0.57 (-2.83, 0.93)	0.67	3
		PB1	-0.22 (-3.75, 3.02)	0.65	2	-0.44 (-2.77, 1.06)	0.56	2
		PA	-0.76 (-4.05, 2.65)	0.70	3	-0.88 (-3.21, 0.81)	0.69	3
		NP	-0.76 (-4.05, 2.65)	0.7	3	-0.45 (-2.73, 0.99)	0.57	2
		M	-0.26 (-3.74, 3.11)	0.64	2	-0.4 (-2.75, 0.65)	0.43	1
		NS	-0.23 (-3.57, 2.96)	0.63	2	-0.02 (-1.42, 1.06)	0.23	0
	Destination	PB2	0.48 (-2.86, 3.44)	0.66	2	0.26 (-1.18, 2.18)	0.66	2
		PB1	0.08 (-3.24, 3.13)	0.62	2	0.41 (-0.98, 2.45)	0.56	2
		PA	0.02 (-3.17, 3.12)	0.62	2	0.16 (-1.46, 2.12)	0.57	2
		NP	0.02 (-3.17, 3.12)	0.62	2	0.29 (-1.19, 2.2)	0.54	1
		M	0.06 (-3.27, 3.16)	0.61	2	0.37 (-0.51, 2.38)	0.41	1
		NS	0.01 (-3.1, 3.32)	0.61	2	0.11 (-0.5, 1.59)	0.23	0
**Nature Reserves**	Overall	PB2	-0.17 (-3.97, 3.56)	0.63	2	-0.22 (-3.49, 2.17)	0.63	2
		PB1	-0.13 (-3.82, 3.49)	0.64	2	0.06 (-2.55, 3.06)	0.64	2
		PA	0.07 (-3.91, 3.74)	0.77	4	0.02 (-2.75, 2.8)	0.66	2
		NP	0.07 (-3.91, 3.74)	0.77	4	0.17 (-2.6, 3.16)	0.60	2
		M	0.09 (-3.88, 3.85)	0.74	4	-0.1 (-3.2, 2.52)	0.62	2
		NS	0.05 (-3.84, 3.99)	0.7	3	0.29 (-2.43, 3.66)	0.63	2
	Origin	PB2	0.24 (-3.33, 3.14)	0.51	1	0.34 (-0.94, 1.97)	0.51	1
		PB1	0.28 (-2.95, 3.52)	0.53	1	0.73 (-0.61, 2.2)	0.72	3
		PA	0.82 (-2.22, 3.11)	0.76	4	0.64 (-0.5, 2.19)	0.70	3
		NP	0.82 (-2.22, 3.11)	0.76	4	0.76 (-0.55, 2.35)	0.73	3

		M	0.81 (-2.25, 3.16)	0.73	3	0.49 (-0.67, 2.11)	0.60	2
		NS	0.77 (-2.36, 3.18)	0.71	3	1.3 (-0.24, 2.92)	0.88	9
	Destination	PB2	-1.95 (-3.12, -0.63)	0.98	69	-1.08 (-2.4, 0.16)	0.98	69
		PB1	-1.85 (-2.95, -0.4)	0.98	56	-0.68 (-2.15, 0.54)	0.73	3
		PA	-1.39 (-3.06, 0.48)	0.92	13	-0.76 (-2.17, 0.43)	0.77	4
		NP	-1.39 (-3.06, 0.48)	0.92	13	-0.57 (-2.04, 0.68)	0.69	3
		M	-1.39 (-3.01, 0.84)	0.9	11	-1.02 (-2.25, 0.42)	0.84	6
		NS	-1.36 (-3.06, 1.25)	0.87	8	-0.34 (-2.12, 1.1)	0.59	2
**Average Temperature**	Overall	PB2	0.17 (-3.63, 4.06)	0.59	2	-0.08 (-3.37, 3.23)	0.59	2
		PB1	0.11 (-3.83, 3.76)	0.58	2	-0.49 (-3.03, 1.89)	0.56	2
		PA	-0.08 (-4.12, 4.03)	0.75	4	-0.6 (-3.4, 1.91)	0.67	3
		NP	0 (-4.3, 4.04)	0.68	3	-0.46 (-2.99, 1.98)	0.64	2
		M	-0.02 (-3.72, 4.2)	0.67	3	-0.13 (-2.86, 2.29)	0.56	2
		NS	-0.06 (-3.87, 4.06)	0.68	3	0.32 (-2.07, 3.3)	0.59	2
	Origin	PB2	-0.34 (-3.17, 3.4)	0.51	1	-1.59 (-3.25, 0)	0.88	9
		PB1	-0.41 (-3.47, 3.13)	0.53	1	0.1 (-1.15, 1.62)	0.94	19
		PA	-1.08 (-3.36, 1.65)	0.82	6	0.04 (-1.5, 1.57)	0.99	122
		NP	-0.9 (-3.73, 2.13)	0.72	3	0.11 (-1.24, 1.5)	0.98	60
		M	-0.79 (-3.27, 2.53)	0.7	3	0.35 (-0.79, 1.76)	0.7	3
		NS	-1.02 (-3.79, 2.11)	0.75	4	0.86 (-0.44, 2.27)	0.79	5
	Destination	PB2	3.4 (1.8, 4.8)	1	6172	0.42 (-0.53, 2.22)	0.54	1
		PB1	3.46 (1.83, 4.89)	1	6172	-0.34 (-1.63, 0.45)	0.43	1
		PA	2.55 (0.82, 4.25)	0.99	122	-0.46 (-1.89, 0.46)	0.58	2
		NP	2.9 (0.95, 4.69)	0.99	122	-0.34 (-1.65, 0.51)	0.57	2
		M	2.93 (1.19, 4.44)	1	6172	-0.51 (-1.73, 0.4)	0.6	2
		NS	2.88 (1.02, 4.6)	0.99	122	-0.25 (-1.62, 0.98)	0.55	2
**Average Relative Humidity**	Overall	PB2	0.68 (-3.28, 4.59)	0.74	4	-0.08 (-3.8, 3.34)	0.72	3
		PB1	0.45 (-3.5, 4.31)	0.75	4	-0.37 (-3.88, 2.37)	0.7	3
		PA	0.05 (-3.74, 4.19)	0.76	4	-0.88 (-4.54, 2.35)	0.67	3
		NP	0.4 (-3.59, 4.17)	0.76	4	-0.81 (-4.84, 2.36)	0.67	3
		M	0.25 (-3.64, 4.3)	0.75	4	-0.12 (-3.57, 2.92)	0.69	3
		NS	0.28 (-3.79, 4.03)	0.75	4	-0.26 (-3.86, 2.78)	0.71	3
	Origin	PB2	-2.61 (-6.26, 1.28)	0.9	11	-3.1 (-5.85, 0)	0.95	23
		PB1	-3.46 (-6.75, -0.25)	0.95	23	-1.88 (-3.42, 0)	0.96	30

		PA	-4.04 (-7.35, -0.62)	0.98	60	-2.46 (-4.8, -0.55)	0.98	60
		NP	-3.34 (-6.93, -0.38)	0.95	23	-2.58 (-4.67, 0)	0.96	30
		M	-3.48 (-7.09, 0.14)	0.96	30	-0.79 (-2.5, 0.37)	0.86	8
		NS	-3.96 (-7.3, -0.75)	0.98	60	-0.99 (-2.77, 0.2)	0.94	19
	Destination	PB2	5.92 (2.81, 8.77)	1	6172	2.76 (0, 5.41)	0.93	16
		PB1	5.63 (2.7, 8.81)	1	6172	0.03 (-1.32, 1.65)	0.82	6
		PA	4.21 (1.22, 7.42)	0.99	122	-0.51 (-2.57, 0.7)	0.7	3
		NP	5.13 (2.1, 8.19)	1	6172	-0.56 (-2.85, 0.89)	0.76	4
		M	4.73 (1.76, 7.81)	1	6172	0.46 (-0.53, 2.04)	0.96	30
		NS	5.1 (2.02, 8.05)	1	6172	0.25 (-1.05, 1.88)	0.88	9
**Surface Water Resources**	Overall	PB2	0.27 (-3.33, 4.15)	0.76	4	0.3 (-2.94, 3.54)	0.76	4
		PB1	0.1 (-3.68, 4.07)	0.72	3	0.16 (-2.74, 3.26)	0.60	2
		PA	-0.2 (-4.04, 3.72)	0.73	3	-0.07 (-3.3, 3.19)	0.67	3
		NP	-0.2 (-4.04, 3.72)	0.73	3	0.1 (-2.88, 3.69)	0.62	2
		M	-0.21 (-4.17, 3.59)	0.73	3	0.27 (-2.52, 3.57)	0.58	2
		NS	-0.33 (-4.28, 3.57)	0.73	3	0.2 (-3.18, 3.33)	0.64	2
	Origin	PB2	-1.31 (-4.13, 1.99)	0.83	6	-0.67 (-3.12, 0.87)	0.83	6
		PB1	-1.5 (-4.58, 1.86)	0.82	6	-0.57 (-2.74, 0.8)	0.61	2
		PA	-2.63 (-5, -0.18)	0.97	38	-1.46 (-3.65, 0.12)	0.82	6
		NP	-2.63 (-5, -0.18)	0.97	38	-0.86 (-3.23, 0.67)	0.68	3
		M	-2.7 (-5.3, 0.04)	0.96	28	-0.41 (-2.76, 0.91)	0.53	1
		NS	-3.06 (-5.71, -0.35)	0.97	43	-0.74 (-3.08, 0.83)	0.64	2
	Destination	PB2	2.75 (0.57, 5)	0.98	68	2.16 (0, 3.79)	0.98	68
		PB1	2.28 (-0.31, 4.71)	0.93	17	1.28 (-0.15, 3.18)	0.82	6
		PA	1.41 (-1.55, 4.17)	0.84	7	1.34 (-0.26, 3.54)	0.80	5
		NP	1.41 (-1.55, 4.17)	0.84	7	1.66 (-0.03, 3.76)	0.86	8
		M	1.51 (-1.42, 4.37)	0.85	7	2.17 (0, 3.69)	0.94	19
		NS	1.45 (-1.79, 4.34)	0.83	6	2.13 (0, 3.85)	0.93	16
**Human population density**	Overall	PB2	1.34 (-2.76, 5.33)	0.77	4	1.2 (-1.69, 4.96)	0.77	4
		PB1	0.85 (-3.21, 4.67)	0.75	4	0.5 (-2.1, 3.64)	0.57	2
		PA	0.41 (-3.55, 4.05)	0.74	4	0.3 (-2.71, 3.46)	0.64	2
		NP	0.41 (-3.55, 4.05)	0.74	4	0.35 (-2.37, 3.69)	0.59	2
		M	0.3 (-3.58, 3.92)	0.74	3	0.19 (-2.93, 3.31)	0.59	2
		NS	0.2 (-3.59, 4.04)	0.74	3	-0.1 (-3.46, 2.88)	0.61	2

	Origin	PB2	-0.07 (-3.09, 3.41)	0.66	2	0.38 (-1.62, 2.75)	0.66	2
		PB1	-0.38 (-3.64, 2.81)	0.68	3	-0.14 (-2.15, 1.57)	0.49	1
		PA	-1.34 (-4.38, 2.04)	0.81	5	-0.52 (-2.89, 1.24)	0.63	2
		NP	-1.34 (-4.38, 2.04)	0.81	5	-0.29 (-2.44, 1.52)	0.55	2
		M	-1.28 (-4.46, 2.08)	0.79	5	-0.61 (-3.02, 1.05)	0.61	2
		NS	-1.45 (-4.98, 1.68)	0.81	5	-1.29 (-3.56, 0.63)	0.77	4
	Destination	PB2	3.84 (1.91, 5.62)	1	2056	1.79 (0, 3.69)	1	2056
		PB1	3.48 (1.39, 5.22)	1	513	0.86 (-0.25, 2.84)	0.67	3
		PA	3.15 (1.09, 5.06)	0.99	170	1.3 (-0.24, 3.15)	0.84	6
		NP	3.15 (1.09, 5.06)	0.99	170	0.82 (-0.41, 2.77)	0.68	3
		M	3.1 (0.91, 4.97)	0.99	126	1.25 (-0.25, 3.13)	0.80	5
		NS	2.82 (0.37, 5.02)	0.97	38	0.94 (-0.6, 3.07)	0.71	3
**Freight by transportation**	Overall	PB2	1.69 (-2.38, 5.89)	0.85	7	1.35 (-1.77, 5.46)	0.85	7
		PB1	1.12 (-2.93, 5.16)	0.81	5	0.88 (-2.55, 4.55)	0.74	4
		PA	0.58 (-3.52, 4.33)	0.77	4	0.54 (-2.86, 4.41)	0.77	4
		NP	0.58 (-3.52, 4.33)	0.77	4	0.63 (-2.73, 4.46)	0.75	4
		M	0.51 (-3.43, 4.39)	0.76	4	0.73 (-2.62, 4.43)	0.76	4
		NS	0.35 (-3.76, 4.26)	0.75	4	0.62 (-2.88, 4.23)	0.75	4
	Origin	PB2	-4.71 (-8.32, -1.12)	0.99	92	-1.54 (-4.83, 1.2)	0.99	92
		PB1	-5.59 (-8.94, -2.27)	1	342	-1.93 (-5.37, 0.68)	0.82	6
		PA	-6.2 (-9.77, -2.75)	1	424	-2.97 (-6.18, 0)	0.93	16
		NP	-6.2 (-9.77, -2.75)	1	424	-2.43 (-5.97, 0.3)	0.87	8
		M	-6.45 (-9.78, -3.17)	1	1542	-3.51 (-6.49, 0)	0.96	30
		NS	-7.03 (-10.31, -4.03)	1	6172	-3.45 (-6.44, 0)	0.96	30
	Destination	PB2	8.94 (6.03, 11.81)	1	6172	5.79 (2.57, 9.17)	1	12344
		PB1	8.61 (5.62, 11.43)	1	6172	4.99 (1.53, 8.69)	0.99	122
		PA	7.74 (4.8, 10.47)	1	6172	4.86 (1.57, 8.57)	0.99	122
		NP	7.74 (4.8, 10.47)	1	6172	4.62 (0.96, 8.36)	0.99	122
		M	7.87 (5, 10.76)	1	6172	5.72 (2.6, 9.02)	0.99	6172
		NS	8 (4.97, 10.86)	1	6172	5.3 (1.9, 8.52)	1.00	6172
**Sample size**	Overall	PB2	0.26 (-3.23, 4.12)	0.52	1	0.22 (-2.46, 3.14)	0.52	1
		PB1	0.21 (-3.36, 4.05)	0.49	1	0 (-2.93, 3.1)	0.50	1
		PA	-0.07 (-3.88, 3.83)	0.69	3	-0.03 (-3.3, 3.62)	0.61	2
		NP	-0.07 (-3.88, 3.83)	0.69	3	-0.09 (-3.53, 3.45)	0.59	2

		M	-0.03 (-3.96, 3.76)	0.64	2	0.05 (-3.17, 2.86)	0.51	1
		NS	0.05 (-3.65, 4.09)	0.63	2	-0.07 (-3.32, 3.26)	0.56	2
	Origin	PB2	-0.31 (-3.32, 3.57)	0.46	1	-0.24 (-2, 0.91)	0.46	1
		PB1	-0.36 (-3.47, 3.23)	0.46	1	-0.46 (-2.6, 0.46)	0.48	1
		PA	-1.31 (-3.89, 1.54)	0.84	6	-1.23 (-3.35, 0.04)	0.75	4
		NP	-1.31 (-3.89, 1.54)	0.84	6	-1.18 (-3.43, 0.11)	0.71	3
		M	-1.14 (-3.75, 2.17)	0.78	4	-0.45 (-2.47, 0.55)	0.48	1
		NS	-1.07 (-4.02, 2.31)	0.71	3	-0.82 (-3.14, 0.22)	0.61	2
	Destination	PB2	3.65 (2, 4.88)	1	1028	2.86 (1.4, 4.24)	1	1028
		PB1	3.56 (1.94, 4.76)	1	1121	2.55 (0.65, 4.03)	0.98	60
		PA	0.13 (0, 2.92)	0.84	70	1.77 (0, 3.5)	0.87	8
		NP	2.62 (0, 4.43)	0.98	70	1.84 (0, 3.54)	0.88	9
		M	2.69 (0.81, 4.34)	0.98	68	2.82 (0.97, 4.2)	0.99	122
		NS	2.82 (0.79, 4.6)	0.98	56	2.26 (0, 3.59)	0.94	19

**Proportion of domestic**	Overall	PB2	-0.14 (-3.19, 3.4)	0.61	2	0.02 (-3.76, 4.02)	0.12	0
		PB1	-0.13 (-3.4, 3.16)	0.63	2	-0.09 (-3.97, 3.81)	0.15	0
		PA	-0.09 (-3.53, 3.19)	0.63	2	0 (-3.67, 3.99)	0.13	0
		NP	0.15 (-3.61, 3.77)	0.63	2	-0.01 (-3.84, 4.12)	0.21	0
		M	-0.03 (-3.52, 3.34)	0.62	2	-0.01 (-3.75, 4.02)	0.10	0
		NS	0.3 (-3.28, 3.95)	0.63	2	0.13 (-3.62, 4.08)	0.15	0
	Origin	PB2	0.66 (-2.88, 3.59)	0.68	3	0.04 (-3.83, 3.9)	0.09	0
		PB1	0.94 (-2.35, 3.93)	0.73	3	0.13 (-3.97, 3.66)	0.12	0
		PA	0.83 (-2.59, 3.73)	0.72	3	0.07 (-3.87, 3.73)	0.10	0
		NP	1.22 (-2.75, 4.23)	0.78	4	0.2 (-3.77, 3.66)	0.19	0
		M	0.88 (-2.37, 3.98)	0.72	3	0.03 (-3.97, 3.74)	0.07	0
		NS	1.38 (-2.09, 4.24)	0.81	5	0.01 (-3.85, 3.8)	0.06	0
	Destination	PB2	-0.87 (-3.37, 2.69)	0.73	3	0.03 (-3.97, 3.66)	0.09	0
		PB1	-1.16 (-3.73, 2.13)	0.81	5	0.1 (-3.92, 3.77)	0.14	0
		PA	-1.12 (-3.66, 1.95)	0.81	5	0.06 (-3.76, 3.9)	0.10	0
		NP	-0.88 (-3.64, 2.61)	0.78	4	0.12 (-3.76, 3.72)	0.19	0

		M	-1.21 (-3.74, 2.01)	0.81	5	-0.02 (-3.92, 3.87)	0.07	0
		NS	-0.9 (-3.61, 2.49)	0.76	4	0.01 (-3.89, 3.79)	0.05	0
**Proportion of domestic galliformes**	Overall	PB2	0.41 (-2.95, 3.86)	0.61	2	0.33 (-3.32, 3.7)	0.42	1
		PB1	0.69 (-3.08, 4.07)	0.67	2	0.03 (-3.6, 3.74)	0.29	1
		PA	0.29 (-3.25, 3.53)	0.64	2	0.07 (-3.67, 3.66)	0.29	1
		NP	0.87 (-2.55, 4.45)	0.73	3	0.37 (-3.5, 3.59)	0.50	2
		M	0.6 (-3, 4.04)	0.67	2	0.16 (-3.64, 3.63)	0.34	1
		NS	0.71 (-2.99, 4.19)	0.67	2	0.05 (-3.52, 3.75)	0.30	1
	Origin	PB2	-0.26 (-3.19, 3.11)	0.60	2	0.08 (-3.65, 3.72)	0.24	1
		PB1	-0.21 (-3.04, 3.04)	0.64	2	0.13 (-3.7, 3.62)	0.23	1
		PA	-0.35 (-3.3, 2.77)	0.65	2	0.07 (-3.76, 3.82)	0.22	0
		NP	-0.03 (-2.9, 3.08)	0.68	3	0.2 (-3.53, 3.45)	0.36	1
		M	-0.14 (-3.09, 2.98)	0.65	2	0.09 (-3.75, 3.52)	0.25	1
		NS	-0.1 (-2.94, 3.19)	0.67	2	-0.03 (-3.92, 3.55)	0.22	0
	Destination	PB2	0.8 (-2.15, 3.49)	0.74	4	0.07 (-3.72, 3.57)	0.25	1
		PB1	1.27 (-1.18, 3.84)	0.87	8	0.01 (-3.89, 3.53)	0.21	0
		PA	0.59 (-2.59, 3.29)	0.68	3	0.07 (-3.63, 3.78)	0.20	0
		NP	1.14 (-1.63, 3.58)	0.85	7	0.07 (-3.63, 3.46)	0.30	1
		M	0.83 (-2.35, 3.39)	0.76	4	0.06 (-3.7, 3.62)	0.23	1
		NS	1.12 (-1.65, 3.63)	0.85	7	0.02 (-3.84, 3.64)	0.20	0

For predictors, their correlations to the AIV diffusion in two region types (TR, ED) were summarized and the columns represented six internal segments.
[a]: IP is the abbreviation of the inclusion probabilities, ranging between 0 to 1, representing the less precise values by showing only 2 decimal places.
[b]: the Bayes Factor estimates for the inclusion probabilities with BF>3 indicating a statistical significant (in bold).
[c]: the correlation Coefficient with mean and 95% HPD interval. The mean and intervals above 0 indicates a positive correlation (in red), while the value below 0 indicates a negative correlation (in blue).

Table S9 Sequence information and Accession numbers of 6 internal segments of AIV in this study

Strain Name	Accession No.						Subtype	Host		Provinces
	PB2	PB1	PA	NP	M	NS		Order	Type	
A/chicken/Henan/26/00	DQ064557	DQ064530	DQ064503	DQ064449	DQ064395	DQ064476	H9N2	Galliformes	Domestic	Henan
A/duck/Shantou/1275/2004	HM145575	HM145406	HM145237	HM145068	HM144731	HM144899	H6N1	Anseriformes	Domestic	Guangdong
A/wild duck/Shantou/867/2002	HM145625	HM145456	HM145287	HM145118	HM144780	HM144949	H6N2	Anseriformes	Wild	Guangdong
A/little grebe/Xianghai/429/2011	JX570859	JX570858	JX570857	JX570862	JX570860	JX570861	H5N2	Anseriformes	Wild	Jilin
A/duck/Guangxi/2281/2007	CY109623	CY109624	CY109625	CY109627	CY109629	CY109630	H6N6	Anseriformes	Domestic	Guangxi
A/duck/Guangxi/GXd-1/2009	JX293566	JX293565	JX293564	JX293562	JX293560	JX293563	H6N5	Anseriformes	Domestic	Guangxi
A/duck/Guangxi/GXd-7/2011	JX304777	JX304776	JX304775	JX304773	JX304771	JX304774	H6N6	Anseriformes	Domestic	Guangxi
A/duck/Fujian/378/2007	CY109775	CY109776	CY109777	CY109779	CY109781	CY109782	H6N6	Anseriformes	Domestic	Fujian
A/duck/Guangxi/3574/2006	CY109271	CY109272	CY109273	CY109275	CY109277	CY109278	H6N2	Anseriformes	Domestic	Guangxi
A/wild duck/Shantou/5769/2004	HM145668	HM145499	HM145330	HM145161	HM144823	HM144992	H6N2	Anseriformes	Wild	Guangdong
A/wild duck/Shantou/865/2002	HM145624	HM145455	HM145286	HM145117	HM144779	HM144948	H6N2	Anseriformes	Wild	Guangdong
A/duck/Fujian/2018/2007	CY109807	CY109808	CY109809	CY109811	CY109813	CY109814	H6N6	Anseriformes	Domestic	Fujian
A/wild duck/Shantou/311/2001	HM145740	HM145571	HM145402	HM145233	HM144895	HM145064	H6N9	Anseriformes	Wild	Guangdong
A/duck/Fujian/5813/2007	CY110529	CY110530	CY110531	CY110533	CY110535	CY110536	H6N6	Anseriformes	Domestic	Fujian
A/duck/Shantou/10776/2006	CY109439	CY109440	CY109441	CY109443	CY109445	CY109446	H6N2	Anseriformes	Domestic	Guangdong
A/duck/Fujian/331/2007	CY110653	CY110654	CY110655	CY110657	CY110659	CY110660	H6N6	Anseriformes	Domestic	Fujian
A/duck/Fujian/958/2006	CY109487	CY109488	CY109489	CY109491	CY109493	CY109494	H6N6	Anseriformes	Domestic	Fujian
A/duck/Fujian/11339/2005	HM145714	HM145545	HM145376	HM145207	HM144869	HM145038	H6N2	Anseriformes	Domestic	Fujian
A/duck/Fujian/8807/2006	CY110635	CY110636	CY110637	CY110639	CY110641	CY110642	H6N6	Anseriformes	Domestic	Fujian
A/duck/Shantou/7568/2005	HM145683	HM145514	HM145345	HM145176	HM144838	HM145007	H6N2	Anseriformes	Domestic	Guangdong
A/duck/Hunan/S1661/2012	CY146617	CY146618	CY146619	CY146621	CY146623	CY146624	H6N6	Anseriformes	Domestic	Hunan
A/duck/Hubei/5/2010	CY110970	CY110971	CY110972	CY110974	CY110976	CY110977	H6N6	Anseriformes	Domestic	Hubei
A/duck/Hunan/4056/2006	CY109319	CY109320	CY109321	CY109323	CY109325	CY109326	H6N8	Anseriformes	Domestic	Hunan
A/duck/Shantou/1080/2007	CY109743	CY109744	CY109745	CY109747	CY109749	CY109750	H6N2	Anseriformes	Domestic	Guangdong
A/duck/Shantou/14841/2006	CY109447	CY109448	CY109449	CY109451	CY109453	CY109454	H6N6	Anseriformes	Domestic	Guangdong
A/duck/Shantou/5808/2005	HM145681	HM145512	HM145343	HM145174	HM144836	HM145005	H6N2	Anseriformes	Domestic	Guangdong

A/duck/Shantou/2472/2005	HM145677	HM145508	HM145339	HM145170	HM144832	HM145001	H6N2	Anseriformes	Domestic	Guangdong
A/wild duck/Shantou/9466/2006	CY109431	CY109432	CY109433	CY109435	CY109437	CY109438	H6N6	Anseriformes	Wild	Guangdong
A/duck/Shantou/9689/2006	CY110331	CY110332	CY110333	CY110335	CY110337	CY110338	H6N2	Anseriformes	Domestic	Guangdong
A/duck/Shantou/9395/2006	CY110315	CY110316	CY110317	CY110319	CY110321	CY110322	H6N2	Anseriformes	Domestic	Guangdong
A/duck/Guangdong/W12/2011	JX175250	JX175251	JX175252	JX175254	JX175256	JX175257	H3N2	Anseriformes	Domestic	Guangdong
A/duck/Hebei/0908/2009	JQ041387	JQ041391	JQ041395	JQ041403	JQ041411	JQ041415	H5N2	Anseriformes	Domestic	Hebei
A/duck/Guangdong/E1/2012	JQ924790	JQ924791	JQ924792	JQ924789	JQ924788	JQ924793	H10N8	Anseriformes	Domestic	Guangdong
A/chicken/Jiangsu/1001/2013	KF150631	KF150632	KF150633	KF150635	KF150637	KF150638	H5N2	Galliformes	Domestic	Jiangsu
A/chicken/Guangdong/LG1/2013	KC951119	KC951120	KC951121	KC951123	KC951125	KC951126	H9N2	Galliformes	Domestic	Guangdong
A/chicken/Rizhao/1339/2013	KF260950	KF260706	KF260462	KF259974	KF259455	KF260218	H9N2	Galliformes	Domestic	Shandong
A/silkie chicken/Wenzhou/812/2013	KF260936	KF260692	KF260448	KF259960	KF259441	KF260204	H9N2	Galliformes	Domestic	Zhejiang
A/chicken/Rizhao/55/2013	KF260941	KF260697	KF260453	KF259965	KF259446	KF260209	H9N2	Galliformes	Domestic	Shandong
A/pigeon/Shanghai/JC1/2013	KJ128369	KJ128368	KJ128367	KJ128365	KJ128363	KJ128366	H9N2	Passerine	Wild	Shanghai
A/chicken/Wenzhou/253/2013	KF260932	KF260688	KF260444	KF259956	KF259437	KF260200	H9N2	Galliformes	Domestic	Zhejiang
A/chicken/Shanghai/020/2013	KF500984	KF500983	KF500982	KF500980	KF500978	KF500981	H9N2	Galliformes	Domestic	Shanghai
A/chicken/Guangdong/ZHJ/2011	JN869535	JN869536	JN869537	JN869539	JN869541	JN869542	H9N2	Galliformes	Domestic	Guangdong
A/chicken/Zhejiang/607/2011	JQ356885	JQ356888	JQ356882	JQ356879	JQ356891	JQ356894	H9N2	Galliformes	Domestic	Zhejiang
A/chicken/Wenzhou/89/2013	KF260902	KF260658	KF260414	KF259926	KF259407	KF260170	H7N7	Galliformes	Domestic	Zhejiang
A/chicken/Shandong/03/2010	JF795067	JF795068	JF795069	JF795071	JF795073	JF795074	H9N2	Galliformes	Domestic	Shandong
A/duck/Shanghai/C163/2009	KC768061	KC768058	KC768055	KC768049	KC768043	KC768052	H9N2	Anseriformes	Domestic	Shanghai
A/chicken/Shandong/H/2009	JF795091	JF795092	JF795093	JF795095	JF795097	JF795098	H9N2	Galliformes	Domestic	Shandong
A/chicken/Shandong/02/2010	JF795059	JF795060	JF795061	JF795063	JF795065	JF795066	H9N2	Galliformes	Domestic	Shandong
A/chicken/Hunan/12/2011	KF714780	KF714781	KF714782	KF714784	KF714786	KF714787	H9N2	Galliformes	Domestic	Hunan
A/chicken/Jiangsu/Q3/2010	JN869528	JN869529	JN869530	JN869531	JN869533	JN869534	H9N2	Galliformes	Domestic	Jiangsu
A/chicken/Shanghai/S1080/2013	CY147001	CY147002	CY147003	CY147005	CY147007	CY147008	H7N9	Galliformes	Domestic	Shanghai
A/brambling/Beijing/16/2012	KC464595	KC464596	KC464597	KC464599	KC464601	KC464602	H9N2	Passerine	Wild	Beijing
A/chicken/Shanghai/017/2013	KF542890	KF542888	KF542886	KF542882	KF542878	KF542884	H7N9	Galliformes	Domestic	Shanghai
A/chicken/Zhejiang/SD007/2013	CY147033	CY147034	CY147035	CY147037	CY147039	CY147040	H7N9	Galliformes	Domestic	Zhejiang
A/chicken/Hunan/1/2012	KF714772	KF714773	KF714774	KF714776	KF714778	KF714779	H9N2	Galliformes	Domestic	Hunan
A/chicken/Zhejiang/611/2011	JQ356886	JQ356889	JQ356883	JQ356880	JQ356892	JQ356895	H9N2	Galliformes	Domestic	Zhejiang

A/chicken/Shandong/01/2009	JF795141	JF795142	JF795143	JF795145	JF795147	JF795148	H9N2	Galliformes	Domestic	Shandong
A/chicken/Tibet/S1/2009	CY087168	CY087169	CY087170	CY087172	CY087174	CY087175	H9N2	Galliformes	Domestic	Tibet
A/spot-billed duck/Xianghai/427/2011	JX570843	JX570842	JX570841	JX570846	JX570844	JX570845	H5N2	Anseriformes	Wild	Jilin
A/baikal teal/Xianghai/426/2011	JX570835	JX570834	JX570833	JX570838	JX570836	JX570837	H5N2	Anseriformes	Wild	Jilin
A/duck/Jiangxi/23005/2009	KF260738	KF260494	KF260250	KF259762	KF259243	KF260006	H7N3	Anseriformes	Domestic	Jiangxi
A/duck/Jiangxi/8028/2009	KF260729	KF260485	KF260241	KF259753	KF259234	KF259997	H7N3	Anseriformes	Domestic	Jiangxi
A/chicken/Zhejiang/HJ/2007	FJ581429	FJ581435	FJ581430	FJ581428	FJ581432	FJ581433	H9N2	Galliformes	Domestic	Zhejiang
A/duck/Hunan/2110/2006	CY109311	CY109312	CY109313	CY109315	CY109317	CY109318	H6N2	Anseriformes	Domestic	Hunan
A/duck/Guangxi/2736/2006	CY109255	CY109256	CY109257	CY109259	CY109261	CY109262	H6N8	Anseriformes	Domestic	Guangxi
A/duck/Guangxi/1533/2007	CY109615	CY109616	CY109617	CY109619	CY109621	CY109622	H6N2	Anseriformes	Domestic	Guangxi
A/mallard/Jiangxi/7787/2003	HM145574	HM145405	HM145236	HM145067	HM144730	HM144898	H6N1	Anseriformes	Wild	Jiangxi
A/duck/Guangxi/141/2005	HM145694	HM145525	HM145356	HM145187	HM144849	HM145018	H6N2	Anseriformes	Domestic	Guangxi
A/duck/Guangxi/3459/2005	HM145696	HM145527	HM145358	HM145189	HM144851	HM145020	H6N2	Anseriformes	Domestic	Guangxi
A/duck/Guangxi/1455/2004	HM145717	HM145548	HM145379	HM145210	HM144872	HM145041	H6N5	Anseriformes	Domestic	Guangxi
A/chicken/Fujian/G9/2009	JN869514	JN869515	JN869516	JN869517	JN869519	JN869520	H9N2	Galliformes	Domestic	Fujian
A/sparrow/Guangxi/GXs-1/2012	KF013901	KF013908	KF013907	KF013905	KF013903	KF013906	H1N2	Passerine	Wild	Guangxi
A/duck/Hunan/S11313/2012	CY146569	CY146570	CY146571	CY146573	CY146575	CY146576	H4N2	Anseriformes	Domestic	Hunan
A/duck/Guizhou/1078/2011	KF260748	KF260504	KF260260	KF259772	KF259253	KF260016	H11N9	Anseriformes	Domestic	Guizhou
A/duck/Jiangxi/21980/2010	KF260747	KF260503	KF260259	KF259771	KF259252	KF260015	H7N7	Anseriformes	Domestic	Jiangxi
A/duck/Hunan/S1256/2012	CY146601	CY146602	CY146603	CY146605	CY146607	CY146608	H3N8	Anseriformes	Domestic	Hunan
A/wild goose/Dongting/C1037/2011	KC876688	KC876689	KC876690	KC876692	KC876694	KC876695	H12N8	Anseriformes	Wild	Hunan
A/wild waterfowl/Dongting/C2383/2012	KF874478	KF874479	KF874480	KF874482	KF874484	KF874485	H1N2	Anseriformes	Wild	Hunan
A/wild duck/Jiangxi/8462/2006	CY109335	CY109336	CY109337	CY109339	CY109341	CY109342	H6N1	Anseriformes	Wild	Jiangxi
A/canvasback/Xianghai/428/2011	JX570851	JX570850	JX570849	JX570854	JX570852	JX570853	H5N2	Anseriformes	Wild	Jilin
A/duck/Shanghai/C84/2009	JX286598	JX286597	JX286596	JX286594	JX286592	JX286595	H3N2	Anseriformes	Domestic	Shanghai
A/chicken/Hubei/2856/2007	FJ784835	FJ784819	FJ784803	FJ784787	FJ784883	FJ784867	H5N1	Galliformes	Domestic	Hubei
A/chicken/Hunan/3157/2006	FJ784838	FJ784822	FJ784806	FJ784790	FJ784886	FJ784870	H5N1	Galliformes	Domestic	Hunan
A/duck/Hubei/2911/2007	FJ784836	FJ784820	FJ784804	FJ784788	FJ784884	FJ784868	H5N1	Anseriformes	Domestic	Hubei
A/duck/Hunan/1590/2007	CY109679	CY109680	CY109681	CY109683	CY109685	CY109686	H6N9	Anseriformes	Domestic	Hunan
A/wild duck/Jiangxi/9157/2005	KF260785	KF260541	KF260297	KF259809	KF259290	KF260053	H7N8	Anseriformes	Wild	Jiangxi

A/wild duck/Jiangxi/10179/2005	KF260786	KF260542	KF260298	KF259810	KF259291	KF260054	H7N3	Anseriformes	Wild	Jiangxi
A/duck/Hunan/S1824/2012	CY146625	CY146626	CY146627	CY146629	CY146631	CY146632	H3N8	Anseriformes	Domestic	Hunan
A/duck/Hunan/3748/2004	HM145737	HM145568	HM145399	HM145230	HM144892	HM145061	H6N8	Anseriformes	Domestic	Hunan
A/duck/Guizhou/2492/2007	CY109655	CY109656	CY109657	CY109659	CY109661	CY109662	H6N1	Anseriformes	Domestic	Guizhou
A/duck/Jiangsu/26/2004	KC261666	KC261667	KC261668	KC261670	KC261672	KC261673	H3N2	Anseriformes	Domestic	Jiangsu
A/mallard/Jiangxi/12147/2005	HM145700	HM145531	HM145362	HM145193	HM144855	HM145024	H6N2	Anseriformes	Wild	Jiangxi
A/duck/Guizhou/888/2006	CY109279	CY109280	CY109281	CY109283	CY109285	CY109286	H6N5	Anseriformes	Domestic	Guizhou
A/duck/Guizhou/1084/2006	CY109287	CY109288	CY109289	CY109291	CY109293	CY109294	H6N2	Anseriformes	Domestic	Guizhou
A/duck/Shanghai/Y20/2006	CY111603	CY111604	CY111605	CY111607	CY111609	CY111610	H4N6	Anseriformes	Domestic	Shanghai
A/garganey/SanJiang/160/2006	EF634329	EF634330	EF634331	EF634333	EF634335	EF634336	H5N2	Anseriformes	Wild	Guangxi
A/green-winged teal/Xianghai/430/2011	JX570867	JX570866	JX570865	JX570870	JX570868	JX570869	H5N2	Anseriformes	Wild	Jilin
A/wild goose/Dongting/PC0360/2012	KC876680	KC876681	KC876682	KC876684	KC876686	KC876687	H7N7	Anseriformes	Wild	Hunan
A/mallard/Jiangxi/6845/2003	HM145573	HM145404	HM145235	HM145066	HM144729	HM144897	H6N1	Anseriformes	Wild	Jiangxi
A/duck/Hunan/5613/2003	HM145716	HM145547	HM145378	HM145209	HM144871	HM145040	H6N5	Anseriformes	Domestic	Hunan
A/duck/Hunan/1469/2002	HM145735	HM145566	HM145397	HM145228	HM144890	HM145059	H6N8	Anseriformes	Domestic	Hunan
A/chicken/Shaanxi/11/2012	KC767264	KC767257	KC767258	KC767260	KC767262	KC767263	H9N2	Galliformes	Domestic	Shaanxi
A/chicken/Henan/43/02	DQ064558	DQ064531	DQ064504	DQ064450	DQ064396	DQ064477	H9N2	Galliformes	Domestic	Henan
A/chicken/Guangdong/V/2008	JQ639783	JQ639784	JQ639785	JQ639787	JQ639789	JQ639790	H9N2	Galliformes	Domestic	Guangdong
A/chicken/Guangdong/47/01	DQ064547	DQ064521	DQ064494	DQ064440	DQ064386	DQ064467	H9N2	Galliformes	Domestic	Guangdong
A/chicken/Guangdong/10/00	DQ064545	DQ064518	DQ064491	DQ064437	DQ064383	DQ064464	H9N2	Galliformes	Domestic	Guangdong
A/chicken/Shandong/1/2008	JQ904459	JQ904460	JQ904461	JQ904462	JQ904463	JQ904464	H9N2	Galliformes	Domestic	Shandong
A/chicken/Fujian/25/00	DQ064544	DQ064517	DQ064490	DQ064436	DQ064382	DQ064463	H9N2	Galliformes	Domestic	Fujian
A/chicken/Shijiazhuang/2/98	DQ064568	DQ064541	DQ064514	DQ064460	DQ064406	DQ064487	H9N2	Galliformes	Domestic	Hebei
A/chicken/Guangdong/5/97	DQ064549	DQ064522	DQ064495	DQ064441	DQ064387	DQ064468	H9N2	Galliformes	Domestic	Guangdong
A/chicken/Gansu/2/99	EF070740	EF070739	EF070738	EF070735	EF070737	EF070736	H9N2	Galliformes	Domestic	Gansu
A/chicken/Hebei/L1/2006	EU914201	EU882861	EU532061	EU532044	EU532036	EU532055	H9N2	Galliformes	Domestic	Hebei
A/chicken/Shandong/02/2008	JF795043	JF795044	JF795045	JF795047	JF795049	JF795050	H9N2	Galliformes	Domestic	Shandong
A/chicken/Shandong/KD/2009	JF795099	JF795100	JF795101	JF795103	JF795105	JF795106	H9N2	Galliformes	Domestic	Shandong
A/chicken/Hebei/7/2008	GQ202053	GQ202052	GQ202055	GQ202054	GQ202051	GQ202050	H9N2	Galliformes	Domestic	Hebei
A/chicken/Hebei/C4/2008	GQ202047	GQ202046	GQ202049	GQ202048	GQ202045	GQ202044	H9N2	Galliformes	Domestic	Hebei

A/chicken/Shandong/LY-1/2008	JF795107	JF795108	JF795109	JF795111	JF795113	JF795114	H9N2	Galliformes	Domestic	Shandong
A/chicken/Guangdong/56/01	DQ064550	DQ064523	DQ064496	DQ064442	DQ064388	DQ064469	H9N2	Galliformes	Domestic	Guangdong
A/chicken/Guangxi/37/2005	EU086233	EU086232	EU086231	EU086229	EU086227	EU086230	H9N2	Galliformes	Domestic	Guangxi
A/chicken/Heilongjiang/48/01	DQ064556	DQ064529	DQ064502	DQ064448	DQ064394	DQ064475	H9N2	Galliformes	Domestic	Heilongjiang
A/chicken/Hubei/C1/2007	EU365368	EU365369	EU365370	EU365372	EU365374	EU365375	H9N2	Galliformes	Domestic	Hubei
A/chicken/Jiangsu/7/2002	FJ384748	FJ384749	FJ384750	FJ384752	FJ384754	FJ384755	H9N2	Galliformes	Domestic	Jiangsu
A/duck/Wenzhou/771/2013	KF260901	KF260657	KF260413	KF259925	KF259406	KF260169	H7N3	Anseriformes	Domestic	Zhejiang
A/chicken/Jiangsu/RD5/2013	KF006411	KF006412	KF006413	KF006415	KF006417	KF006418	H10N9	Galliformes	Domestic	Jiangsu
A/goose/Guangdong/7472/2012	KF260721	KF260477	KF260233	KF259745	KF259226	KF259989	H7N7	Anseriformes	Domestic	Guangdong
A/duck/Jiangsu/10-d4/2011	CY125004	CY125005	CY125006	CY125008	CY125010	CY125011	H11N3	Anseriformes	Domestic	Jiangsu
A/duck/Zhejiang/0224-6/2011	JN605400	JN605396	JN605392	JN605384	JN605376	JN605388	H1N2	Anseriformes	Domestic	Zhejiang
A/duck/Shanghai/28-1/2009	JX485433	JX485432	JX485431	JX485429	JX485427	JX485430	H4N2	Anseriformes	Domestic	Shanghai
A/duck/Jiangxi/21714/2011	KF260751	KF260507	KF260263	KF259775	KF259256	KF260019	H11N9	Anseriformes	Domestic	Jiangxi
A/duck/Zhejiang/5/2011	JX051226	JX051227	JX051228	JX051230	JX051232	JX051233	H3N3	Anseriformes	Domestic	Zhejiang
A/chicken/Guangxi/12/2004	DQ366327	DQ366328	DQ366329	DQ366331	DQ366333	DQ366334	H5N1	Galliformes	Domestic	Guangxi
A/duck/Anhui/56/2005	HM172441	HM172402	HM172322	HM172227	HM172152	HM172270	H5N1	Anseriformes	Domestic	Anhui
A/duck/Hunan/3340/2006	FJ784840	FJ784824	FJ784808	FJ784792	FJ784888	FJ784872	H5N1	Anseriformes	Domestic	Hunan
A/chicken/Shandong/A-1/2009	HM172414	HM172363	HM172329	HM172252	HM172153	HM172283	H5N1	Galliformes	Domestic	Shandong
A/chicken/Hunan/1/2009	HM172425	HM172367	HM172333	HM172255	HM172150	HM172268	H5N1	Galliformes	Domestic	Hunan
A/wild duck/Shantou/1737/2000	CY031006	EF597452	EF597417	EF597347	AY651429	AY651593	H6N8	Anseriformes	Wild	Guangdong
A/duck/Shandong/093/2004	AY856861	AY856862	AY856863	AY856864	AY856865	AY856866	H5N1	Anseriformes	Domestic	Shandong
A/duck/Guangxi/12/2003	EU263342	EU263343	EU263344	EU263346	EU263348	EU263349	H5N1	Anseriformes	Domestic	Guangxi
A/duck/Guangxi/27/2003	EU263350	EU263351	EU263352	EU263354	EU263356	EU263357	H5N1	Anseriformes	Domestic	Guangxi
A/duck/Guangdong/wy24/2008	CY091640	CY091641	CY091642	CY091644	CY091646	CY091647	H5N5	Anseriformes	Domestic	Guangdong
A/duck/Guangdong/wy19/2008	CY091632	CY091633	CY091634	CY091636	CY091638	CY091639	H5N5	Anseriformes	Domestic	Guangdong
A/duck/Jiangsu/m234/2012	JX507352	JX507353	JX507354	JX507356	JX507358	JX507359	H5N2	Anseriformes	Domestic	Jiangsu
A/wild duck/Shandong/628/2011	JX534562	JX534563	JX534564	JX534566	JX534568	JX534569	H5N1	Anseriformes	Wild	Shandong
A/duck/Jiangsu/1-15/2011	KC282876	KC282877	KC282878	KC282880	KC282882	KC282883	H4N2	Anseriformes	Domestic	Jiangsu
A/goose/Guangdong/k0103/2010	JQ973683	JQ973684	JQ973685	JQ973687	JQ973689	JQ973690	H5N5	Anseriformes	Domestic	Guangdong
A/duck/Shantou/728/2001	HM145588	HM145419	HM145250	HM145081	HM144744	HM144912	H6N2	Anseriformes	Domestic	Guangdong

A/quail/Jiangsu/k0104/2010	JQ973675	JQ973676	JQ973677	JQ973679	JQ973681	JQ973682	H5N5	Galliformes	Domestic	Jiangsu
A/goose/Guangdong/xb/2001	DQ997528	DQ997527	DQ997526	DQ997524	DQ997521	DQ997525	H5N1	Anseriformes	Domestic	Guangdong
A/duck/Jiangxi/5748/2006	CY109327	CY109328	CY109329	CY109331	CY109333	CY109334	H6N2	Anseriformes	Domestic	Jiangxi
A/duck/Jiangxi/21669/2009	KF260737	KF260493	KF260249	KF259761	KF259242	KF260005	H7N7	Anseriformes	Domestic	Jiangxi
A/wild duck/Jiangxi/19831/2009	KF260736	KF260492	KF260248	KF259760	KF259241	KF260004	H7N7	Anseriformes	Wild	Jiangxi
A/duck/Jiangxi/23008/2009	KF260739	KF260495	KF260251	KF259763	KF259244	KF260007	H7N7	Anseriformes	Domestic	Jiangxi
A/duck/Hunan/S1607/2012	CY146609	CY146610	CY146611	CY146613	CY146615	CY146616	H11N9	Anseriformes	Domestic	Hunan
A/chicken/Guangxi/GXc-1/2011	KF013909	KF013916	KF013915	KF013913	KF013911	KF013914	H1N2	Galliformes	Domestic	Guangxi
A/duck/Guangxi/GXd-4/2011	KF013925	KF013932	KF013931	KF013929	KF013927	KF013930	H1N2	Anseriformes	Domestic	Guangxi
A/duck/Guangxi/GXd-1/2011	KF013917	KF013924	KF013923	KF013921	KF013919	KF013922	H1N2	Anseriformes	Domestic	Guangxi
A/duck/Guizhou/1560/2007	CY109647	CY109648	CY109649	CY109651	CY109653	CY109654	H6N8	Anseriformes	Domestic	Guizhou
A/chicken/Jiangxi/2369/2010	KF260742	KF260498	KF260254	KF259766	KF259247	KF260010	H7N7	Galliformes	Domestic	Jiangxi
A/duck/Nanjing/1102/2010	KC683700	KC683701	KC683702	KC683704	KC683706	KC683707	H4N8	Anseriformes	Domestic	Jiangsu
A/domestic green-winged teal/Hunan/3450/2006	KC690153	KC690154	KC690155	KC690157	KC690159	KC690160	H5N1	Anseriformes	Domestic	Hunan
A/duck/Guizhou/1073/2007	CY109631	CY109632	CY109633	CY109635	CY109637	CY109638	H6N8	Anseriformes	Domestic	Guizhou
A/duck/Yunnan/1282/2007	KF260775	KF260531	KF260287	KF259799	KF259280	KF260043	H11N9	Anseriformes	Domestic	Yunnan
A/duck/Guangxi/912/2008	CY076889	CY076890	CY076891	CY076893	CY076895	CY076896	H4N2	Anseriformes	Domestic	Guangxi
A/mallard/Jiangxi/6285/2004	HM145738	HM145569	HM145400	HM145231	HM144893	HM145062	H6N8	Anseriformes	Wild	Jiangxi
A/mallard/Jiangxi/10071/2005	HM145577	HM145408	HM145239	HM145070	HM144733	HM144901	H6N1	Anseriformes	Wild	Jiangxi
A/duck/Jiangxi/22041/2008	KF260749	KF260505	KF260261	KF259773	KF259254	KF260017	H4N9	Anseriformes	Domestic	Jiangxi
A/duck/Guangdong/4323/2007	KF260766	KF260522	KF260278	KF259790	KF259271	KF260034	H11N9	Anseriformes	Domestic	Guangdong
A/duck/Beijing/61/05	EU492492	EU492498	EU492504	EU492516	EU492528	EU492510	H3N8	Anseriformes	Domestic	Beijing
A/duck/Beijing/40/04	EU492488	EU492494	EU492500	EU492512	EU492524	EU492506	H3N8	Anseriformes	Domestic	Beijing
A/duck/Hunan/8-19/2009	HQ285883	HQ285884	HQ285885	HQ285887	HQ285889	HQ285890	H4N2	Anseriformes	Domestic	Hunan
A/duck/Guangxi/GXd-2/2012	KF013933	KF013940	KF013939	KF013937	KF013935	KF013938	H1N2	Anseriformes	Domestic	Guangxi
A/duck/Hunan/S11547/2012	CY146577	CY146578	CY146579	CY146581	CY146583	CY146584	H4N9	Anseriformes	Domestic	Hunan
A/egret/Hunan/1/2012	JX437691	JX437686	JX437688	JX437689	JX437684	JX437687	H9N2	Pelecaniformes	Wild	Hunan
A/baikal teal/Xianghai/421/2011	KC162238	KC162237	KC162236	KC162241	KC162239	KC162240	H9N2	Anseriformes	Wild	Jilin
A/duck/Hunan/S11200/2012	CY146553	CY146554	CY146555	CY146557	CY146559	CY146560	H4N6	Anseriformes	Domestic	Hunan
A/chicken/Jilin/xw/2003	DQ997546	DQ997545	DQ997551	DQ997549	DQ997552	DQ997550	H5N1	Galliformes	Domestic	Jilin

A/chicken/Guangdong/178/04	AY737293	AY737294	AY737295	AY737297	AY737298	AY737300	H5N1	Galliformes	Domestic	Guangdong
A/duck/Guangdong/23/2004	HM172432	HM172387	HM172321	HM172230	HM172140	HM172300	H5N1	Anseriformes	Domestic	Guangdong
A/duck/Hunan/70/2004	HM172416	HM172389	HM172326	HM172231	HM172158	HM172306	H5N1	Anseriformes	Domestic	Hunan
A/goose/Fujian/bb/2003	DQ997404	DQ997403	DQ997401	DQ997407	DQ997402	DQ997408	H5N1	Anseriformes	Domestic	Fujian
A/duck/Hunan/69/2004	HM172422	HM172384	HM172325	HM172229	HM172155	HM172307	H5N1	Anseriformes	Domestic	Hunan
A/chicken/Xinjiang/16/2005	HM172420	HM172383	HM172351	HM172219	HM172125	HM172267	H5N1	Galliformes	Domestic	Xinjiang
A/chicken/Anhui/39/2004	HM172430	HM172390	HM172320	HM172237	HM172123	HM172287	H5N1	Galliformes	Domestic	Anhui
A/shrike/Tibet/13/2006	HM172410	HM172364	HM172341	HM172222	HM172131	HM172281	H5N1	Passerine	Wild	Tibet
A/wild duck/Hunan/021/2005	EU329174	EU329175	EU329176	EU329178	EU329180	EU329181	H5N1	Anseriformes	Wild	Hunan
A/duck/Yunnan/5310/2006	CY030886	CY030887	CY030888	CY030890	CY030892	CY030893	H5N1	Anseriformes	Domestic	Yunnan
A/duck/Hubei/Hangmei01/2006	EU594346	EU594347	EU594348	EU594350	EU594352	EU594353	H5N1	Anseriformes	Domestic	Hubei
A/duck/Hubei/49/2005	HM172449	HM172360	HM172311	HM172216	HM172119	HM172263	H5N1	Anseriformes	Domestic	Hubei
A/chicken/Hunan/21/2005	HM172427	HM172393	HM172353	HM172233	HM172141	HM172293	H5N1	Galliformes	Domestic	Hunan
A/duck/Hubei/xn/2007	KJ003984	KJ003985	KJ003986	KJ003988	KJ003990	KJ003991	H5N1	Anseriformes	Domestic	Hubei
A/goose/Hubei/65/2005	HM172450	HM172358	HM172312	HM172215	HM172117	HM172262	H5N1	Anseriformes	Domestic	Hubei
A/chicken/Sichuan/81/2005	HM172452	HM172357	HM172309	HM172214	HM172118	HM172261	H5N1	Galliformes	Domestic	Sichuan
A/chicken/Hunan/41/2004	HM172428	HM172404	HM172319	HM172224	HM172130	HM172303	H5N1	Galliformes	Domestic	Hunan
A/chicken/Xinjiang/17/2005	HM172435	HM172399	HM172345	HM172225	HM172161	HM172276	H5N1	Galliformes	Domestic	Xinjiang
A/duck/Jiangxi/80/2005	HM172437	HM172391	HM172339	HM172242	HM172151	HM172290	H5N1	Anseriformes	Domestic	Jiangxi
A/duck/Guangdong/173/04	AY737301	AY737302	AY737303	AY737305	AY737306	AY737307	H5N1	Anseriformes	Domestic	Guangdong
A/chicken/Xinjiang/54/2005	HM172439	HM172400	HM172347	HM172247	HM172142	HM172305	H5N1	Galliformes	Domestic	Xinjiang
A/chicken/Jiangxi/25/2004	HM172483	HM172479	HM172475	HM172467	HM172459	HM172471	H5N1	Galliformes	Domestic	Jiangxi
A/ostrich/Suzhou/097/2003	JF732743	JF732745	JF732741	JF732744	JF732740	JF732742	H5N1	Struthioniformes	Domestic	Jiangsu
A/chicken/Xinjiang/67/2005	HM172436	HM172398	HM172349	HM172239	HM172154	HM172302	H5N1	Galliformes	Domestic	Xinjiang
A/chicken/Xinjiang/28/2006	HM172445	HM172386	HM172346	HM172248	HM172144	HM172289	H5N1	Galliformes	Domestic	Xinjiang
A/chicken/Xinjiang/78/2005	HM172446	HM172397	HM172343	HM172246	HM172143	HM172278	H5N1	Galliformes	Domestic	Xinjiang
A/duck/Guangxi/xa/2001	DQ997519	DQ997518	DQ997517	DQ997515	DQ997520	DQ997516	H5N1	Anseriformes	Domestic	Guangxi
A/chicken/Xinjiang/68/2005	HM172447	HM172392	HM172348	HM172256	HM172145	HM172299	H5N1	Galliformes	Domestic	Xinjiang
A/parrot/Guangdong/C99/2005	JX013487	JX013488	JX013489	JX013490	JX013491	JX013492	H5N1	Psittaciformes	Domestic	Guangdong
A/duck/Shanghai/xj/2002	DQ997530	DQ997529	DQ997535	DQ997533	DQ997536	DQ997534	H5N1	Anseriformes	Domestic	Shanghai

A/duck/Zhejiang/bj/2002	DQ997416	DQ997415	DQ997414	DQ997412	DQ997409	DQ997413	H5N1	Anseriformes	Domestic	Zhejiang
A/duck/Hubei/wp/2003	DQ997169	DQ997168	DQ997167	DQ997165	DQ997170	DQ997166	H5N1	Anseriformes	Domestic	Hubei
A/duck/Anhui/1/06	HM172409	HM172401	HM172356	HM172238	HM172133	HM172304	H5N1	Anseriformes	Domestic	Anhui
A/chicken/Jiangsu/18/2008	HM172417	HM172368	HM172352	HM172234	HM172134	HM172275	H5N1	Galliformes	Domestic	Jiangsu
A/chicken/Sheny/0606/2008	JQ277222	JQ277223	JQ277224	JQ277226	JQ277228	JQ277229	H5N1	Galliformes	Domestic	Liaoning
A/chicken/Shandong/A-5/2006	HM172444	HM172382	HM172316	HM172235	HM172164	HM172296	H5N1	Galliformes	Domestic	Shandong
A/chicken/Shanxi/2/2006	DQ914812	DQ914811	DQ914813	DQ914815	DQ914817	DQ914818	H5N1	Galliformes	Domestic	Shanxi
A/chicken/Shanxi/10/2006	HM172440	HM172361	HM172337	HM172243	HM172135	HM172297	H5N1	Galliformes	Domestic	Shanxi
A/chicken/Shandong/A-10/2006	HM172418	HM172376	HM172327	HM172244	HM172139	HM172274	H5N1	Galliformes	Domestic	Shandong
A/chicken/Liaoning/A-11/2006	HM172431	HM172362	HM172355	HM172250	HM172148	HM172308	H5N1	Galliformes	Domestic	Liaoning
A/chicken/Hebei/102/2005	EU243139	EU243138	EU243137	EU243135	EF175672	EU243136	H5N1	Galliformes	Domestic	Hebei
A/chicken/Henan/A-7/2006	HM172434	HM172374	HM172340	HM172236	HM172127	HM172291	H5N1	Galliformes	Domestic	Henan
A/chicken/Hebei/126/2005	EU243140	EU243141	EU243142	EU243144	EF175673	EU243143	H5N1	Galliformes	Domestic	Hebei
A/chicken/Hebei/326/2005	DQ351871	DQ351875	DQ351868	DQ351865	DQ351859	DQ351862	H5N1	Galliformes	Domestic	Hebei
A/duck/Guangxi/GXd-5/2010	JX304761	JX304760	JX304759	JX304757	JX304755	JX304758	H6N1	Anseriformes	Domestic	Guangxi
A/duck/Hunan/S4234/2011	CY146705	CY146706	CY146707	CY146709	CY146711	CY146712	H5N1	Anseriformes	Domestic	Hunan
A/wild duck/Jilin/HF/2011	JX534570	JX534571	JX534572	JX534574	JX534576	JX534577	H5N1	Anseriformes	Wild	Jilin
A/wild duck/Jilin/ZF/2011	JX534578	JX534579	JX534580	JX534582	JX534584	JX534585	H5N1	Anseriformes	Wild	Jilin
A/chicken/Jiangsu/k0402/2010	JQ638687	JQ638685	JQ638683	JQ638679	JQ638675	JQ638681	H5N1	Galliformes	Domestic	Jiangsu
A/wild duck/Shandong/2/2011	JX534554	JX534555	JX534556	JX534558	JX534560	JX534561	H5N1	Anseriformes	Wild	Shandong
A/goose/Jiangsu/k0403/2010	JQ638688	JQ638686	JQ638684	JQ638680	JQ638676	JQ638682	H5N1	Anseriformes	Domestic	Jiangsu
A/great crested-grebe/Qinghai/1/2009	CY063315	CY063316	CY063317	CY063319	CY063321	CY063322	H5N1	Anseriformes	Wild	Qinghai
A/brown-headed gull/Qinghai/9/2009	GU477557	GU477554	GU477551	GU477545	GU477539	GU477536	H5N1	Charadriiformes	Wild	Qinghai
A/great black-headed gull/Qinghai/8/2009	GU477556	GU477553	GU477550	GU477544	GU477538	GU477535	H5N1	Charadriiformes	Wild	Qinghai
A/chicken/Tibet/6/2008	HM172426	HM172380	HM172328	HM172258	HM172162	HM172282	H5N1	Galliformes	Domestic	Tibet
A/chicken/Guizhou/7/2008	HM172424	HM172370	HM172323	HM172259	HM172157	HM172279	H5N1	Galliformes	Domestic	Guizhou
A/duck/Hunan/8/2008	GU182163	GU182164	GU182165	GU182167	GU182169	GU182170	H5N1	Anseriformes	Domestic	Hunan
A/swan/Shanghai/10/2009	JF975558	JF975559	JF975560	JF975562	JF975564	JF975565	H5N1	Anseriformes	Wild	Shanghai
A/bar-headed goose/Qinghai/1-HVRI/2006	HM172415	HM172365	HM172332	HM172218	HM172132	HM172284	H5N1	Anseriformes	Wild	Qinghai
A/bar-headed goose/Qinghai/3/2005	HM172481	HM172477	HM172474	HM172465	HM172457	HM172470	H5N1	Anseriformes	Wild	Qinghai

A/wild duck/Liaoning/8/2006	HM172423	HM172403	HM172314	HM172226	HM172146	HM172269	H5N1	Anseriformes	Wild	Liaoning
A/chicken/Liaoning/23/2005	HM172482	HM172478	HM172473	HM172466	HM172458	HM172469	H5N1	Galliformes	Domestic	Liaoning
A/pied magpie/Liaoning/7/2006	HM172484	HM172480	HM172476	HM172468	HM172460	HM172472	H5N1	Passerine	Wild	Liaoning
A/bar-headed goose/Tibet/8/2006	HM172419	HM172372	HM172318	HM172249	HM172128	HM172277	H5N1	Anseriformes	Wild	Tibet
A/great cormorant/Tibet/12/2006	HM172408	HM172378	HM172317	HM172253	HM172121	HM172292	H5N1	Anseriformes	Wild	Tibet
A/chicken/Tibet/LZ01/2010	JX565016	JX565017	JX565018	JX565020	JX565022	JX565023	H5N2	Galliformes	Domestic	Tibet
A/chicken/Hunan/8/2008	GU182155	GU182156	GU182157	GU182159	GU182162	GU182161	H5N1	Galliformes	Domestic	Hunan
A/chicken/Hunan/3/2007	GU182139	GU182140	GU182141	GU182143	GU182145	GU182146	H5N1	Galliformes	Domestic	Hunan
A/duck/Hunan/3/2007	GU182147	GU182148	GU182149	GU182151	GU182153	GU182154	H5N1	Anseriformes	Domestic	Hunan
A/duck/Hunan/11/2007	HM172407	HM172381	HM172331	HM172245	HM172160	HM172273	H5N1	Anseriformes	Domestic	Hunan
A/chicken/Fujian/1/2007	HM172421	HM172373	HM172335	HM172223	HM172122	HM172272	H5N1	Galliformes	Domestic	Fujian
A/duck/Hunan/29/2006	HM172405	HM172379	HM172334	HM172221	HM172129	HM172294	H5N1	Anseriformes	Domestic	Hunan
A/goose/Yunnan/3798/2006	CY030902	CY030903	CY030904	CY030906	CY030908	CY030909	H5N1	Anseriformes	Domestic	Yunnan
A/goose/Yunnan/4371/2006	CY030910	CY030911	CY030912	CY030914	CY030916	CY030917	H5N1	Anseriformes	Domestic	Yunnan
A/goose/Guangdong/1/1996	AF144300	AF144301	AF144302	AF144303	AF144306	AF144307	H5N1	Anseriformes	Domestic	Guangdong
A/duck/Fujian/5476/2008	KF260723	KF260479	KF260235	KF259747	KF259228	KF259991	H7N7	Anseriformes	Domestic	Fujian
A/mallard/Jiangxi/10668/2005	HM145578	HM145409	HM145240	HM145071	HM144734	HM144902	H6N1	Anseriformes	Wild	Jiangxi
A/black-billed magpie/Guangxi/29/2005	GU121379	GU121380	GU121381	GU121383	GU121385	GU121386	H9N2	Passerine	Wild	Guangxi
A/chicken/Jilin/hd/2002	DQ997289	DQ997288	DQ997287	DQ997285	DQ997290	DQ997286	H5N1	Galliformes	Domestic	Jilin
A/chicken/Guangdong/1/2005	EU874902	EU874903	EU874897	EU874898	EU874901	EU874904	H5N1	Galliformes	Domestic	Guangdong
A/duck/Fujian/7794/2007	CY109895	CY109896	CY109897	CY109899	CY109901	CY109902	H6N6	Anseriformes	Domestic	Fujian
A/duck/Shantou/494/2007	CY109735	CY109736	CY109737	CY109739	CY109741	CY109742	H6N6	Anseriformes	Domestic	Guangdong
A/chicken/Rizhao/1436/2013	KF260952	KF260708	KF260464	KF259976	KF259457	KF260220	H9N2	Galliformes	Domestic	Shandong
A/duck/Guangxi/890/2007	CY109599	CY109600	CY109601	CY109603	CY109605	CY109606	H6N8	Anseriformes	Domestic	Guangxi
A/chicken/Henan/13/2004	AY950282	AY950275	AY950268	AY950254	AY950240	AY950261	H5N1	Galliformes	Domestic	Henan
A/chicken/Henan/01/2004	AY950279	AY950272	AY950265	AY950251	AY950237	AY950258	H5N1	Galliformes	Domestic	Henan
A/duck/Hunan/908/2005	HM145698	HM145529	HM145360	HM145191	HM144853	HM145022	H6N2	Anseriformes	Domestic	Hunan
A/chicken/Wenzhou/606/2013	KF260933	KF260689	KF260445	KF259957	KF259438	KF260201	H9N2	Galliformes	Domestic	Zhejiang
A/duck/Guangxi/13/2004	DQ366335	DQ366336	DQ366337	DQ366339	DQ366341	DQ366342	H5N1	Anseriformes	Domestic	Guangxi
A/pigeon/Nanchang/2-0461/2000	CY005512	CY005511	CY005510	CY005509	CY005507	CY006020	H9N2	Passerine	Wild	Jiangxi

A/wild waterfowl/Dongting/PC2562/2012	KF972106	KF972107	KF972108	KF972110	KF972112	KF972113	H9N2	Anseriformes	Wild	Hunan
A/duck/Shantou/1522/2001	HM145593	HM145424	HM145255	HM145086	HM144749	HM144917	H6N2	Anseriformes	Domestic	Guangdong
A/duck/Fujian/3193/2005	HM145705	HM145536	HM145367	HM145198	HM144860	HM145029	H6N2	Anseriformes	Domestic	Fujian
A/duck/Fujian/3354/2006	CY109519	CY109520	CY109521	CY109523	CY109525	CY109526	H6N6	Anseriformes	Domestic	Fujian
A/duck/Hubei/2/2010	CY110946	CY110947	CY110948	CY110950	CY110952	CY110953	H6N6	Anseriformes	Domestic	Hubei
A/duck/Jiangxi/7348/2007	CY109703	CY109704	CY109705	CY109707	CY109709	CY109710	H6N2	Anseriformes	Domestic	Jiangxi
A/duck/Fujian/5643/2005	HM145711	HM145542	HM145373	HM145204	HM144866	HM145035	H6N2	Anseriformes	Domestic	Fujian
A/duck/Shantou/10124/2006	CY110339	CY110340	CY110341	CY110343	CY110345	CY110346	H6N2	Anseriformes	Domestic	Guangdong
A/chicken/Rizhao/723/2013	KF260946	KF260702	KF260458	KF259970	KF259451	KF260214	H9N2	Galliformes	Domestic	Shandong
A/chicken/Guangxi/G8/2009	KF367736	KF367737	KF367738	KF367739	KF367741	KF367742	H9N2	Galliformes	Domestic	Guangxi
A/chicken/Jiangxi/SD001/2013	CY146945	CY146946	CY146947	CY146949	CY146951	CY146952	H7N9	Galliformes	Domestic	Jiangxi
A/duck/Jiangxi/25186/2009	KF260741	KF260497	KF260253	KF259765	KF259246	KF260009	H7N6	Anseriformes	Domestic	Jiangxi
A/duck/Guangxi/1248/2006	CY109239	CY109240	CY109241	CY109243	CY109245	CY109246	H6N2	Anseriformes	Domestic	Guangxi
A/mallard/Jiangxi/8346/2004	HM145719	HM145550	HM145381	HM145212	HM144874	HM145043	H6N5	Anseriformes	Wild	Jiangxi
A/duck/Hunan/748/2005	HM145721	HM145552	HM145383	HM145214	HM144876	HM145045	H6N5	Anseriformes	Domestic	Hunan
A/duck/Jiangxi/16769/2010	KF260746	KF260502	KF260258	KF259770	KF259251	KF260014	H7N7	Anseriformes	Domestic	Jiangxi
A/duck/Hunan/573/2002	HM145645	HM145476	HM145307	HM145138	HM144800	HM144969	H6N2	Anseriformes	Domestic	Hunan
A/Muscovy duck/Fujian/CL/1997	JF916710	JF916711	JF916712	JF916714	JF916716	JF916717	H9N2	Anseriformes	Wild	Fujian
A/chicken/Jiangsu/1/00	DQ064561	DQ064534	DQ064507	DQ064453	DQ064399	DQ064480	H9N2	Galliformes	Domestic	Jiangsu
A/chicken/Hebei/4/2008	FJ499468	FJ499467	FJ499470	FJ499469	FJ499466	FJ499465	H9N2	Galliformes	Domestic	Hebei
A/wild duck/Hunan/211/2005	EU329189	EU329188	EU329187	EU329185	EU329183	EU329182	H5N1	Anseriformes	Wild	Hunan
A/duck/Zhejiang/2/2011	JQ906561	JQ906565	JQ906569	JQ906577	JQ906585	JQ906589	H7N3	Anseriformes	Domestic	Zhejiang
A/chicken/Guangdong/191/04	AY737286	AY737287	AY737288	AY737290	AY737292	AY737285	H5N1	Galliformes	Domestic	Guangdong
A/duck/Guizhou/5302/2007	CY109663	CY109664	CY109665	CY109667	CY109669	CY109670	H6N2	Anseriformes	Domestic	Guizhou
A/wild waterfowl/Dongting/C2032/2011	KF971946	KF971947	KF971948	KF971950	KF971952	KF971953	H9N2	Anseriformes	Wild	Hunan
A/chicken/Hubei/489/2004	AY770084	AY770083	AY770082	AY770081	AY770077	AY770080	H5N1	Galliformes	Domestic	Hubei
A/wild duck/Fujian/2/2011	JX534594	JX534595	JX534596	JX534598	JX534600	JX534601	H5N1	Anseriformes	Wild	Fujian
A/duck/Anhui/SC702/2013	CY147057	CY147058	CY147059	CY147061	CY147063	CY147064	H7N9	Anseriformes	Domestic	Anhui
A/mallard/SanJiang/151/2006	EF592492	EF592493	EF592494	EF592496	EF592498	EF592499	H6N2	Anseriformes	Wild	Guangxi
A/duck/Fujian/10774/2006	CY109575	CY109576	CY109577	CY109579	CY109581	CY109582	H6N6	Anseriformes	Domestic	Fujian

A/duck/Fujian/4276/2006	CY109535	CY109536	CY109537	CY109539	CY109541	CY109542	H6N2	Anseriformes	Domestic	Fujian
A/duck/Zhejiang/0607-13/2011	JN605401	JN605397	JN605393	JN605385	JN605377	JN605389	H1N2	Anseriformes	Domestic	Zhejiang
A/chicken/Wenzhou/645/2013	KF260919	KF260675	KF260431	KF259943	KF259424	KF260187	H7N7	Galliformes	Domestic	Zhejiang
A/duck/Hunan/S4111/2011	CY146657	CY146658	CY146659	CY146661	CY146663	CY146664	H9N2	Anseriformes	Domestic	Hunan
A/brown-headed gull/Qinghai/19/2009	GU477558	GU477555	GU477552	GU477546	GU477540	GU477537	H5N1	Charadriiformes	Wild	Qinghai
A/duck/Tibet/S2/2009	CY087184	CY087185	CY087186	CY087188	CY087190	CY087191	H9N2	Anseriformes	Domestic	Tibet
A/duck/Fujian/6388/2010	KF260726	KF260482	KF260238	KF259750	KF259231	KF259994	H7N3	Anseriformes	Domestic	Fujian
A/duck/Yangzhou/02/2005	EF061121	EF061124	EF061120	EF061123	EF061125	EF061119	H8N4	Anseriformes	Domestic	Jiangsu
A/duck/Guizhou/2773/2006	CY109303	CY109304	CY109305	CY109307	CY109309	CY109310	H6N2	Anseriformes	Domestic	Guizhou
A/chicken/Guangdong/174/04	AY609309	AY609310	AY609311	AY609313	AY609315	AY609316	H5N1	Galliformes	Domestic	Guangdong
A/duck/Hunan/S11893/2012	CY146593	CY146594	CY146595	CY146597	CY146599	CY146600	H4N6	Anseriformes	Domestic	Hunan
A/duck/Yangzhou/013/2008	GU220596	GU220597	GU220598	GU220600	GU220602	GU220603	H6N5	Anseriformes	Domestic	Jiangsu
A/wild duck/Jiangxi/19615/2009	KF260735	KF260491	KF260247	KF259759	KF259240	KF260003	H7N8	Anseriformes	Wild	Jiangxi
A/chicken/Henan/5/98	DQ064559	DQ064532	DQ064505	DQ064451	DQ064397	DQ064478	H9N2	Galliformes	Domestic	Henan
A/chicken/Shanghai/10/01	DQ064567	DQ064540	DQ064513	DQ064459	DQ064405	DQ064486	H9N2	Galliformes	Domestic	Shanghai
A/duck/Zhejiang/2245/2011	JN646697	JN646704	JN646711	JN646725	JN646739	JN646746	H5N1	Anseriformes	Domestic	Zhejiang
A/quail/Nanchang/7-026/2000	CY005460	CY005459	CY005458	CY005456	CY005454	CY005457	H3N6	Galliformes	Domestic	Jiangxi
A/wild duck/Shantou/992/2000	CY117380	CY117379	CY117378	CY117376	CY117374	CY117377	H2N8	Anseriformes	Wild	Guangdong
A/duck/Jiangxi/3190/2009	KF260716	KF260472	KF260228	KF259740	KF259221	KF259984	H7N9	Anseriformes	Domestic	Jiangxi
A/Baikal teal/Hongze/14/2005	GQ203121	GQ203120	GQ203119	GQ169501	GQ219716	GQ219715	H11N9	Anseriformes	Wild	Jiangsu
A/chicken/Ningxia/24/2006	HM172443	HM172375	HM172336	HM172241	HM172136	HM172285	H5N1	Galliformes	Domestic	Ningxia
A/duck/Hunan/S4150/2011	CY146689	CY146690	CY146691	CY146693	CY146695	CY146696	H5N1	Anseriformes	Domestic	Hunan
A/duck/Shantou/6847/2004	HM145669	HM145500	HM145331	HM145162	HM144824	HM144993	H6N2	Anseriformes	Domestic	Guangdong
A/pigeon/Shanghai/S1421/2013	CY147177	CY147178	CY147179	CY147181	CY147183	CY147184	H7N9	Passerine	Wild	Shanghai

Sequence name, subtype, host order, host type and region types are summarized in each column.

(page intentionally blank)

Table S10: Original predictor data per province

Predictor data are referenced from the China statistical year book 2013 and the China agriculture yearbook 2012. The table is separated into three parts for display. The predictor name keys (and units) are listed at the end.

Province	Agriculture								
	1	2	3	4	5	6	7	8	9
Beijing	20.09	15.24	15.87	2662.8	1584.34	1700	1700	154.16	3658.52
Tianjin	0.73	18.66	11.87	2315.5	2049.12	612	612	105.01	6485.19
Hebei	3.86	342.56	99.91	35668.3	1877.28	2466	431	1747.66	9628.07
Shanxi	2.77	74.69	10.25	6282.1	305.25	1435	482	298.83	5101.21
Inner Mongolia	1.92	54.46	32.02	4735.5	40.03	2493	774	1118.86	25875.66
Liaoning	28.78	279.9	137.43	39152.2	2687.18	4594	1338	1621.23	17585.47
Jilin	10.7	100.25	78.19	15302.8	816.58	1818	824	1130.36	21768.51
Heilongjiang	3.42	108.15	36.04	13715.9	289.98	2192	759	1350.68	28180.14
Shanghai	23.33	5.9	6.48	1420	2239.75	4043	4043	72.59	2015.71
Jiangsu	8.73	197.2	156.59	34741.9	3386.15	9486	1255	1226.18	5337.15
Zhejiang	12.35	48.14	38.26	12416.4	1219.69	8446	2320	549.04	6934.74
Anhui	19.87	122.65	115.35	23906	1712.46	1992	533	1119.73	10392.83
Fujian	3.77	25.36	40.88	8424.2	693.92	1817	306	481.28	6741.12
Jiangxi	6.2	45.79	60.6	19451.6	1165.46	1656	502	752.68	6937.5
Shandong	37.72	401.99	287.41	58330.5	3715.32	2472	462	2285.92	12157.98
Henan	5.87	404.17	139.66	64642	3870.78	2463	887	2255.61	8957.36
Hubei	2.8	139.36	67.91	29812.3	1603.67	2217	825	1334.04	7446.51
Hunan	2.1	95.18	60.56	27563.8	1301.41	2331	948	1488.58	5179.18
Guangdong	10.81	31.81	159.26	36998.9	2057.78	8836	1660	1134.14	4342.63
Guangxi	7.77	21.82	141.43	30282.6	1281.53	1741	512	1072.77	8992.57
Hainan	16.68	3.58	27.87	4666.7	1375.8	431	124	214.14	8829.15
Chongqing	8.63	40.05	40.64	11627.5	1400.9	1735	1735	453.9	9653.81
Sichuan	3.97	146.44	120.5	37519.8	773.6	4151	2129	2269.86	10218.22
Guizhou	2.45	14.65	17.57	7698.1	436.9	1128	417	421.55	7956.99
Yunnan	2.21	22.13	39.19	11949.6	303.29	1657	736	912.97	14409
Tibet	0.09	0.43	0.17	122.6	1	131	48	59.02	43715.73
Shaanxi	1.39	51.86	9.29	6255	303.94	1335	600	598.72	5888.58
Gansu	1.13	14.7	6.59	3676.6	80.8	1322	399	231.72	13124.41
Qinghai	0.08	2.01	1.08	242.2	3.36	561	326	137.08	15663.33
Ningxia	2.37	6.18	2.5	884.5	133.21	1126	573	105.72	16077.68
Xinjiang	4.27	25.89	19.91	2964.6	17.81	1479	633	485.37	29085.38

Province	Resource and Environment										
	10	11	12	13	14	15	16	17	18	19	20
Beijing	1038.58	7.97	12.85	51	733.2	2450.2	39.5	17.95	6.68	9.38	17.75
Tianjin	198.89	8.06	12.53	57	755.3	2174.4	32.94	26.54	8.41	22.45	33.42
Hebei	8374.08	3.61	13.96	54.67	649.4	2288.2	235.53	117.76	123.59	134.12	176.11
Shanxi	7643.67	7.44	10.65	51.17	427.8	2618.6	106.25	65.9	107.09	130.18	124.4
Inner Mongolia	117720.51	11.57	7.15	46.83	551.4	2677.8	510.25	349.24	83.3	138.49	141.89
Liaoning	20226.85	12.39	7.43	68.5	786	2577.2	547.3	492.42	72.63	105.87	103.63
Jilin	84412.29	12.43	5.23	63	718.3	2438.3	460.47	387.33	26.48	40.35	57.59
Heilongjiang	152104.96	14.85	4.62	67.17	740.8	1773.8	841.41	695.69	69.93	51.43	78.06
Shanghai	100.95	5.22	16.87	69.5	1103.7	1676.7	33.9	27.35	8.71	22.82	40.16
Jiangsu	3501.75	4.1	15.98	68.08	917.2	1939.1	373.33	279.14	44.32	99.2	147.96
Zhejiang	17223.14	1.53	17.11	70.75	1728.8	1520.5	1444.79	1427.15	25.4	62.58	80.88
Anhui	13755.41	3.76	16.49	73.08	936.4	1912.8	701	640.64	46.21	51.96	92.13
Fujian	48436.28	3.11	20.18	75.17	1913.4	1291.3	1511.44	1510.07	25.26	37.13	46.72
Jiangxi	39529.64	7.55	18.03	77.08	2059.8	1622	2174.36	2155.79	35.74	56.77	57.71
Shandong	6338.53	4.71	14.34	55.17	569.1	2146	274.3	182.17	69.53	174.88	173.9
Henan	12936.12	4.4	15.46	53.17	498.7	1883.7	265.54	172.65	59.98	127.59	162.59
Hubei	20942.49	5.14	16.37	81.42	1415.5	1553.9	813.88	783.76	34.97	62.24	64
Hunan	34906.67	6.07	17.59	76.42	1730	1493.6	1988.94	1981.31	34.07	64.5	60.72
Guangdong	30183.37	6.73	21.67	81.58	1813.9	1471.2	2026.55	2017.49	32.83	79.92	130.34
Guangxi	46875.18	5.98	21.42	79.75	1086.8	1295.7	2087.4	2086.36	29.97	50.41	49.83
Hainan	7274.23	6.97	24.58	81.92	2094.3	1766.4	364.31	360.22	1.66	3.41	10.34
Chongqing	11331.85	10.32	18.3	71.75	1104.4	812	476.89	476.89	18.23	56.48	38.27
Sichuan	159572.37	18.54	15.86	78.08	610.9	780.6	2892.36	2891.21	29.58	86.44	65.9
Guizhou	24007.96	5.41	13.65	84.58	1226.4	681.6	974.02	974.02	29.45	104.11	56.35
Yunnan	155380.09	7.45	16.33	66.83	802.1	2554.2	1689.77	1689.77	39.06	67.22	54.43
Tibet	224550.91	33.91	9.61	33.5	365.2	3162.9	4196.35	4196.35	0.66	0.42	4.43
Shaanxi	33820.54	5.65	14.17	61.75	385.3	1922	390.49	367.96	46.21	84.38	80.81
Gansu	19363.83	16.17	7.54	57.42	231.2	2600.2	266.95	258.95	20.76	57.25	47.34
Qinghai	3915.64	30.21	5.22	59	446.1	2655.2	895.22	879.21	15.64	15.39	12.61
Ningxia	492.14	10.34	9.81	48	292.7	2728.3	10.81	8.45	19.83	40.66	45.54
Xinjiang	30100.54	12.95	7.39	53.08	286.9	2864.6	900.63	851.6	69.61	79.61	81.95

Province	Population							Transportation			
	21	22	23	24	25	26	27	28	29	30	31
Beijing	2069.3	1231.213	1783.737	86.2	285.5634	13.8	1231.21	26161.91	1236.91	24925	0
Tianjin	1413.15	1250.575	1152.424	86.2	260.7262	18.45	1250.58	46015.2	7909.2	27735	10371
Hebei	7287.51	383.5532	3410.555	86.2	3876.955	53.2	383.55	219130.28	21010.28	195530	2590
Shanxi	3610.83	175.4534	1850.911	86.2	1759.919	48.74	175.45	144607.93	71427.93	73150	30
Inner Mongolia	2489.85	21.04691	1437.639	86.2	1052.211	42.26	21.05	189942.25	64682.25	125260	0
Liaoning	4389	301.2354	2881.379	65.65	1507.622	34.35	301.24	206788.68	19802.68	174355	12631
Jilin	2750.4	146.7663	1476.965	53.7	1273.435	46.3	146.77	54808.12	7347.12	47130	331
Heilongjiang	3834	81.05708	2181.546	56.9	1652.454	43.1	81.06	65230.66	16590.66	47465	1175
Shanghai	2380.43	3754.621	2125.724	89.3	254.706	10.7	3754.62	94038.29	825.29	42911	50302
Jiangsu	7919.98	771.9279	4989.587	63	2930.393	37	771.93	220007.48	7670.48	153698	58639
Zhejiang	5477	538.0157	3461.464	63.2	2015.536	36.8	538.02	191817.32	4607.32	113393	73817
Anhui	5988	428.9398	2784.42	46.5	3203.58	53.5	428.94	312436.77	12259.77	259461	40716
Fujian	3748	308.7315	2233.808	59.6	1514.192	40.4	308.73	84345.09	3814.09	59431	21100
Jiangxi	4503.93	269.8581	2139.818	47.51	2364.114	52.49	269.86	127195.53	5561.53	113703	7931
Shandong	9684.97	616.8771	5077.83	52.43	4607.14	47.57	616.88	333602.6	23144.6	296754	13704
Henan	9406	563.2335	3990.966	42.43	5415.034	57.57	563.23	272114.94	12637.94	251772	7705
Hubei	5779	310.8661	3091.765	53.5	2687.235	46.5	310.87	122945.35	5882.35	97136	19927
Hunan	6638.93	313.4528	3097.061	46.65	3541.869	53.35	313.45	191051.75	5676.75	166670	18705
Guangdong	10594	589.2102	7140.356	67.4	3453.644	32.6	589.21	256076.69	9305.69	189034	57737
Guangxi	4682	198.138	2038.075	43.53	2643.925	56.47	198.14	161356.01	6846.01	135112	19398
Hainan	886.55	261.365	457.4598	51.6	429.0902	48.4	261.36	26880.39	752.39	16600	9528
Chongqing	2945	354.8193	1678.061	56.98	1266.939	43.02	354.82	86474.05	2328.05	71272	12874
Sichuan	8076.2	166.5196	3515.57	43.53	4560.63	56.47	166.52	174349.34	8793.34	158396	7160
Guizhou	3484.07	197.7338	1268.55	36.41	2215.52	63.59	197.73	52654.92	6664.92	44892	1098
Yunnan	4659	118.2487	1831.453	39.31	2827.547	60.69	118.25	68734.88	5030.88	63239	465
Tibet	307.62	2.504233	69.98355	22.75	237.6365	77.25	2.5	1126.63	84.63	1042	0
Shaanxi	3753.09	182.3659	1877.321	50.02067	1875.769	49.97933	182.37	136726.79	31941.79	104593	192
Gansu	2577.55	56.64945	998.8006	38.75	1578.749	61.25	56.65	45831.67	6289.67	39517	25
Qinghai	573.17	7.960694	271.9118	47.44	301.2582	52.56	7.96	13483.92	3783.92	9700	0
Ningxia	647.19	97.46837	327.9312	50.67	319.2588	49.33	97.47	41113.31	8467.31	32646	0
Xinjiang	2232.78	13.41	981.98	43.98	1250.8	56.02	13.41	58793.53	6839.53	51954	0

Predictor names key

1 Sales of Poultry Per Capita Rural Household (kg)
2 Output of Poultry Eggs (10 000 tons)
3 Output of Poultry meat (10 000 tons)
4 poultry population at the end of year (10000 unit)
5 poultry density (10000unit/km^2))
6 number of farm product market (region) (unit)
7 number of farm product market (city) (unit)
8 Gross Output Value of Animal Husbandry (100 million yuan)
9 Original Value of Productive Fixed Assets of Rural Households at Year-end ()
10 Stock Volume of Forest (10 000 cu.m)
11 Percentage of Nature Reserves in the Region (%)
12 Average Temperature of Major Cities (℃)
13 Average Relative Humidity of Major Cities (%)
14 Average Precipitation of Major Cities (milimeters)
15 Average Sunshine Hours of Major Cities (Hours)
16 Total Amount of Water Resources (100 million cu.m)
17 Surface Water Resources (100 million cu.m)
18 Smoke and Dust (10 000 tons)
19 Sulphur Dioxide (10 000 tons)
20 Nitrogen Oxides (10 000 tons)
21 Population at Year-end by Region (10 000 persons)
22 population density ()
23 Urban Population (Population)
24 Urban Population (%)
25 Rural Population (Population)
26 Rural Population (%)
27 population density (10000 persons/km^2))
28 Freight Traffic by Region total (10 000 tons)
29 Freight Traffic by Region railway (10 000 tons)
30 Freight Traffic by Region Highways (10 000 tons)
31 Freight Traffic by Region Waterways (10 000 tons)
