# Supplementary material for: Quantifying predictors for the spatial diffusion of avian influenza virus in China
Source: BMC Evol Biol. 2017 Jan 13;17:16. doi: 10.1186/s12862-016-0845-3 (PMC5237338; doi:10.1186/s12862-016-0845-3)
Supplement: Additional file 2: Figure S1. — Host distribution of 320 Chinese AIV sequences in Traditional Region, Economic Region, Economic Divided zone, and China Agro-Ecological Region types. Figure S2. Distributions of 320 Chinese AIV sequences in the sampled time, host order, subtype and sampled provinces. Figure S3. Influence of the sampling scheme on the phylogeographic reconstruction. Figure S4. PB1 tree mapping with region traits. Figure S5. PA tree mapping with region traits. Figure S6. NP tree mapping with region traits. Figure S7. M tree mapping with region traits. Figure S8. NS tree mapping with region traits. (PDF 864 kb) [file 12862_2016_845_MOESM2_ESM.pdf]

Additional Figures

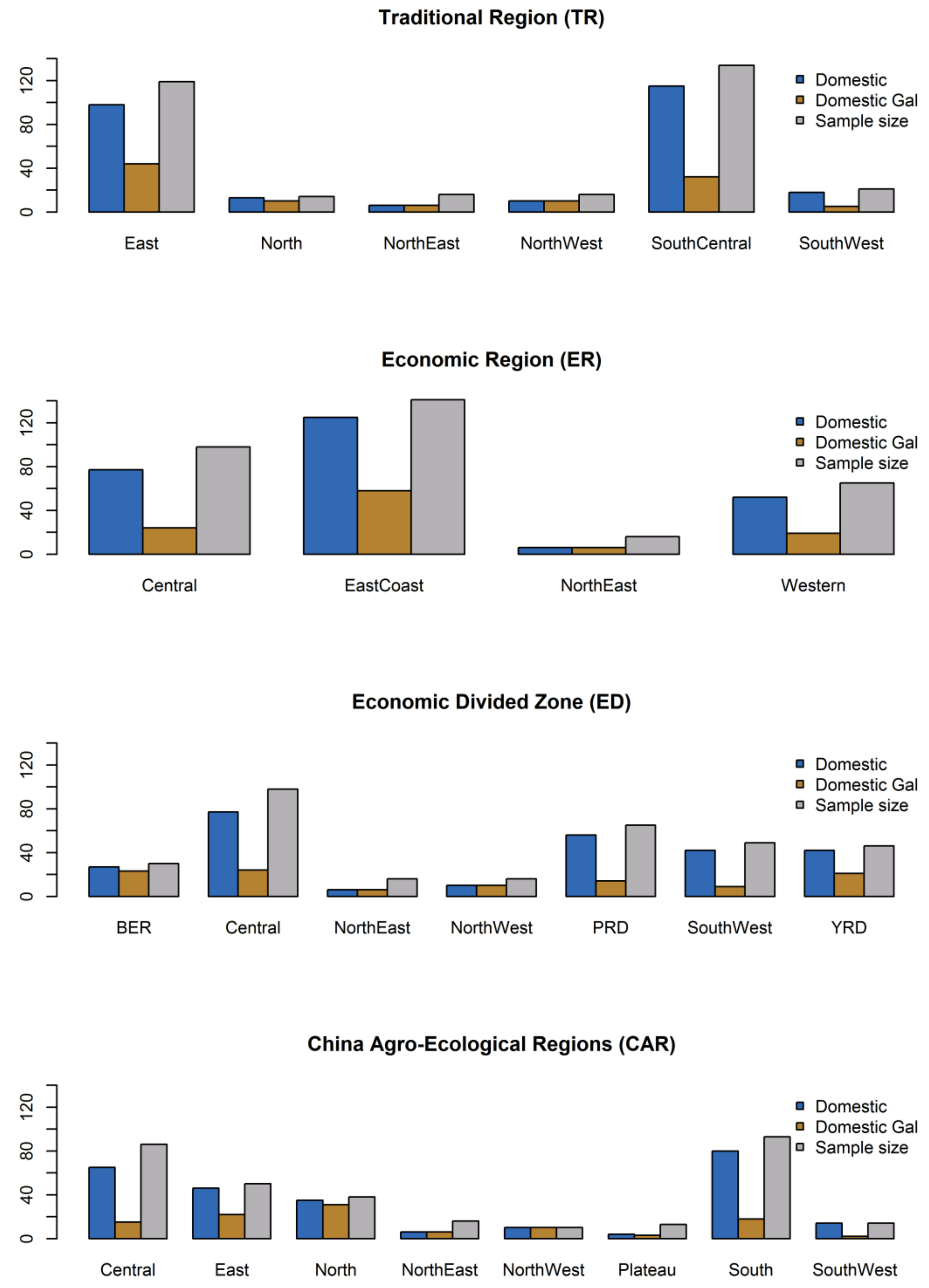

Figure S1 Host distribution of 320 Chinese AIV sequences in Traditional Region, Economic Region, Economic Divided Zone, and China Agro-Ecological Region types.
The number of domestic bird sequences per region trait is shown in blue; domestic galliformes per region trait is shown in orange and the number of samples per region trait is shown in grey.

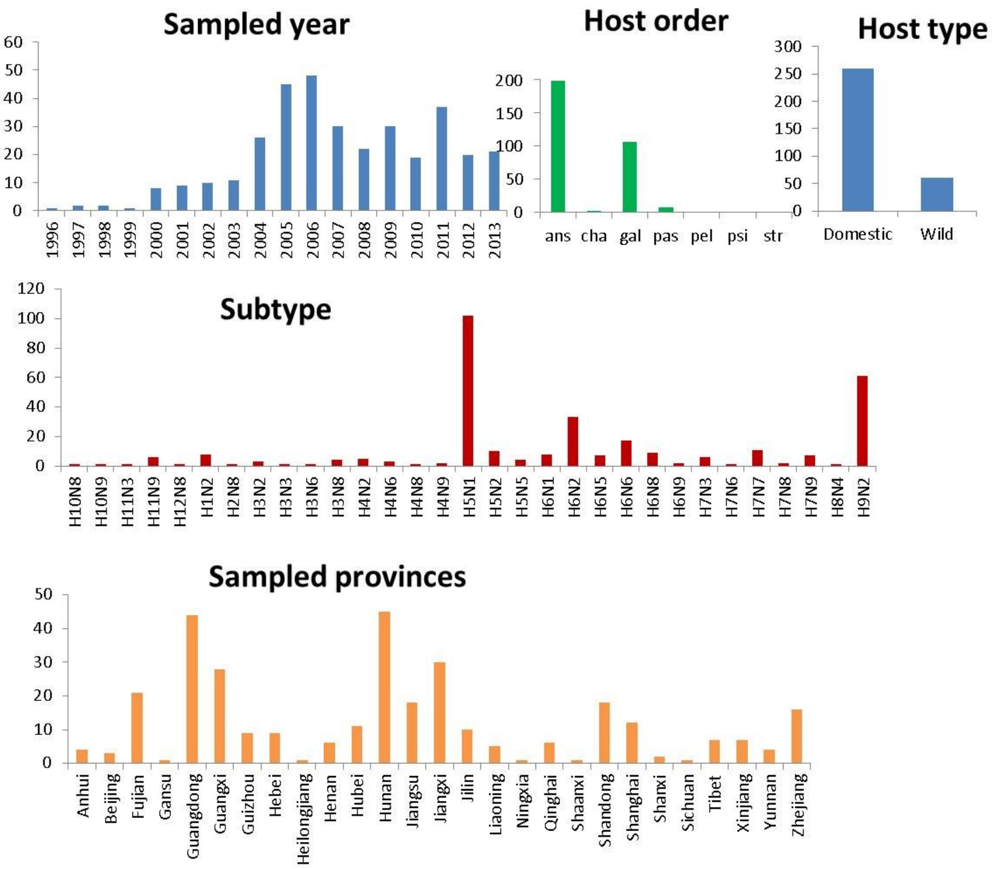

Figure S2 Distributions of 320 Chinese AIV sequences in the sampled time, host order, subtype and sampled provinces.

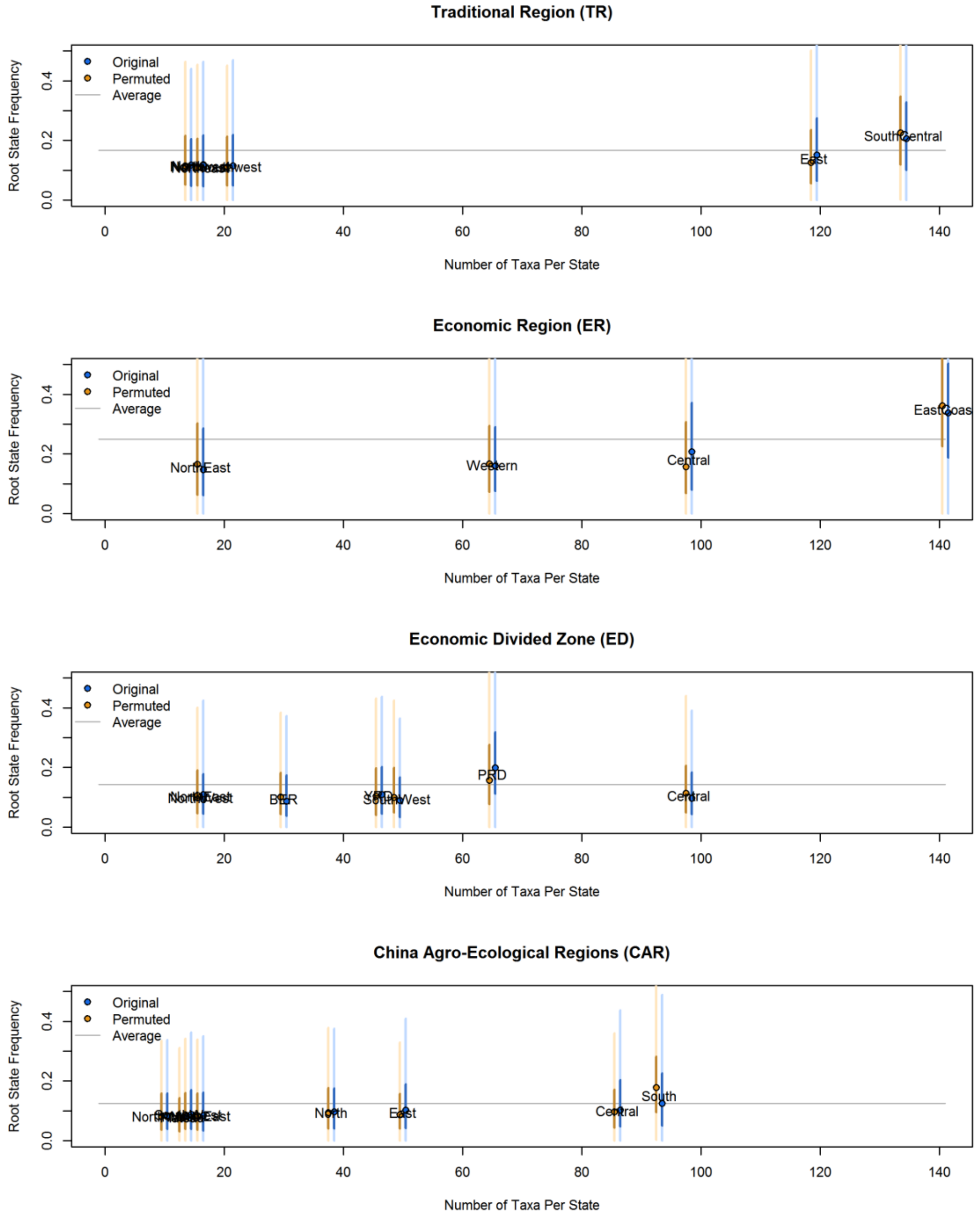

Figure S3 Influence of the Sampling Scheme on the phylogeographic reconstruction. Blue points are the estimated root state frequencies (median, with interquartile range-bold, and 95% HPD intervals-pale) against the number of taxa per location state using the original data (with state name under each point) in 4 region types: Traditional Regions (TR), Economic Regions (ER), Economic Development Zones (ED) and China Agro-Ecological Regions (CAR); Orange points are the estimated root state frequencies against the number of taxa with randomizes location state assignment.

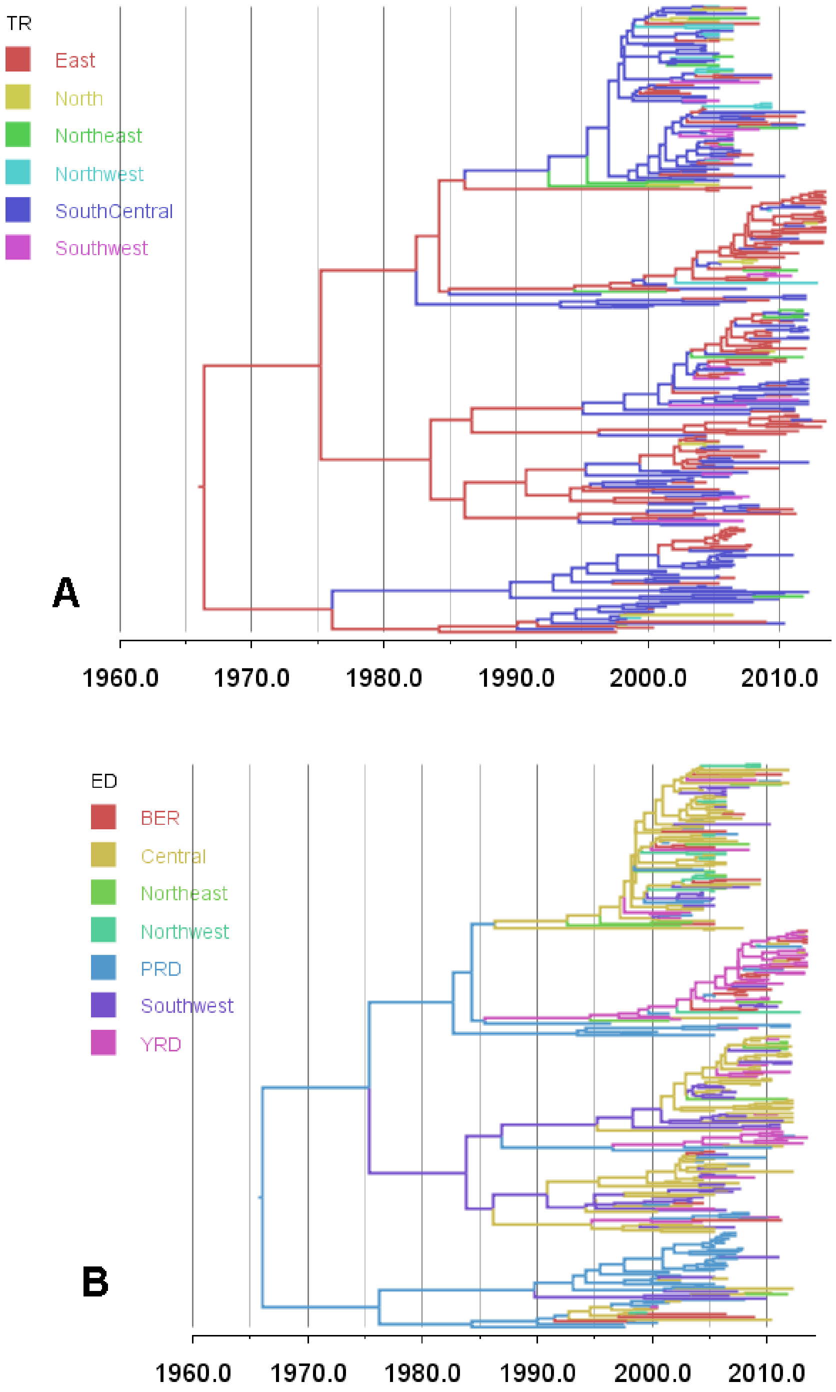

Figure S4 Bayesian MCC phylogenies of PB1 segment.
A: PB1 phylogeny, coloured by traditional regions
B: PB1 phylogeny coloured by economic zones
Branches are coloured according to their descendent nodes annotated by the different sampled areas within the certain region type, with the key for colours shown on the left.

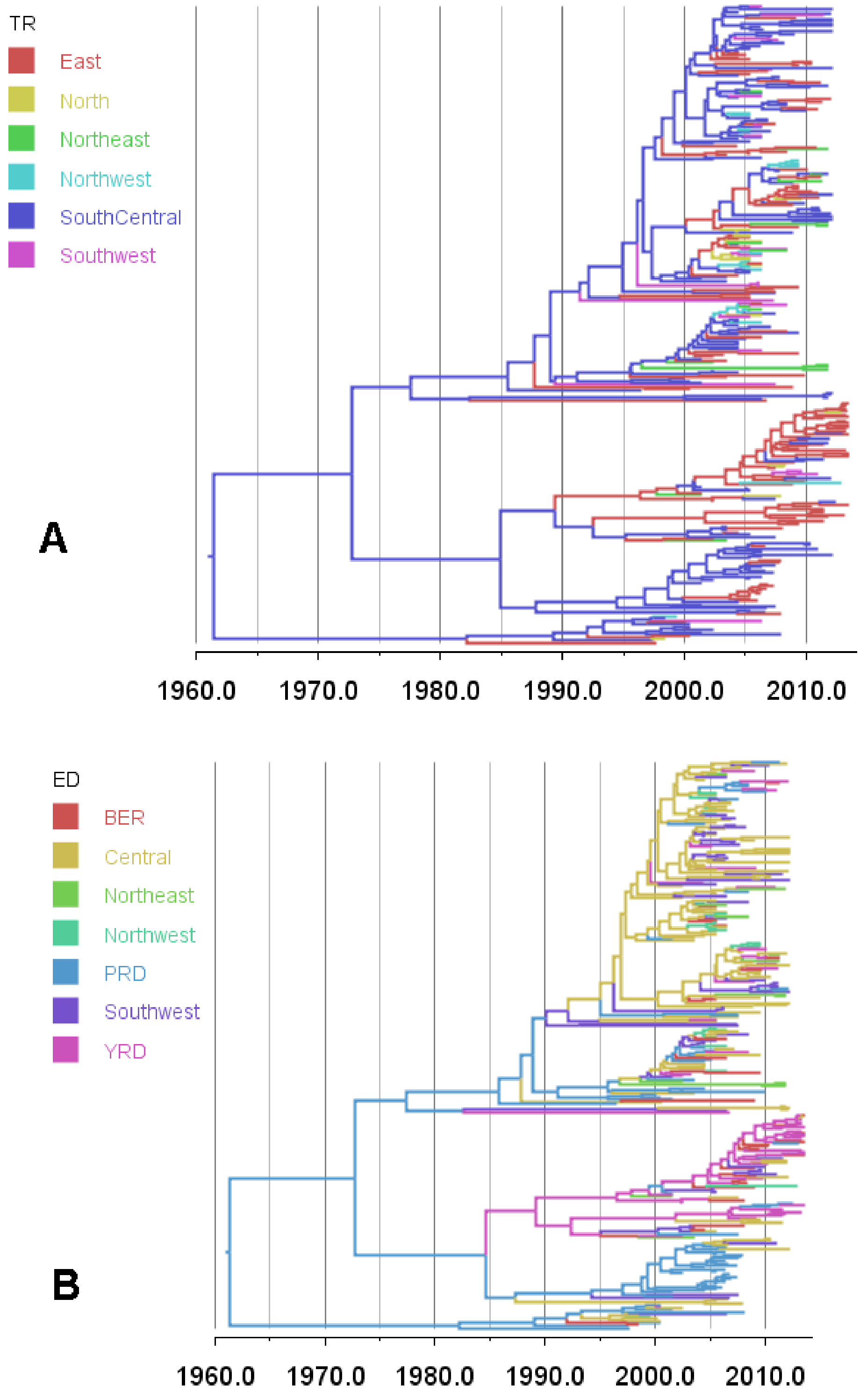

Figure S5 Bayesian MCC phylogenies of PA segment.
A: PA phylogeny, coloured by traditional regions
B: PA phylogeny coloured by economic zones
Branches are coloured according to their descendent nodes annotated by the different sampled areas within the certain region type, with the key for colours shown on the left.

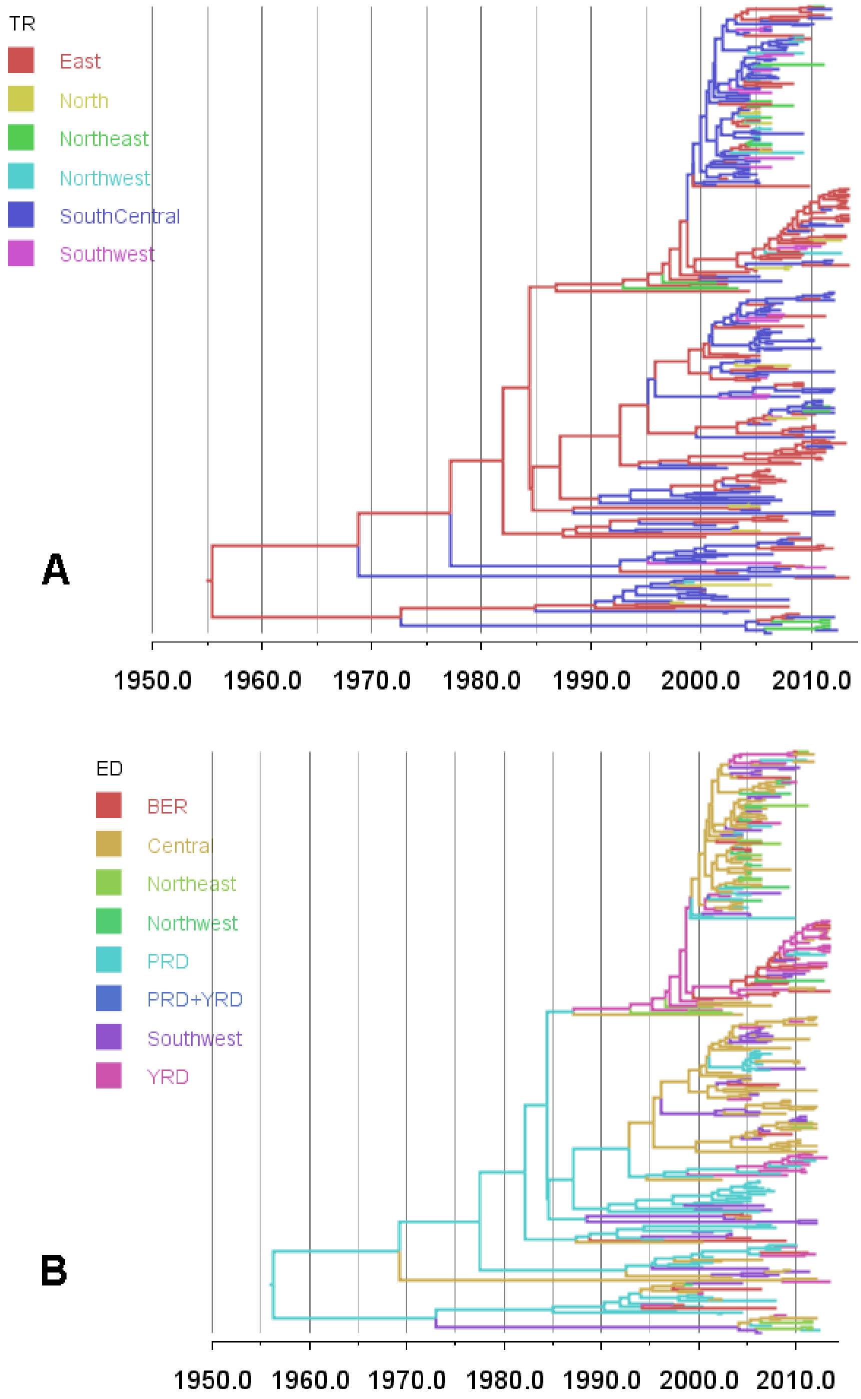

Figure S6 Bayesian MCC phylogenies of NP segment.
A: NP phylogeny, coloured by traditional regions
B: NP phylogeny coloured by economic zones
Branches are coloured according to their descendent nodes annotated by the different sampled areas within the certain region type, with the key for colours shown on the left.

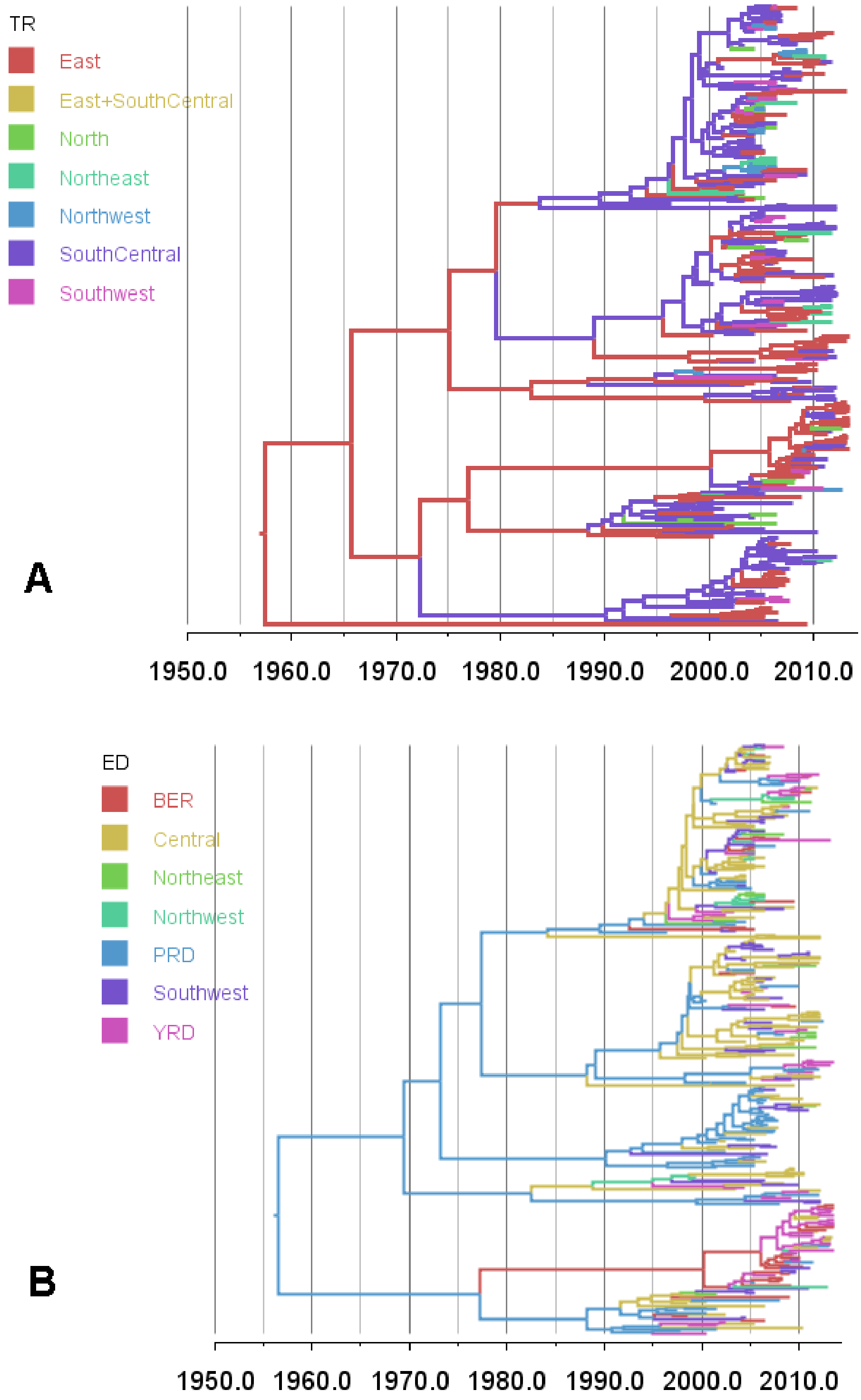

Figure S7 Bayesian MCC phylogenies of M segment.
A: M phylogeny, coloured by traditional regions
B: M phylogeny coloured by economic zones
Branches are coloured according to their descendent nodes annotated by the different sampled areas within the certain region type, with the key for colours shown on the left.

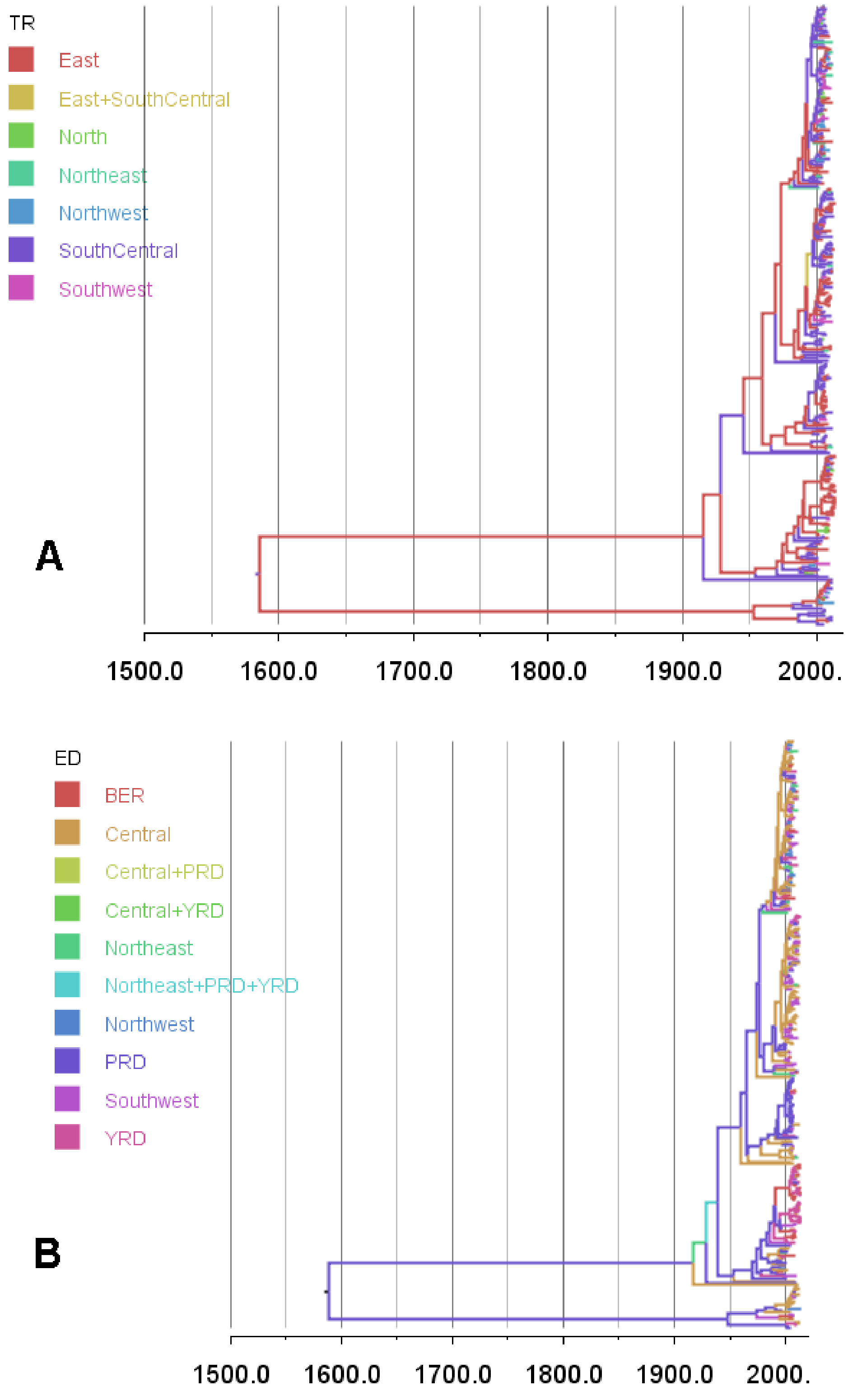

Figure S8 Bayesian MCC phylogenies of NS segment.
A: NS phylogeny, coloured by traditional regions
B: NS phylogeny coloured by economic zones
Branches are coloured according to their descendent nodes annotated by the different sampled areas within the certain region type, with the key for colours shown on the left.
