# Supplementary figures and images for: Quantifying predictors for the spatial diffusion of avian influenza virus in China
Source: BMC Evol Biol. 2017 Jan 13;17:16. doi: 10.1186/s12862-016-0845-3 (PMC5237338; doi:10.1186/s12862-016-0845-3)

Figure S9 (A)

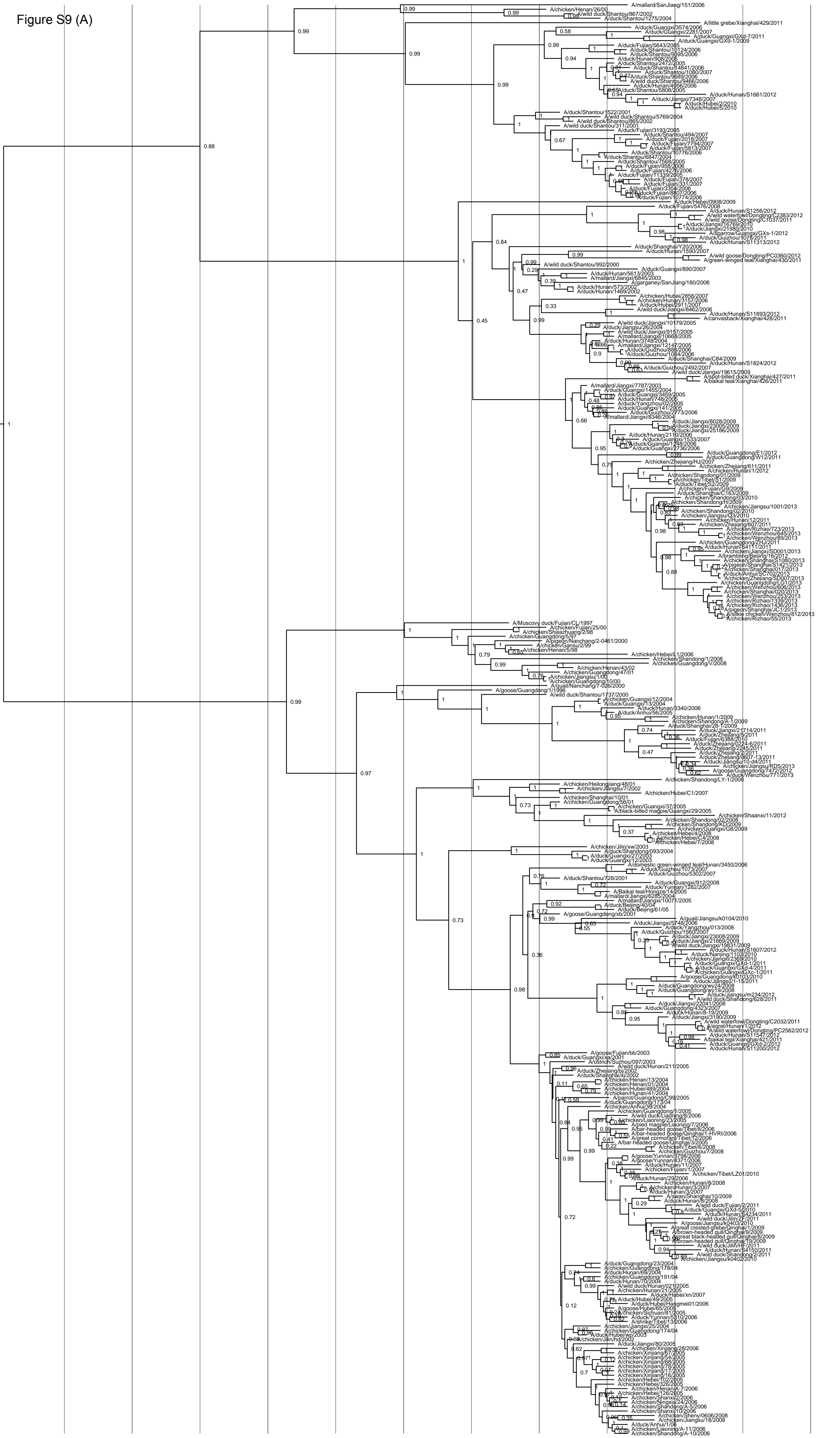

Supplement: Additional file 3: Figure S9. — (A to F) Bayesian MCC phylogenies of 6 internal segments of 320 Chinese AIV sequences labelled with sequence names on tips and Bayesian posterior probability on nodes. (A) PB2; (B) PB1; (C) PA; (D) NP; (E) M; (F) NS. (ZIP 523 kb) [file 12862_2016_845_MOESM3_ESM.zip › Figure S9 A (PB2).pdf]

Figure S9 (B)

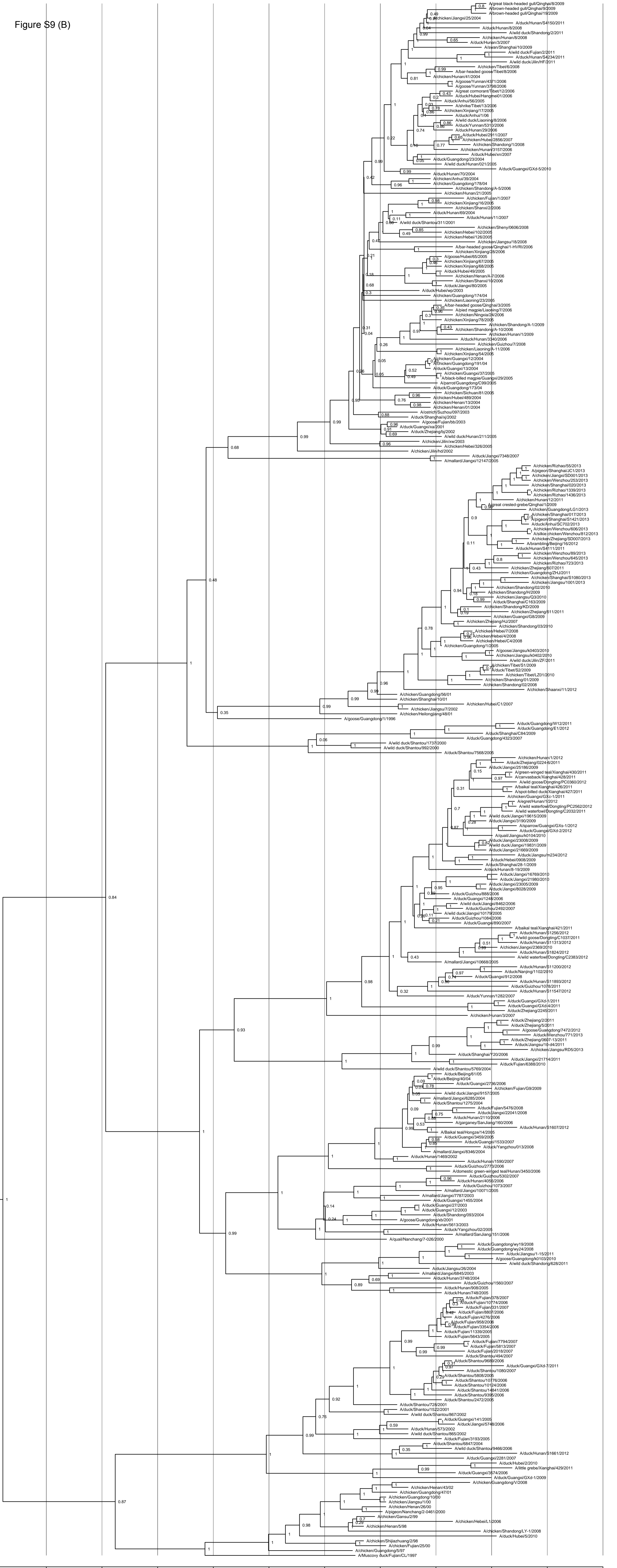

Supplement: Additional file 3: Figure S9. — (A to F) Bayesian MCC phylogenies of 6 internal segments of 320 Chinese AIV sequences labelled with sequence names on tips and Bayesian posterior probability on nodes. (A) PB2; (B) PB1; (C) PA; (D) NP; (E) M; (F) NS. (ZIP 523 kb) [file 12862_2016_845_MOESM3_ESM.zip › Figure S9 B (PB1).pdf]

Figure S9 (C)

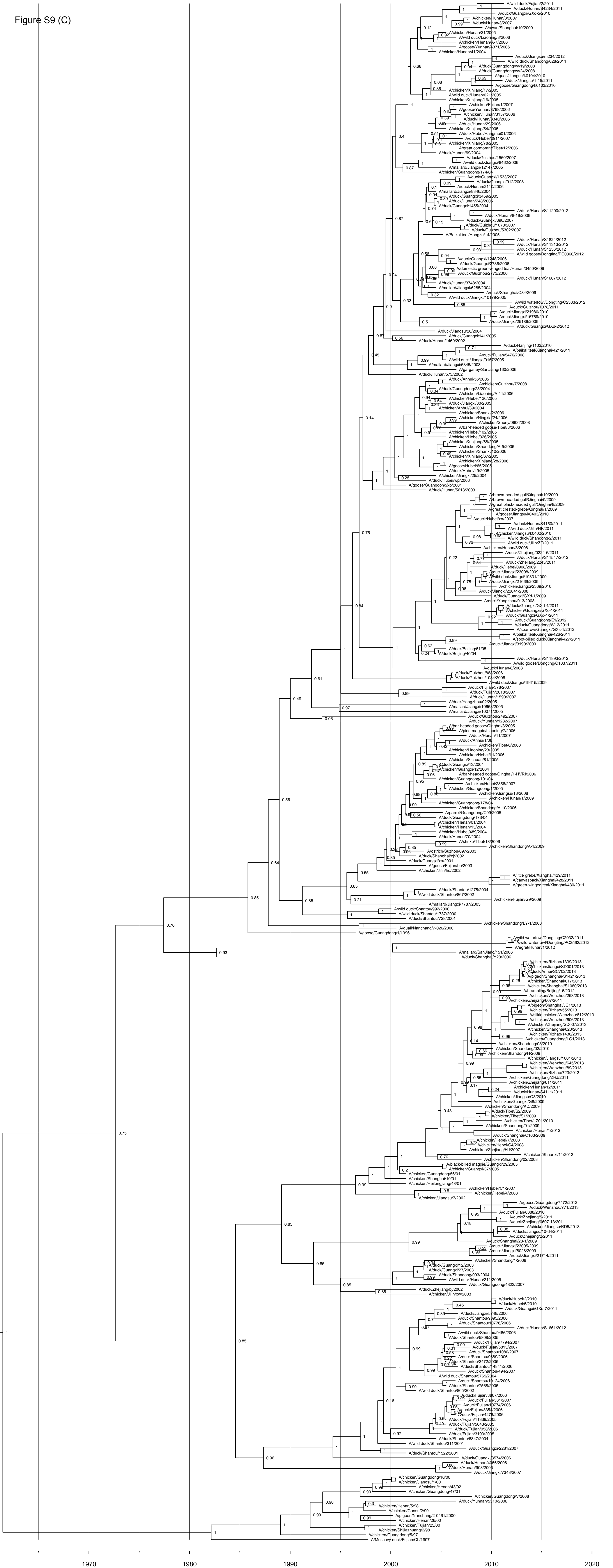

Supplement: Additional file 3: Figure S9. — (A to F) Bayesian MCC phylogenies of 6 internal segments of 320 Chinese AIV sequences labelled with sequence names on tips and Bayesian posterior probability on nodes. (A) PB2; (B) PB1; (C) PA; (D) NP; (E) M; (F) NS. (ZIP 523 kb) [file 12862_2016_845_MOESM3_ESM.zip › Figure S9 C (PA).pdf]

Figure S9 (D)

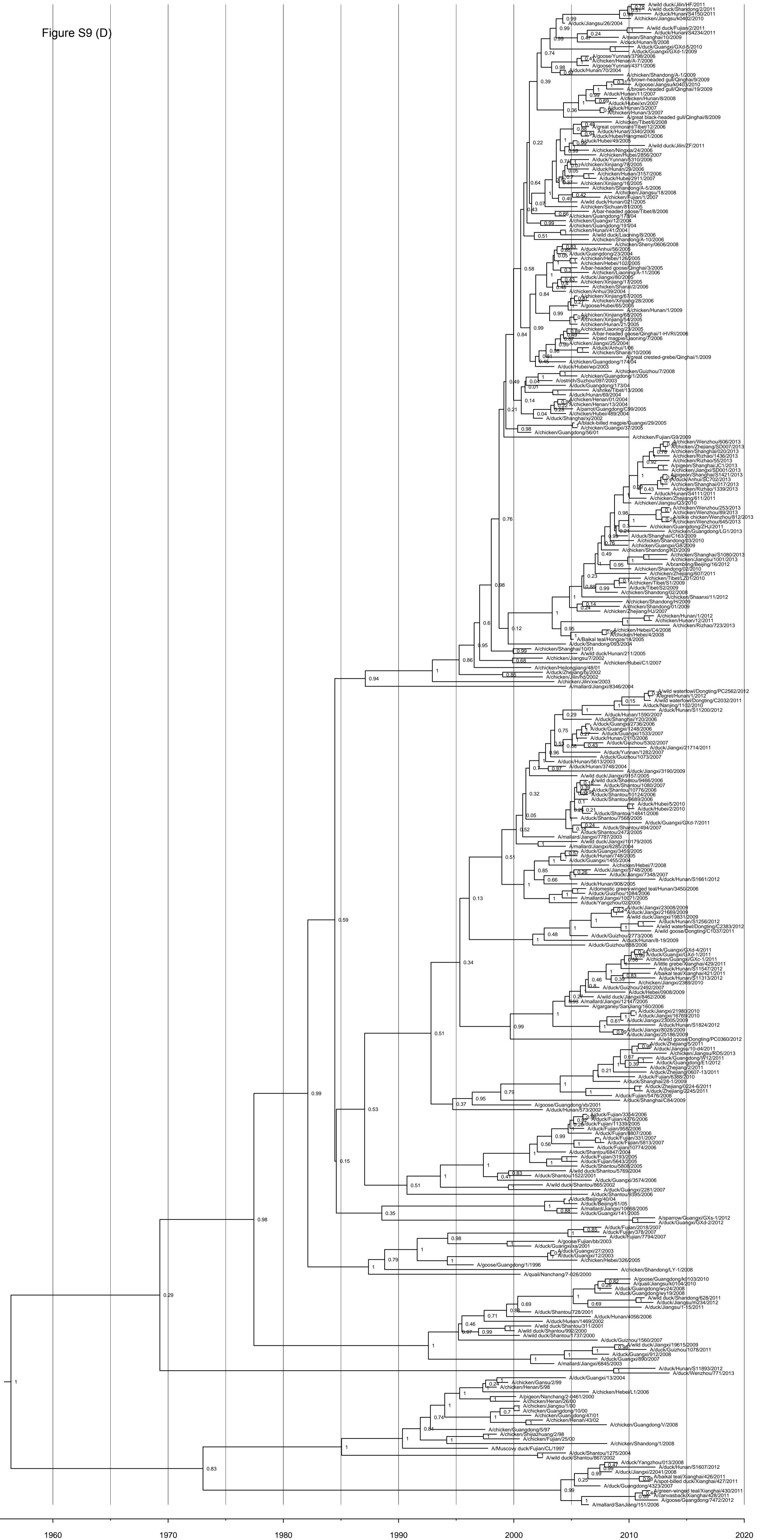

Supplement: Additional file 3: Figure S9. — (A to F) Bayesian MCC phylogenies of 6 internal segments of 320 Chinese AIV sequences labelled with sequence names on tips and Bayesian posterior probability on nodes. (A) PB2; (B) PB1; (C) PA; (D) NP; (E) M; (F) NS. (ZIP 523 kb) [file 12862_2016_845_MOESM3_ESM.zip › Figure S9 D (NP).pdf]

Figure S9 (E)

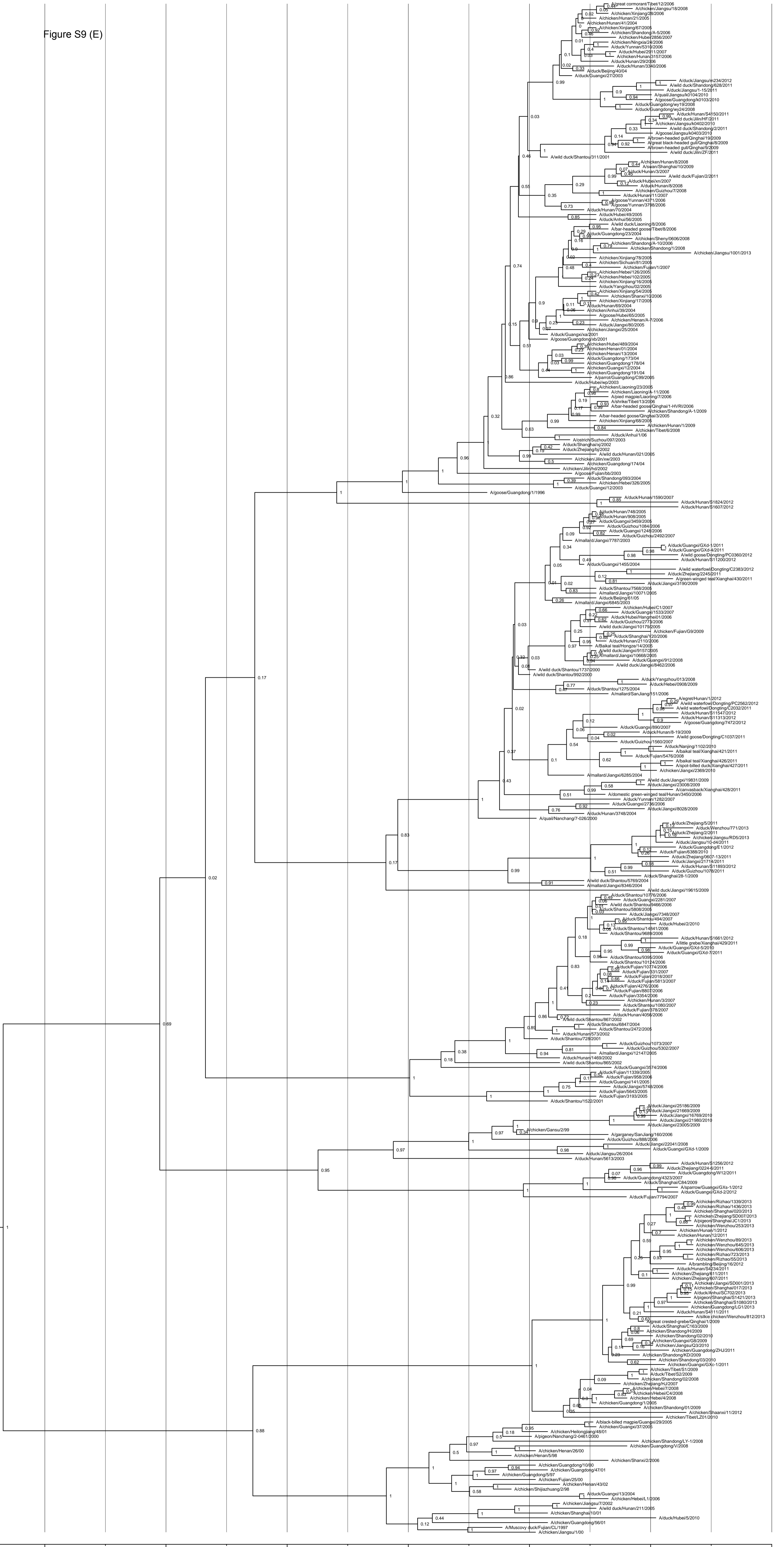

Supplement: Additional file 3: Figure S9. — (A to F) Bayesian MCC phylogenies of 6 internal segments of 320 Chinese AIV sequences labelled with sequence names on tips and Bayesian posterior probability on nodes. (A) PB2; (B) PB1; (C) PA; (D) NP; (E) M; (F) NS. (ZIP 523 kb) [file 12862_2016_845_MOESM3_ESM.zip › Figure S9 E (M).pdf]

Figure S9 (F)

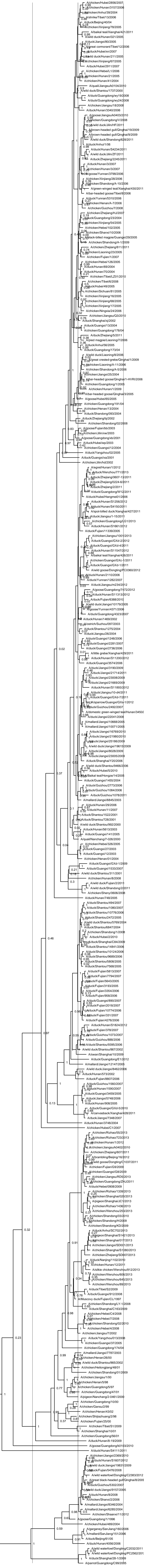

Supplement: Additional file 3: Figure S9. — (A to F) Bayesian MCC phylogenies of 6 internal segments of 320 Chinese AIV sequences labelled with sequence names on tips and Bayesian posterior probability on nodes. (A) PB2; (B) PB1; (C) PA; (D) NP; (E) M; (F) NS. (ZIP 523 kb) [file 12862_2016_845_MOESM3_ESM.zip › Figure S9 F (NS).pdf]
